# Discovery of dihydrooxazolo[2,3-*a*]isoquinoliniums as highly specific inhibitors of hCE2†

**DOI:** 10.1039/c9ra07457k

**Published:** 2019-11-04

**Authors:** Lixia Ding, Lu Wang, Kun Zou, Bo Li, Yunqing Song, Qihua Zhang, Yitian Zhao, Zhijian Xu, Guangbo Ge, Bo Zhao, Weiliang Zhu

**Affiliations:** Key Laboratory of Receptor Research, Drug Discovery and Design Center, Shanghai Institute of Materia Medica, Chinese Academy of Sciences 555 Zuchongzhi Road Shanghai 201203 China boli@simm.ac.cn wlzhu@simm.ac.cn; Shanghai University of Traditional Chinese Medicine 1200 Cailun Road Shanghai 201203 China geguangbo@dicp.ac.cn; College of Chemistry and Materials Science, Nanjing Normal University 1 Wenyuan Road Nanjing 210097 China zhaobo@njnu.edu.cn; University of Chinese Academy of Sciences No. 19A Yuquan Road Beijing 100049 China; Open Studio for Druggability Research of Marine Natural Products, Pilot National Laboratory for Marine Science and Technology (Qingdao) 1 Wenhai Road, Aoshanwei, Jimo Qingdao 266237 China

## Abstract

Human carboxylesterase 2 (hCE2) is one of the most abundant esterases distributed in human small intestine and colon, which participates in the hydrolysis of a variety of ester-bearing drugs and thereby affects the efficacy of these drugs. Herein, a new compound (23o) with a novel skeleton of dihydrooxazolo[2,3-*a*]isoquinolinium has been discovered with strong inhibition on hCE2 (IC_50_ = 1.19 μM, *K*_i_ = 0.84 μM) and more than 83.89 fold selectivity over hCE1 (IC_50_ > 100 μM). Furthermore, 23o can inhibit hCE2 activity in living HepG2 cells with the IC_50_ value of 2.29 μM, indicating that this compound has remarkable cell-membrane permeability and is capable for inhibiting intracellular hCE2. The SAR (structure–activity relationship) analysis and molecular docking results demonstrate that the novel skeleton of oxazolinium is essential for hCEs inhibitory activity and the benzyloxy moiety mainly contributes to the selectivity of hCE2 over hCE1.

## Abstract 2

Novel oxazoliniums are highly specific inhibitors of hCE2 over hCE1 and have good cell-membrane permeability for inhibiting intracellular hCE2.

## Graphical abstract

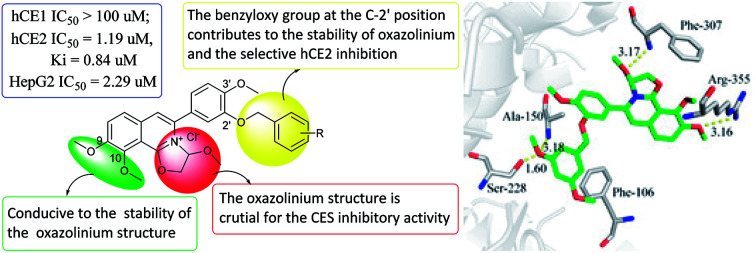

## Introduction

Mammalian carboxylesterases (CEs), important members of the serine hydrolase superfamily widely distributed in the lumen of endoplasmic reticulum in various tissues, are responsible for the hydrolysis of a wide range of endogenous and xenobiotic substrates containing ester, amides, thioesters and carbamates.^1–3^ In human body, hCE1 and hCE2 are the main carboxylesterases, both of which play crucial roles in endo- and xenobiotic metabolism. As one of the most abundant esterases distributed in human small intestine and colon, hCE2 participates in hydrolysis of the ester-bearing drugs (such as irinotecan, prasugrel, capecitabine, flutamide) and thereby affects the efficacy of these drugs.^4–7^ For instance, CPT-11 (irinotecan), an anticancer prodrug, exhibits strong anti-colorectal cancer activity by releasing the effective substance SN-38. However, excessive accumulation of SN-38 in the intestinal mucosa leads to delayed-onset diarrhoea even death.^8–10^ To improve the potential clinical risk of these drugs, some highly specific hCE2 inhibitors have been used in clinical to reduce the local exposure of SN-38 in the intestinal mucosa, thereby ameliorating the intestinal toxicity of CPT-11.^11,12^ Over the past decade, a wide variety of hCE2 inhibitors have been reported, including the natural triterpenoids,^13,14^ flavonoids,^13–15^ 1,2-diones^16,17^ and *etc.* Although many compounds with strong hCE2 inhibitory activities have already been developed, the potent and specific inhibitors targeting intracellular hCE2 are still rarely reported.

**Fig. 1:**
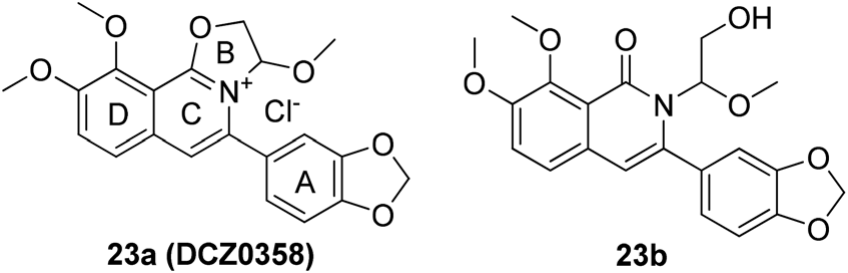
Structures of 23a (DCZ0358) and 23b.

**Fig. 2:**
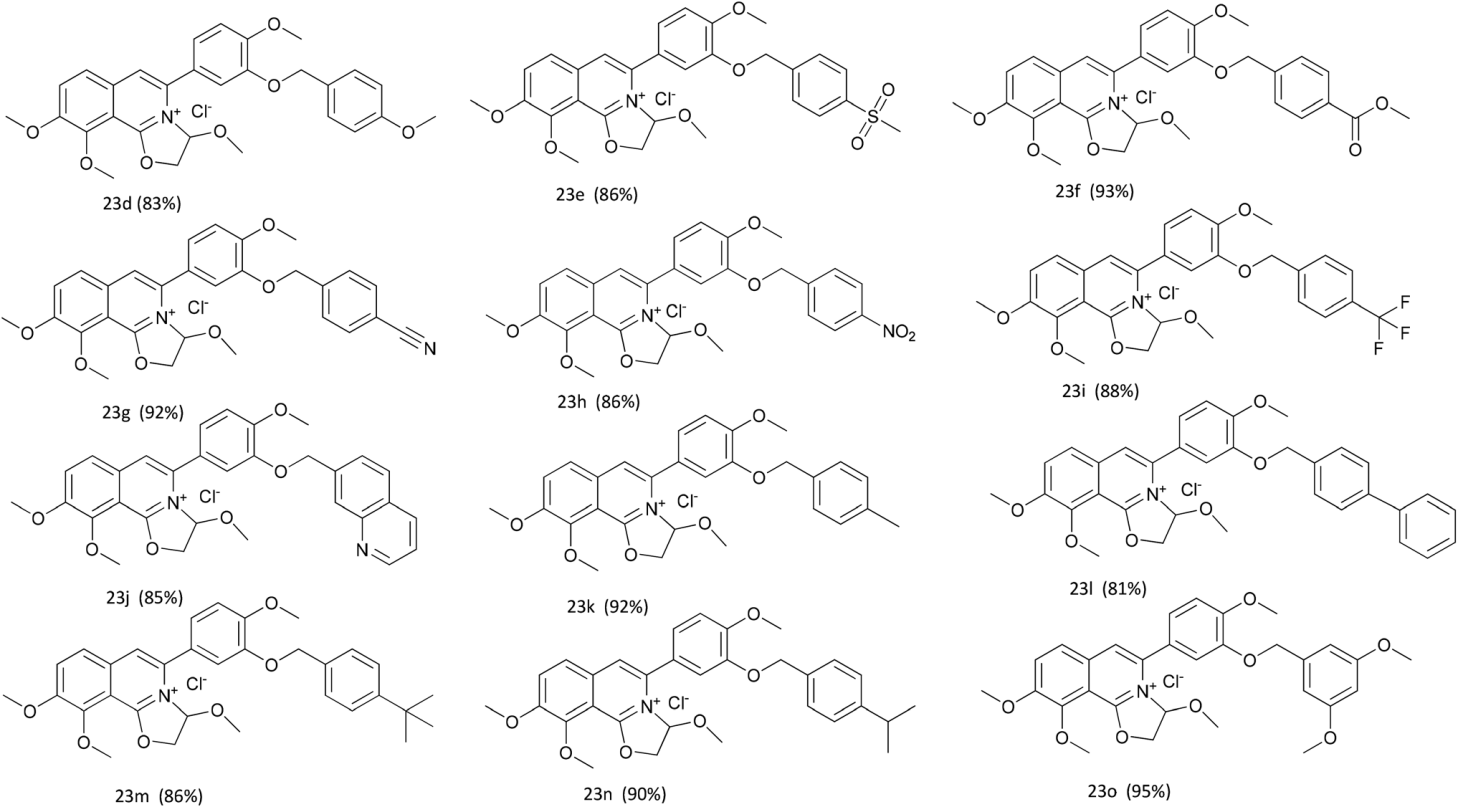
The structures of compounds 23d–o.

DCZ0358 (Fig. 1) is a novel dihydrooxazolo[2,3-*a*]isoquinolinium discovered in the synthesis of berberine analogues.^18–20^ Preliminary screening indicated that DCZ0358 could effectively inhibit the catalytic activity of both hCE1 (IC_50_ = 4.04 μM) and hCE2 (IC_50_ = 16.03 μM), while its hydrolyzate 23b showed a significant reduction of the inhibitory activity (hCE1 IC_50_ = 36.80 μM; hCE2 IC_50_ = 41.75 μM), which demonstrated that the oxazolinium moiety of DCZ0358 is essential for the CE_S_ inhibitory activity (Fig. 1). In the synthesis of derivatives of DCZ0358, we have found that in addition to compound 23d (Fig. 2), other compounds with modification of the substituents on the A and D rings cause structural instability of the quaternary ammonium salt. Moreover, the bioactivity and selectivity of 23d were improved (for hCE2 IC_50_ = 6.889 μM with >14.52-fold selectivity over hCE1). These results encouraged us to make further investigation of the structure–inhibition relationships of these berberine analogues as CEs inhibitors.

**Scheme 1:**
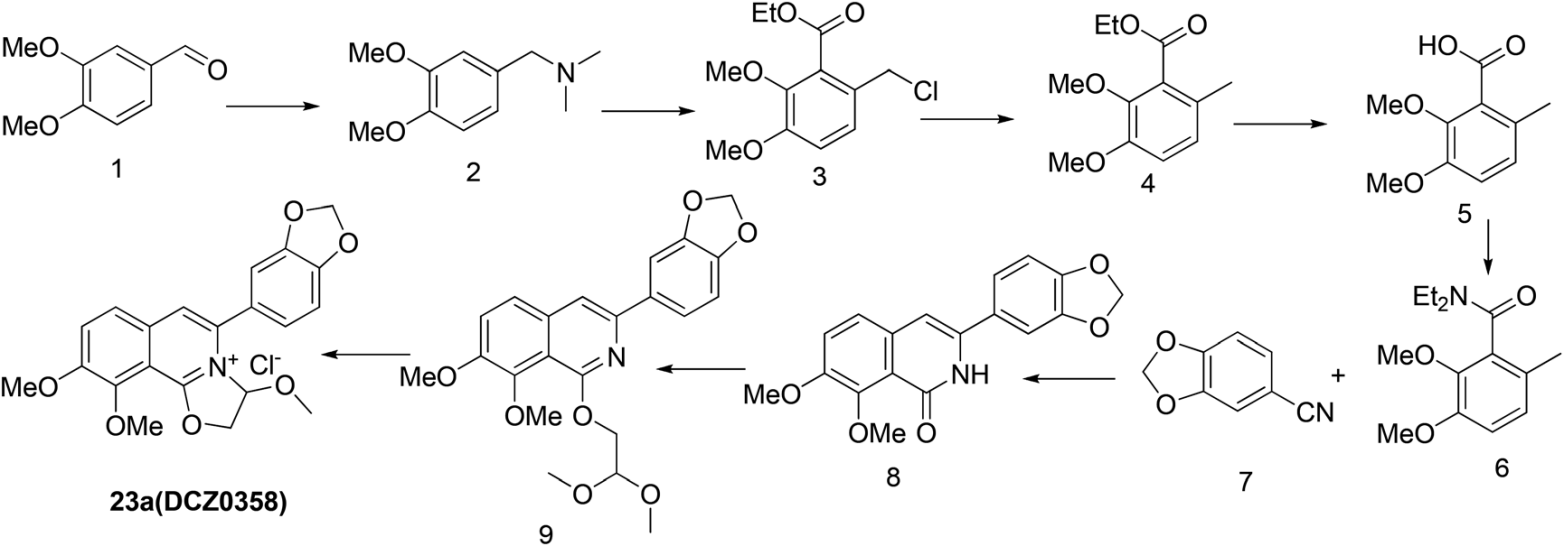
The original synthetic route.

**Scheme 2:**
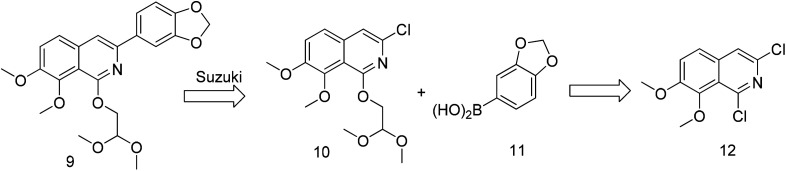
The retrosynthetic analysis.

The previously reported synthetic route of DCZ0358 is inconvenient to prepare more derivatives because of the harsh reaction conditions (Scheme 1).^21^ Therefore, we designed a new synthetic route using compound 12 as the key intermediate (Scheme 2). Among the obtained new analogues, 23o showed the highest selectivity and the best inhibitory activity (hCE1 IC_50_ > 100 μM; hCE2 IC_50_ = 1.192 μM, *K*_i_ = 0.84 μM). It was also found that 23o could inhibit hCE2 activity in living HepG2 cells with the IC_50_ value of 2.29 μM, suggesting that the compound has remarkable cell-membrane permeability and is capable for inhibiting intracellular hCE2. Further molecular docking results showed that the methoxyl group at the benzyloxy ring of 23o could tightly bind to the catalytic amino acid Ser-228 *via* H-bonding, which may account for the high selectivity of 23o on hCE2 over hCE1.

## Results and discussion

### Synthetic procedures

Previously, we reported the synthetic route of DCZ0358 (Scheme 1).^21^ However, the application of *n*-butyl lithium reagent and low temperature condition (−78 °C) restricted the synthesis of derivatives. Therefore, developing a feasible route is important for the further medicinal chemistry research. Based on the retrosynthetic analysis (Scheme 1), compound 9 could be synthesized *via* Suzuki coupling reaction from 10 and 11. Compound 10 could be smoothly prepared from the key intermediate 12.

**Scheme 3:**
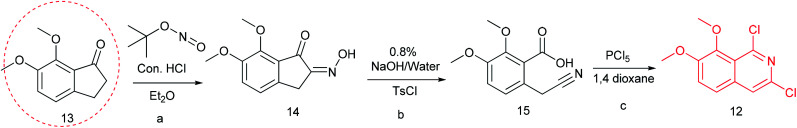
The synthesis of compound 12. Reagents and conditions: (a) *tert*-butyl nitrite, HCl (cat.), diethyl ether, r.t., 2 h; (b) NaOH (8%), 50 °C; TosCl, 80 °C, 3 h; (c) PCl_5_, 1,4-dioxane, 90 °C, 12 h.

Firstly, 6,7-dimethoxy-1-indanone 13 was adopted as the starting material (Scheme 3). After oximation of 13 with *tert*-butyl nitrite under acidic condition, ketoxime 14 was obtained through filtration.^22–24^ Subsequently, compound 14 was hydrolyzed by sodium hydroxide, and then dehydrated by *p*-toluenesulfonyl chloride to give benzonitrile 15.^24^ Finally, 15 was cyclized and chloridized by PCl_5_ to provide the dichloroisoquinoline 12.^24^ However, the starting material 13 is very expensive and difficult to be prepared, which promoted us searching for alternative synthetic route.

**Scheme 4:**
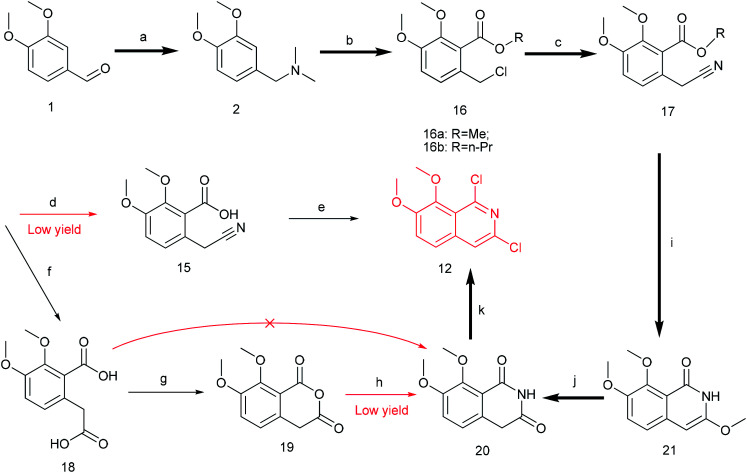
The synthesis of compound 12. Reagents and conditions: (a) DMF, HCOOH, 150 °C, 5 h; (b) *n*-BuLi (0 °C), ClCOOCH_3_/ClCOOC_3_H_7_ (−78 °C), dry THF, r.t 12 h; (c) TMSCN, TBAF, MeCN, 80 °C 12 h; (d) NaOH (2.9 M), 1,4-dioxane, 40 °C, 3 h; (e) PCl_5_, 1,4-dioxane, 90 °C, 12 h; (f) NaOH (10 M), 1,4-dioxane, 90 °C, 8 h; (g) AcCl, 50 °C, 2 h; (h) (NH_4_)_2_CO_3_, 280 °C, 2 h; (i) MeONa, dry MeOH, 80 °C 1 h; (j) MeOH, HCl (3 M), 100 °C 1 h; (k) PhPOCl_2_, 160 °C, 3 h.

Thus, we developed another route taking commercially available 3,4-dimethoxybenzaldehyde 1 as the starting material (Scheme 4). Compound 1 reacted with DMF and formic acid to afford tertiary amine 2 in 75% yield.^25^ Then we added chloroformate to the mixture of 2 and *n*-butyl lithium under −78 °C to produce 16 in 80% yield.^26,27^ Next, compound 16 was attracted by electrophilic reagent TMSCN to afford 17 (82% yield).^28^ The operation for the hydrolysis of the methyl ester compound 17 to the compound 15 is difficult to be control. Subsequently, both ester and cyano groups were hydrolyzed to carboxyl groups under strong alkaline condition to give 18 (76% yield). Compound 18 was easily dehydrated in the presence of acetyl chloride to obtain compound 19 in 78% yield.^29^ However, compound 20 was rather difficult to achieve from compound 18 or compound 19. After trying various amines, we found that only ammonium carbonate could react with 19.^30^ However, this reaction occurred at a high temperature (280 °C) and gave a very low yield (22% yield) of 20. Thus, compound 17 was directly reacted with sodium methoxide to afford compound 21 in 51% yield, followed by demethylation to produce dihydroisoquinoline-1,3-dione 20 with a high yield of 93%.^31^ In order to convert 20 to the key intermediate 12, we explored many reagents, such as PCl_5_, POCl_3_, SOCl_2_ and PhPOCl_2_, it turned out that PhPOCl_2_ behaved the best yield with 47%.^31^

**Scheme 5:**
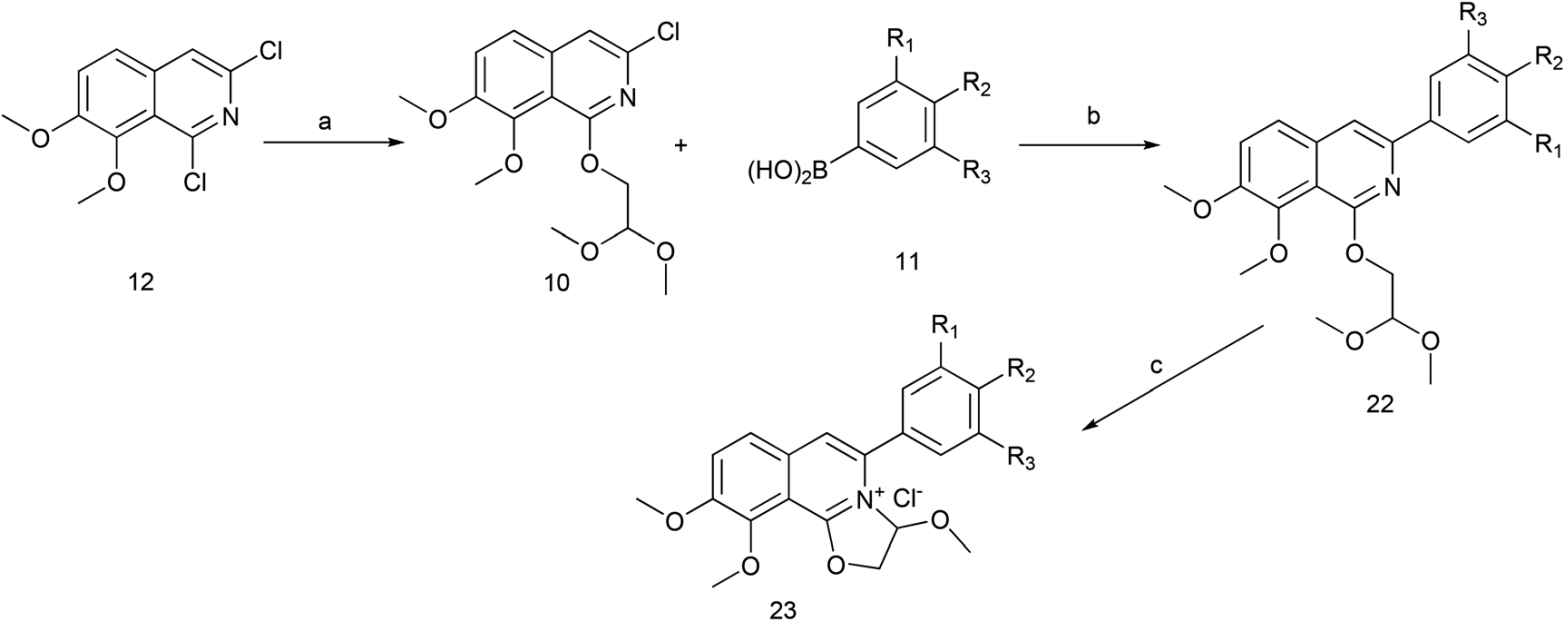
The synthesis of DCZ0358 analogues. Reagents and conditions: (a) NaH, glycolaldehyde dimethyl acetal, THF, r.t.12 h; (b) Pd_2_(dba)_3_, Xphos, K_3_PO_4_, 1,4-dioxane, 90 °C, 12 h; (c) acetone, HCl–Et_2_O (5 : 1), r.t. 1 h.

The key intermediate 12 reacted smoothly with hydroxyacetal under alkaline conditions to give compound 10 with high yield (98%),^32^ and then 10 reacted with various arylboronic acids containing a benzyloxy structure to produce 22 in yields ranging from 46% to 98%.^19^ Finally 22 were cyclized under acidic conditions to give a series of dihydrooxazolo[2,3-*a*]isoquinolinium analogues (Scheme 5, compounds 23d–23o in Fig. 2). The present synthetic route is convenient to scale up and benefits further pharmaceutical research.

### Biological activity assays

We designed and synthesized more than 30 derivatives of DCZ0358. However, the five-ring quaternary ammonium component of some derivatives was unstable to decompose easily into its hydrolyzate 23b. With 12 stable compounds in hand, we conducted experiments to assay inhibitory activities against both hCE1 and hCE2 using a panel of fluorescent probe substrates.^33–36^d-Luciferin methyl ester (DME) was used as a probe substrate, and nevadensin (a specific hCE1 inhibitor) was used as a positive inhibitor control for hCE1. Fluorescein diacetate (FD) was used as a specific probe substrate, and loperamide (LPA) was used as a positive inhibitor control of hCE2. The IC_50_ values of all derivatives were evaluated and listed in Table 1.

**Table 1:** The IC_50_ values of DCZ0358 and its derivatives on hCE1 and hCE2^a^

Compound	IC_50_ (μM) for hCE1	IC_50_ (μM) for hCE2	Selectivity IC_50_(hCE2)/IC_50_(hCE1)
23a	4.04 ± 0.40	16.03 ± 1.49	>0.25
23b	36.80 ± 7.70	41.75 ± 18.15	>0.88
23c	>100	6.89 ± 1.09	>14.52
23e	>100	11.46 ± 1.76	>8.72
23f	>100	5.73 ± 0.79	>17.46
23g	>100	3.33 ± 0.32	>30.07
23h	>100	3.32 ± 0.87	>30.14
23i	>100	2.43 ± 0.28	>41.22
23j	>100	3.77 ± 0.25	>26.51
23k	>100	5.58 ± 0.94	>17.94
23l	>100	2.64 ± 0.45	>37.89
23m	>100	2.33 ± 0.20	>42.81
23n	>100	1.66 ± 0.21	>60.31
23o^a^	>100	1.19 ± 0.10	>83.89
Nevadensin^b^	2.64 ± 0.22	—	—
LPA^c^	—	6.24 ± 0.93	—

a Inhibition potential of all compounds were investigated in living HepG2 cells.

b Nevadensin was used as a positive inhibitor of hCE1.

c LPA was used as a positive inhibitor of hCE2.

Table 1 showed that the inhibitory effects of these compounds against hCE2 were enhanced significantly when the methylenedioxy group on A ring was changed into benzyloxy group. The IC_50_ values of 23n (hCE2 IC_50_ 1.66 ± 0.21 μM) and 23o (hCE2 IC_50_ 1.19 ± 0.10 μM) were improved more than tenfold comparing to that of 23a (hCE2 IC_50_ 16.03 ± 1.49 μM). However, different types of the substituents on the A ring didn't have significant effects on the inhibitory activities, *e.g.*, 23k (hCE2 IC_50_ 5.58 ± 0.94 μM), 23l (hCE2 IC_50_ 2.64 ± 0.45 μM), 23m (hCE2 IC_50_ 2.33 ± 0.20 μM), 23n (hCE2 IC_50_ 1.66 ± 0.21 μM) and 23o (hCE2 IC_50_ 1.19 ± 0.10 μM) with electron-donating groups on the benzyloxy ring were similar to that of 23e (hCE2 IC_50_ 11.46 ± 1.76 μM), 23f (hCE2 IC_50_ 5.73 ± 0.79 μM) and 23h (hCE2 IC_50_ 3.32 ± 0.87 μM) with electron-withdrawing groups. In terms of the selectivity, it improved apparently according to the values of IC_50_ (hCE2)/IC_50_ (hCE1) shown in Table 1. For instance, the value of IC_50_ (hCE2)/IC_50_ (hCE1) of 23o was up to 83 while that of 23a was only 0.25. Thus, 23o have the best selectivity on hCES2 among all these newly synthesized compounds.

**Fig. 3:**
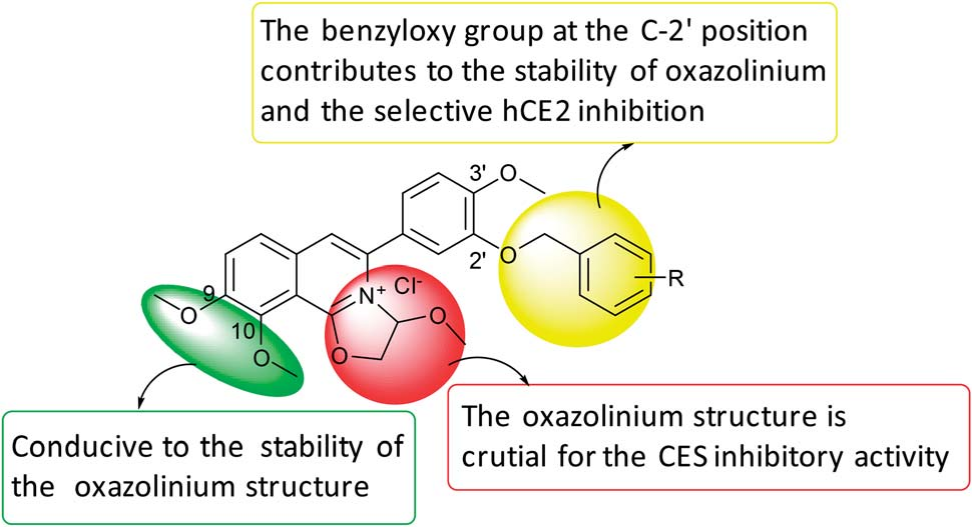
SAR summary of DCZ0358 analogues.

Collectively, the structure–activity relationships of these compounds were summarized as follows, (1) the oxazolinium moiety is crucial for the inhibitory activity against hCEs; (2) the benzyloxy group on the A ring mainly contributed to the selectivity of hCE2 over hCE1 (Fig. 3).

**Fig. 4:**
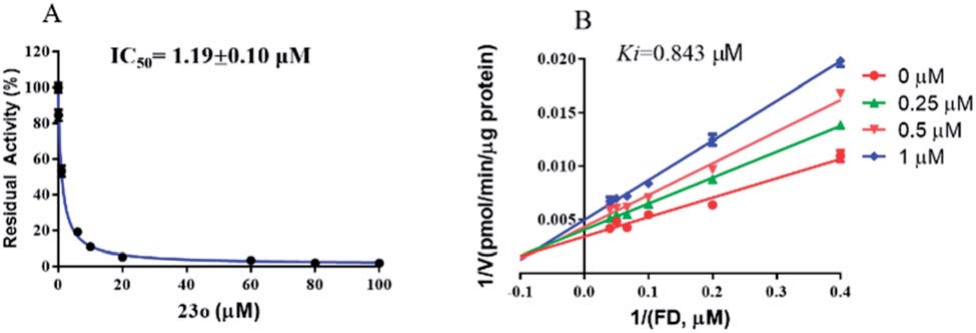
The dose-dependent inhibition curve of 23o (A) and the Lineweaver–Burk plots of 23o against hCE2-mediated FD hydrolysis (B).

**Fig. 5:**
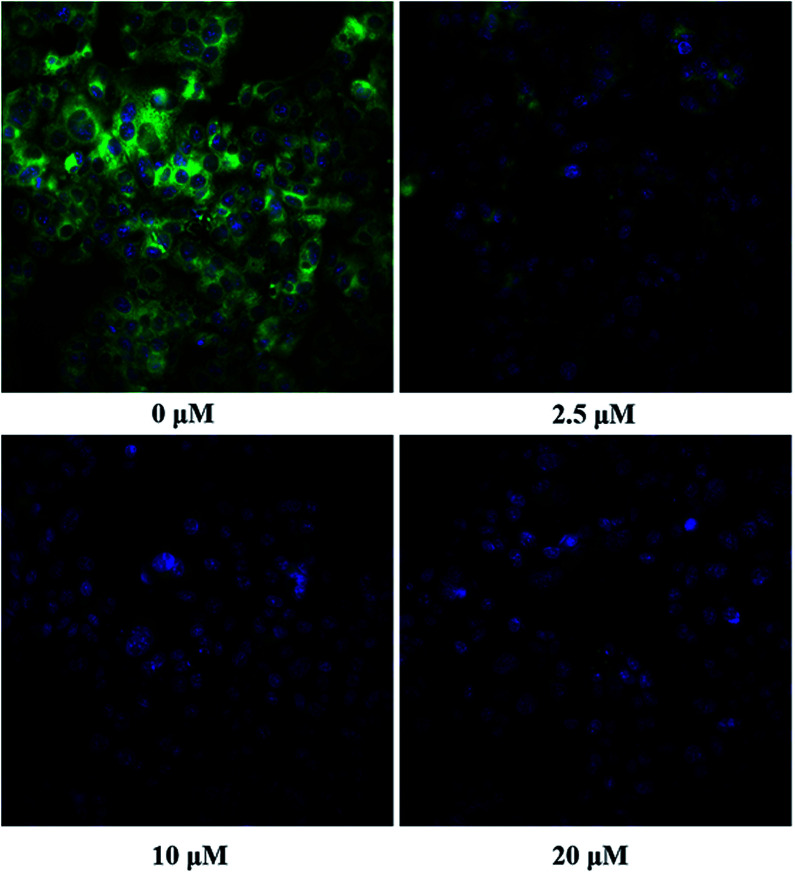
The images of HepG2 cells stained with NCEN (10 μM) and Hoechst 33342 (1 μM) at 37 °C for 50 min in the presence of 23o at various concentrations (0 μM, 2.5 μM, 10 μM and 20 μM). All data was shown as mean ± SD.

The inhibition kinetic of 23o against hCE2-mediated FD hydrolysis has been carefully investigated and the results showed that 23o functioned as a mixed inhibitor against hCE2-mediated FD hydrolysis, with the *K*_i_ value of 0.84 μM (Fig. 4B). Furthermore, in view of that hCE2 is an intracellular enzyme, the inhibition potential of 23o was also investigated. As shown in Fig. 5, 23o could strongly inhibit intracellular hCE2-mediated NCEN hydrolysis and reduce the fluorescence intensity in the green channel (for the hydrolytic metabolite of NCEN) in living HepG2 cells *via* a dose-dependent manner. Meanwhile, the IC_50_ value of 23o against intracellular hCE2 was also evaluated as 2.29 μM (Fig. S2B†).

### Molecular docking

**Fig. 6:**
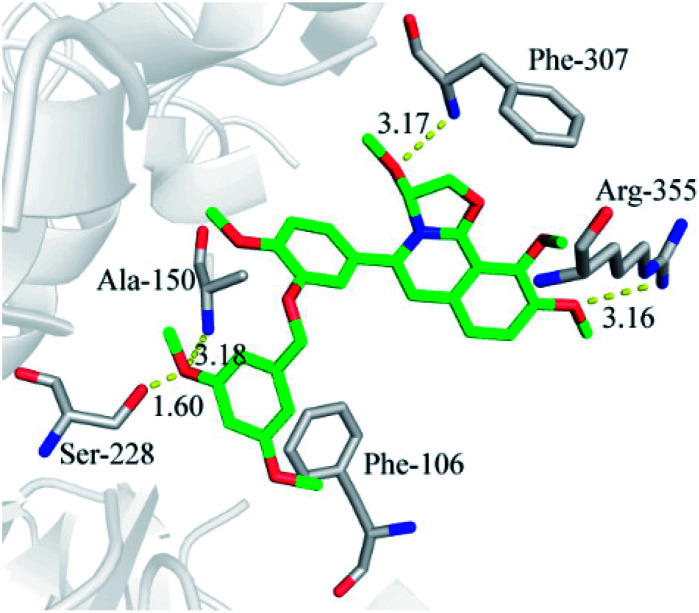
Molecular docking indicates the interactions between compound 23o and hCE2.

In order to investigate the interaction mechanism of 23o with hCE2, molecular docking of 23o to the active site of hCE2 was performed. As shown in Fig. 6, there are hydrogen bond between the methoxyl of ring D with Arg-355 (3.16 Å), and a T-type π–π interaction between the ring D with the Arg-355, as well as, hydrogen bond between the oxygen atom of ring B with Phe-307 (3.17 Å) in the entrance of the active cavity of hCE2. These interactions facilitate the entry of 23o into the active cavity of hCE2. However, the hydrolysate of 23o cannot enter the active cavity of hCE2, due to its small inlet. In addition, the methoxyl group at the benzyloxy end of 23o could tightly bind to the catalytic amino acid Ser-228 (1.6 Å) *via* strong H-bonding, as well as, with Ala-150 (3.18 Å), and there are strong hydrophobic interactions between the benzyloxy group of 23o with the key residues in the active cavity of hCE2. These interactions may account for the high selectivity of 23o on hCE2. The strong H-bond interaction between 23o and Ser-228 indicates that 23o may obstruct hCE2-mediated hydrolysis, possibly because Ser-228 is an important residue involved in substrate recognition and catalysis of hCE2. These findings agreed well with the experimental data where 23o exhibited much more potent inhibitory effect on hCE2 but a relatively weaker one on hCE1.

## Conclusions

A new compound 23o with a novel skeleton of dihydrooxazolo[2,3-*a*]isoquinolinium was discovered with good inhibitory activity on hCE2 (IC_50_ = 1.19 μM, *K*_i_ = 0.84 μM) and high selectivity over hCE1 (IC_50_ > 100 μM). The SAR (structure–activity relationship) analysis and molecular docking results revealed that the novel oxazolinium moiety is essential for hCE2 inhibitory activity, while the benzyloxy moiety contributes to the selectivity of hCE2 over hCE1. Furthermore, 23o could strongly inhibit intracellular hCE2 in living HepG2 cells, with the IC_50_ value of 2.29 μM. These findings are important for further research and development of hCE2 inhibitors with high specificity and efficacy.

## Experimental

### Chemical synthesis

#### Materials

All starting materials were obtained from commercial suppliers and used without further purification. The ^1^H and ^13^C NMR spectra were taken on Bruker Avance-600 or 500 or 400, Varian MERCURY Plus-400 or 300 NMR spectrometer operating at 400 MHz or 300 MHz for ^1^H NMR, 125 MHz or 100 MHz for ^13^C NMR, using TMS as internal standard and CDCl_3_ or methanol-*d*_4_ or DMSO-*d*_6_ as solvent.^13^C NMR spectra were recorded with complete proton decoupling. The ESI-MS or EI-MS was recorded on Finnigan LCQ/DECA or Thermo-DFS, respectively. The HRMS were obtained from Micromass Ultra Q-TOF (ESI) or Thermo-DFS (EI) spectrometer. Flash column chromatography was carried out using silica gel (200–400 mesh). Thin layer chromatography (TLC) was used silica gel F254 fluorescent treated silica that were visualised under UV light (254 nm).

#### Synthetic procedure

Compounds DCZ0358 and 23b have been reported in our previous work.^18,20^ Synthesis of 3,9,10-trimethoxy-5-(4-methoxy-3-((4-methoxybenzyl)oxy)phenyl)-2,3-dihydrooxazolo[2,3-*a*]isoquinolin-4-ium (23d). To a solution of 22d (56 mg, 0.1 mmol) in acetone (5 mL) was added hydrochloric acid (1 mL, 2.0 M in diethyl ether), and then the mixture was stirred for 2 h at room temperature. The solution was evaporated *in vacuo* to obtain the titled compound 23d as yellow solid (46 mg, 83%). ^1^H NMR (500 MHz, DMSO-*d*_6_) *δ* 8.15 (s, 1H), 7.99 (s, 1H), 7.81 (s, 1H), 7.34 (s, 2H), 7.13 (s, 3H), 6.91 (s, 2H), 6.62 (s, 1H), 5.30 (s, 1H), 5.20 (s, 1H), 4.69 (s, 2H), 4.02 (s, 3H), 3.95 (s, 3H), 3.85 (s, 3H), 3.73 (s, 3H), 2.95 (s, 3H). ^13^C NMR (125 MHz, DMSO-*d*_6_) *δ* 161.2, 159.7, 152.9, 149.9, 147.3, 146.2, 136.8, 134.5, 130.9, 130.1, 126.1, 124.9, 124.5, 120.9, 118.8, 116.5, 114.5, 112.8, 110.6, 90.9, 76.5, 62.3, 57.5, 56.3, 55.8, 55.7, 46.8. HRMS (EI) calcd for C_30_H_17_O_7_N 503.1000 [M]^+^, found 503.0990.

##### 3,9,10-Trimethoxy-5-(4-methoxy-3-((4-(methylsulfonyl)benzyl)oxy)phenyl)-2,3-dihydrooxazolo[2,3-*a*]isoquinolin-4-ium (23e)

Compound 23e was prepared from compound 22e (58 mg, 0.1 mmol) as a yellow solid (48 mg, 86%). ^1^H NMR (400 MHz, DMSO-*d*_6_) *δ* 8.18 (d, *J* = 7.6 Hz, 1H), 7.97 (d, *J* = 7.2 Hz, 3H), 7.87 (s, 1H), 7.73 (s, 2H), 7.44 (s, 1H), 7.32 (s, 1H), 7.26 (s, 1H), 6.62 (s, 1H), 5.33 (m, 4H), 4.13–3.82 (m, 9H), 3.22 (s, 3H), 2.85 (s, 3H). ^13^C NMR (125 MHz, DMSO-*d*_6_) *δ* 161.1, 153.0, 151.3, 147.9, 146.2, 143.2, 140.8, 136.4, 134.4, 128.7, 127.7, 126.2, 124.9, 124.3, 123.3, 119.1, 114.9, 112.9, 110.5, 91.0, 76.4, 69.8, 62.3, 57.5, 56.4, 55.8, 44.0. HRMS (EI) calcd for C_29_H_29_O_8_NS 551.1608 [M]^+^, found 551.1607.

##### 3,9,10-Trimethoxy-5-(4-methoxy-3-((4-(methoxycarbonyl)benzyl)oxy)phenyl)-2,3-dihydrooxazolo[2,3-*a*]isoquinolin-4-ium (23f)

Compound 23f was prepared from compound 22f (56 mg, 0.1 mmol) as a yellow solid (50 mg, 93%). ^1^H NMR (600 MHz, CDCl_3_) *δ* 8.17 (d, *J* = 8.9 Hz, 2H), 8.01 (s, 2H), 7.99 (s, 1H), 7.86 (s, 1H), 7.61 (d, *J* = 7.8 Hz, 2H), 7.42 (s, 1H), 7.32 (m, 1H), 7.25 (d, *J* = 8.4 Hz, 1H), 6.61 (d, *J* = 6.0 Hz, 1H), 5.33 (m, 2H), 5.27 (m, 1H), 5.22 (m, 1H), 4.04 (s, 3H), 3.97 (s, 3H), 3.89 (s, 3H), 3.85 (s, 3H), 2.86 (s, 3H). ^13^C NMR (125 MHz, DMSO-*d*_6_) *δ* 166.5, 161.1, 153.0, 151.3, 148.0, 146.2, 142.7, 136.4, 134.4, 129.8, 129.6, 128.1, 126.1, 124.9, 124.3, 123.2, 119.1, 114.9, 112.9, 110.6, 91.0, 76.4, 70.0, 62.3, 57.4, 56.4, 55.8, 52.7. HRMS (EI) calcd for C_30_H_29_O_8_N 531.1888 [M]^+^, found 531.1882.

##### 5-(3-((4-Cyanobenzyl)oxy)-4-methoxyphenyl)-3,9,10-trimethoxy-2,3-dihydrooxazolo[2,3-*a*]isoquinolin-4-ium (23g)

Compound 23g was prepared from compound 22g (53 mg, 0.1 mmol) as a yellow solid (46 mg, 92%). ^1^H NMR (600 MHz, DMSO-*d*_6_) *δ* 8.17 (d, *J* = 9.0 Hz, 1H), 8.01 (d, *J* = 9.0 Hz, 1H), 7.90 (d, *J* = 4.2 Hz, 2H), 7.86 (s, 1H), 7.66 (d, *J* = 7.8 Hz, 2H), 7.43 (d, *J* = 1.8 Hz, 1H), 7.33 (dd, *J* = 8.4, 1.8 Hz, 1H), 7.26 (d, *J* = 7.8 Hz, 1H), 6.64 (d, *J* = 6.0 Hz, 1H), 5.34 (m, 2H), 5.29 (m, 1H), 5.24 (m, 1H), 4.05 (s, 3H), 3.98 (s, 3H), 3.90 (s, 3H), 2.87 (s, 3H). ^13^C NMR (125 MHz, DMSO-*d*_6_) *δ* 161.1, 153.0, 151.3, 147.8, 146.2, 143.0, 136.3, 134.4, 133.0, 128.6, 126.1, 124.9, 124.3, 123.3, 119.2, 119.1, 114.8, 112.9, 111.1, 110.6, 91.0, 76.4, 69.7, 62.3, 57.4, 56.4, 55.8. HRMS (EI) calcd for C_29_H_26_O_6_N_2_ 498.1785 [M]^+^, found 498.1785.

##### 3,9,10-Trimethoxy-5-(4-methoxy-3-((4-nitrobenzyl)oxy)phenyl)-2,3-dihydrooxazolo[2,3-*a*]isoquinolin-4-ium (23h)

Compound 23h was prepared from compound 22h (55 mg, 0.1 mmol) as a yellow solid (45 mg, 86%). ^1^H NMR (400 MHz, DMSO-*d*_6_) *δ* 8.30 (d, *J* = 8.4 Hz, 2H), 8.19 (d, *J* = 9.2 Hz, 1H), 8.01 (d, *J* = 9.2 Hz, 1H), 7.87 (s, 1H), 7.74 (d, *J* = 8.4 Hz, 2H), 7.43 (s, 1H), 7.34 (d, *J* = 8.4 Hz, 1H), 7.28 (d, *J* = 8.4 Hz, 1H), 6.66 (d, *J* = 5.2 Hz, 1H), 5.44–5.35 (m, 2H), 5.34 (s, 1H), 5.23 (d, *J* = 6.4 Hz, 1H), 4.05 (s, 3H), 3.98 (s, 3H), 3.91 (s, 3H), 2.89 (s, 3H). ^13^C NMR (125 MHz, DMSO-*d*_6_) *δ* 160.6, 152.5, 150.8, 147.3, 147.1, 145.7, 144.6, 135.8, 133.9, 128.3, 125.7, 124.4, 123.8, 123.6, 122.9, 118.6, 114.4, 112.4, 110.1, 90.5, 75.9, 69.1, 61.8, 56.9, 55.9, 55.3. HRMS (EI) calcd for C_28_H_26_O_8_N_2_ 518.1684 [M]^+^, found 518.1688.

##### 3,9,10-Trimethoxy-5-(4-methoxy-3-((4-(trifluoromethyl)benzyl)oxy)phenyl)-2,3-dihydrooxazolo[2,3-a]isoquinolin-4-ium (23i)

Compound 23i was prepared from compound 22i (57 mg, 0.1 mmol) as a yellow solid (48 mg, 88%). ^1^H NMR (500 MHz, DMSO-*d*_6_) *δ* 8.20 (d, *J* = 7.2 Hz, 1H), 8.03 (d, *J* = 6.8 Hz, 1H), 7.89 (s, 1H), 7.82 (d, *J* = 6.0 Hz, 2H), 7.72 (d, *J* = 6.4 Hz, 2H), 7.46 (s, 1H), 7.35 (d, *J* = 6.4 Hz, 1H), 7.28 (d, *J* = 6.4 Hz, 1H), 6.64 (s, 1H), 5.36 (m, 3H), 5.27 (s, 1H), 4.07 (s, 3H), 4.01 (s, 3H), 3.93 (s, 3H), 2.88 (s, 3H). ^13^C NMR (125 MHz, DMSO-*d*_6_) *δ* 160.6, 152.5, 150.8, 147.4, 145.7, 141.6, 135.9, 133.9, 128.1, 125.7, 125.4, 125.4, 124.4, 123.8, 122.7, 119.4, 118.6, 114.3, 112.4, 110.1, 90.5, 75.9, 69.3, 61.8, 56.9, 55.9, 55.3. HRMS (EI) calcd for C_29_H_26_O_6_NF_3_ 541.1707 [M]^+^, found 541.1710.

##### 3,9,10-Trimethoxy-5-(4-methoxy-3-(quinolin-7-ylmethoxy)phenyl)-2,3-dihydrooxazolo[2,3-*a*]isoquinolin-4-ium (23j)

Compound 23j was prepared from compound 22j (58 mg, 0.1 mmol) as a yellow solid (45 mg, 85%). ^1^H NMR (400 MHz, DMSO-*d*_6_) *δ* 8.99 (s, 1H), 8.57 (d, *J* = 7.2 Hz, 1H), 8.18 (d, *J* = 8.0 Hz, 1H), 8.07 (d, *J* = 7.6 Hz, 1H), 8.00 (s, 2H), 7.89 (s, 1H), 7.73 (s, 1H), 7.70 (s, 1H), 7.57 (s, 1H), 7.32 (d, *J* = 5.6 Hz, 1H), 7.26 (d, *J* = 7.2 Hz, 1H), 6.74 (s, 1H), 5.91 (m, 1H), 5.82 (m, 1H), 5.34 (m, 1H), 5.23 (s, 1H), 4.04 (s, 3H), 3.96 (s, 3H), 3.89 (s, 3H), 2.93 (s, 3H). ^13^C NMR (125 MHz, DMSO-*d*_6_) *δ* 161.1, 153.0, 151.2, 150.0, 148.5, 146.2, 136.5, 134.5, 134.0, 129.8, 129.2, 128.7, 128.4, 127.3, 126.1, 125.0, 124.4, 122.9, 122.3, 119.1, 115.7, 114.3, 112.8, 110.6, 91.0, 76.5, 67.0, 62.3, 57.5, 56.4, 55.8. HRMS (EI) calcd for C_31_H_28_O_6_N_2_ 524.1942 [M]^+^, found 524.1951.

##### 3,9,10-Trimethoxy-5-(4-methoxy-3-((4-methylbenzyl)oxy)phenyl)-2,3-dihydrooxazolo[2,3-*a*]isoquinolin-4-ium (23k)

Compound 23k was prepared from compound 22k (52 mg, 0.1 mmol) as a yellow solid (45 mg, 92%). ^1^H NMR (400 MHz, DMSO-*d*_6_) *δ* 8.16 (s, 1H), 7.99 (s, 1H), 7.85 (s, 1H), 7.39 (s, 1H), 7.33 (s, 2H), 7.26 (s, 1H), 7.20 (s, 3H), 6.54 (s, 1H), 5.31 (s, 1H), 5.13 (s, 3H), 4.02 (s, 3H), 3.95 (s, 3H), 3.85 (s, 3H), 2.82 (s, 3H), 2.28 (s, 3H). ^13^C NMR (125 MHz, DMSO-*d*_6_) *δ* 160.6, 152.4, 150.8, 147.6, 145.7, 137.3, 136.0, 133.9, 133.5, 129.0, 127.9, 125.7, 124.5, 123.7, 122.4, 118.5, 114.1, 112.3, 110.0, 90.5, 76.0, 70.0, 61.9, 57.1, 55.9, 55.4, 20.8. HRMS (EI) calcd for C_29_H_29_O_6_N 487.1989 [M]^+^, found 487.1989.

##### 5-(3-([1,1′-Biphenyl]-4-ylmethoxy)-4-methoxyphenyl)-3,9,10-trimethoxy-2,3-dihydrooxazolo[2,3-*a*]isoquinolin-4-ium (23l)

Compound 23l was prepared from compound 22l (58 mg, 0.1 mmol) as a yellow solid (45 mg, 81%). ^1^H NMR (500 MHz, DMSO-*d*_6_) *δ* 8.20 (d, *J* = 6.8 Hz, 1H), 8.03 (d, *J* = 6.8 Hz, 1H), 7.90 (s, 1H), 7.74 (d, *J* = 6.0 Hz, 2H), 7.70 (d, *J* = 6.0 Hz, 2H), 7.58 (d, *J* = 6.0 Hz, 2H), 7.53–7.47 (m, 3H), 7.40 (t, *J* = 6.0 Hz, 1H), 7.33 (d, *J* = 6.4 Hz, 1H), 7.27 (d, *J* = 6.4 Hz, 1H), 6.63 (s, 1H), 5.34 (m, 1H), 5.27 (m, 3H), 4.07 (s, 3H), 4.00 (s, 3H), 3.93 (s, 3H), 2.88 (s, 3H). ^13^C NMR (125 MHz, DMSO-*d*_6_) *δ* 160.6, 152.5, 150.8, 147.7, 145.7, 139.9, 139.7, 136.0, 135.8, 133.9, 129.0, 128.4, 127.6, 126.8, 126.6, 125.7, 124.4, 123.8, 122.4, 118.6, 114.1, 112.3, 110.0, 90.5, 76.0, 69.8, 61.8, 57.0, 55.8, 55.4. HRMS (EI) calcd for C_34_H_31_O_6_N 549.2146 [M]^+^, found 549.2146.

##### 5-(3-((4-(*tert*-Butyl)benzyl)oxy)-4-methoxyphenyl)-3,9,10-trimethoxy-2,3-dihydrooxazolo[2,3-*a*]isoquinolin-4-ium (23m)

Compound 23m was prepared from compound 22m (56 mg, 0.1 mmol) as a yellow solid (46 mg, 86%). ^1^H NMR (500 MHz, DMSO-*d*_6_) *δ* 8.18 (d, *J* = 7.2 Hz, 1H), 8.02 (d, *J* = 6.8 Hz, 1H), 7.88 (s, 1H), 7.44 (d, *J* = 5.6 Hz, 3H), 7.40 (d, *J* = 6.4 Hz, 2H), 7.30 (d, *J* = 6.8 Hz, 1H), 7.23 (d, *J* = 6.8 Hz, 1H), 6.56 (s, 1H), 5.32 (m, 1H), 5.26 (m, 1H), 5.17 (m, 2H), 4.05 (s, 3H), 3.99 (s, 3H), 3.88 (s, 3H), 2.82 (s, 3H), 1.29 (s, 9H). ^13^C NMR (125 MHz, DMSO-*d*_6_) *δ* 161.1, 153.0, 151.3, 151.1, 148.2, 146.2, 136.5, 134.5, 134.1, 128.3, 126.2, 125.7, 124.9, 124.3, 122.8, 119.0, 114.4, 112.7, 110.5, 91.0, 76.5, 70.3, 62.3, 57.5, 56.3, 55.9, 34.8, 31.6. HRMS (EI) calcd for C_32_H_35_O_6_N 529.2459 [M]^+^, found 529.2459.

##### 5-(3-((4-Isopropylbenzyl)oxy)-4-methoxyphenyl)-3,9,10-trimethoxy-2,3-dihydrooxazolo[2,3-*a*]isoquinolin-4-ium (23n)

Compound 23n was prepared from compound 22n (55 mg, 0.1 mmol) as a yellow solid (50 mg, 90%).^1^H NMR (600 MHz, DMSO-*d*_6_) *δ* 8.18 (d, *J* = 6.0 Hz, 1H), 8.01 (d, *J* = 6.0 Hz, 1H), 7.87 (s, 1H), 7.43 (d, *J* = 1.2 Hz, 1H), 7.39 (d, *J* = 5.2 Hz, 2H), 7.31–7.27 (m, 3H), 7.22 (d, *J* = 5.6 Hz, 1H), 6.55 (d, *J* = 4.0 Hz, 1H), 5.31 (m, 1H), 5.24 (m, 1H), 5.18 (m, 1H), 5.13 (m, 1H), 4.05 (s, 3H), 3.98 (s, 3H), 3.87 (s, 3H), 2.94–2.85 (m, 1H), 2.82 (s, 3H), 1.20 (s, 3H), 1.19 (s, 3H). ^13^C NMR (125 MHz, DMSO-*d*_6_) *δ* 161.1, 153.0, 151.3, 148.8, 148.2, 146.2, 136.5, 134.5, 134.4, 128.5, 126.9, 126.1, 124.9, 124.3, 122.8, 119.0, 114.4, 112.7, 110.5, 91.0, 76.4, 70.4, 62.3, 57.4, 56.3, 55.9, 33.7, 24.3. HRMS (EI) calcd for C_31_H_33_O_6_N 515.2302 [M]^+^, found 515.2295.

##### 5-(3-((3,5-Dimethoxybenzyl)oxy)-4-methoxyphenyl)-3,9,10-trimethoxy-2,3-dihydrooxazolo[2,3-*a*]isoquinolin-4-ium (23o)

Compound 23o was prepared from compound 22o (56 mg, 0.1 mmol) as a yellow solid (51 mg, 95%). ^1^H NMR (500 MHz, DMSO-*d*_6_) *δ* 8.18 (d, *J* = 8.5 Hz, 1H), 8.00 (d, *J* = 9.0 Hz, 1H), 7.87 (m, 1H), 7.39 (s, 1H), 7.32–7.26 (m, 1H), 7.24 (d, *J* = 8.5 Hz, 1H), 6.62 (s, 2H), 6.52 (s, 1H), 6.47 (s, 1H), 5.33 (m, 1H), 5.26–5.17 (m, 1H), 5.15 (s, 2H), 4.05 (s, 3H), 3.98 (s, 3H), 3.90 (s, 3H), 3.74 (s, 6H), 2.84 (s, 3H). ^13^C NMR (125 MHz, DMSO-*d*_6_) *δ* 161.1, 161.1, 153.0, 151.4, 148.0, 146.2, 139.5, 136.5, 134.4, 126.2, 124.9, 124.2, 123.0, 119.0, 114.7, 112.9, 110.5, 106.1, 99.8, 91.0, 76.4, 70.4, 62.3, 57.5, 56.4, 55.9, 55.7. HRMS (EI) calcd for C_30_H_31_O_8_N 533.2044 [M]^+^, found 533.2031.

### Biology

#### Materials

Fluorescein diacetate (FD) and loperamide (LPA) were purchased from TCI (Tokyo Japan), Luciferin Detection Reagent (LDR) was purchased from Promega Corporation (USA). *N*-(2-Butyl-1,3-dioxo-2,3-dihydro-1*H*-phenalen-6-yl)-2-chloroacetamide (NCEN) and d-luciferin methyl ester (DME) were synthesized by authors according to the previously reported synthetic scheme.^37,38^ Nevadensin was purchased from Chengdu Preferred Biotech Co., Ltd. (Chengdu, China). Pooled human liver microsomes (HLMs, from 50 donors, lot no. X008067) were obtained from Bioreclamation IVT (Baltimore, MD, USA) and stored at −80 °C until use. DMSO was purchased from fisher. Phosphate buffer was prepared using Millipore water and then stored at 4 °C until use. All tested compounds were solved by DMSO and stored at 4 °C until use. LC grade acetonitrile and DMSO (Tedia, USA) were used throughout.

#### hCE1 inhibition assay

DME was used as a probe substrate for evaluating the inhibitory effects of all DCZ0358 derivatives on hCE1, while nevadensin (a specific hCE1 inhibitor) was used as a positive control.^39^ Briefly, 100 μL incubation mixture containing 91 μL PBS (pH 6.8), 2 μL inhibitor at different concentrations and 5 μL HLM (1 μg mL^−1^, final concentration), were pre-incubated at 37 °C for 10 min. Subsequently, 2 μL DME (3 μM final concentration, close to the *K*_m_ value of DME in HLM) was added to initiate the reaction. After incubating for 10 min at 37 °C in a shaking bath, the reaction was stopped by the addition of LDR (100 μL). The microplate reader (SpectraMax® iD3, Molecular Devices, Austria) was used for luminescence measurements. The gain value of luminescence detection was set at 140 volts, and the integration time was set at 1 s. The chemical structure of DME and its hydrolytic metabolite (d-luciferin), as well as the detection conditions for d-luciferin are depicted in Table S1.† The negative control incubation (DMSO only) was carried out under the same conditions. The residual activity was calculated using the following formula, the residual activity (%) = (the florescence intensity in the presence of inhibitor)/the florescence intensity in negative control × 100%. The residual activities are show in Fig. S1.†

#### hCE2 inhibition assay

The inhibitory effects of all DCZ0358 derivatives on hCE2 were investigated using fluorescein diacetate (FD) as a specific probe substrate,^40^ while LPA was used as a positive inhibitor of hCE2 in this study.^41^ In brief, 200 μL incubation mixture containing 0.1 M PBS (PH = 7.4), human liver microsomes (2 μg mL^−1^, final concentration) and each inhibitor. After 10 min pre-incubation at 37 °C, the reaction was initiated by adding FD (5 μM, final concentration, close to the *K*_m_ value of FD in HLM). After incubating for 30 min at 37 °C in a shaking bath, the reaction was stopped by the addition of acetonitrile (200 μL). The chemical structure of FD and its hydrolytic metabolite (fluorescein), as well as the detection conditions for fluorescein are depicted in Table S1.† The negative control incubation (DMSO only) was also carried out under the same conditions.^42^ The residual activity was calculated using the formula mentioned above in hCE1 inhibition assay. The residual activities are shown in Fig. S1.†

#### Cell culture and fluorescence imaging analyses

In view of that hCE2 was an intracellular enzyme, the inhibition potential of 23o was investigated in living HepG2 cells. The HepG2 cells were cultured in Modified Eagle's Medium (MEM) with 5% CO_2_ and 0.1% antibiotic–antimycoticmix antibiotic at 37 °C, supplemented with 10% fetal bovine serum (FBS) and used NCEN as substrate probe to assay the 23o inhibition potential toward hCE2. NCEN,^43^ another specific optical probe substrate for hCE2, the structure and hydrolytic site were shown in Fig. S2(A).†

For fluorescence imaging, HepG2 cells were seeded in 96-well plates (8000 cells per well) with complete medium and then incubated for 24 hours. Afterwards, the cells were washed twice with FBS-free culture medium and then preincubated in the medium containing 23o (prepared in FBS-free at various concentrations) for 30 min with 5% CO_2_ at 37 °C. HepG2 cells were then co-incubated with NCEN (final concentration, 10 μM) for another 50 min to assess the intracellular hCE2 function, respectively. The living cells were imaged and analyzed using an ImageXpress® Micro Confocal High-Content Imaging system (Molecular Devices, Austria).

## Conflicts of interest

There are no conflicts to declare.

## Supplementary Material

RA-009-C9RA07457K-s001

RA-009-C9RA07457K-s002
