# Supplementary material for: Discovery of dihydrooxazolo[2,3-a]isoquinoliniums as highly specific inhibitors of hCE2
Source: RSC Adv. 2019 Nov 4;9(61):35904–12. doi: 10.1039/c9ra07457k (PMC9074662; doi:10.1039/c9ra07457k)
Supplement: RA-009-C9RA07457K-s001 [file RA-009-C9RA07457K-s001.pdf]

Electronic Supplementary Material (ESI) for RSC Advances.
This journal is © The Royal Society of Chemistry 2019

23d HREI-MS -02-79A1-0 -c1 2/26/2019 2:57:57 PM

EIH-2019022508-ZWL-DLX_DLX-02-79A1-0 -c1#11 RT: 7.85
T: + c EI Full ms [49.50-800.50]
m/z= 93-530

m/z	Intensity	Relative	Theo. Mass	Delta (mmu)	RDB equiv.	Composition
93.0069	64358.0	5.37	93.0057	1.19	1.0	$C_1H_3O_4N_1$
93.0710	103545.0	8.65	93.0699	1.15	3.5	C_7H_9
94.0420	41056.0	3.43	94.0413	0.65	4.0	$C_6H_6O_1$
94.0771	44034.0	3.68	94.0777	-0.63	3.0	C_7H_{10}
95.0864	271976.0	22.71	95.0855	0.88	2.5	C_7H_{11}
96.0202	42925.0	3.58	96.0206	-0.35	4.0	$C_5H_4O_2$
96.0929	129301.0	10.80	96.0934	-0.42	2.0	C_7H_{12}
97.0660	66227.0	5.53	97.0648	1.17	2.5	$C_6H_9O_1$
97.1014	551836.0	46.08	97.1012	0.22	1.5	C_7H_{13}
98.0361	41698.0	3.48	98.0362	-0.10	3.0	$C_5H_6O_2$
98.0729	153946.0	12.86	98.0726	0.28	2.0	$C_6H_{10}O_1$
98.1093	145361.0	12.14	98.1090	0.33	1.0	C_7H_{14}
99.1164	169539.0	14.16	99.1168	-0.39	0.5	C_7H_{15}
101.0592	54663.0	4.56	101.0597	-0.48	1.5	$C_5H_9O_2$
103.0534	45845.0	3.83	103.0542	-0.81	5.5	C_8H_7
104.0606	40297.0	3.37	104.0621	-1.41	5.0	C_8H_8
105.0336	62197.0	5.19	105.0335	0.08	5.5	$C_7H_5O_1$
105.0701	137360.0	11.47	105.0699	0.24	4.5	C_8H_9
107.0493	82404.0	6.88	107.0491	0.18	4.5	$C_7H_7O_1$
107.0857	66928.0	5.59	107.0855	0.15	3.5	C_8H_{11}
108.0576	113357.0	9.47	108.0570	0.67	4.0	$C_7H_8O_1$
109.1010	176431.0	14.73	109.1012	-0.20	2.5	C_8H_{13}
110.1097	65059.0	5.43	110.1090	0.69	2.0	C_8H_{14}
111.0810	66344.0	5.54	111.0804	0.57	2.5	$C_7H_{11}O_1$
111.1178	268471.0	22.42	111.1168	1.00	1.5	C_8H_{15}
112.1235	72943.0	6.09	112.1247	-1.13	1.0	C_8H_{16}
113.1326	115226.0	9.62	113.1325	0.07	0.5	C_8H_{17}
115.0540	95720.0	7.99	115.0542	-0.21	6.5	C_9H_7
115.0755	55072.0	4.60	115.0754	0.12	1.5	$C_6H_{11}O_2$
117.0693	67979.0	5.68	117.0699	-0.57	5.5	C_9H_9
119.0856	87660.0	7.32	119.0855	0.11	4.5	C_9H_{11}
120.0945	46312.0	3.87	120.0934	1.14	4.0	C_9H_{12}
121.0643	105648.0	8.82	121.0648	-0.53	4.5	$C_8H_9O_1$
121.1008	74753.0	6.24	121.1012	-0.41	3.5	C_9H_{13}
122.0730	46487.0	3.88	122.0726	0.39	4.0	$C_8H_{10}O_1$
123.1168	104947.0	8.76	123.1168	-0.08	2.5	C_9H_{15}
124.1240	54605.0	4.56	124.1247	-0.64	2.0	C_9H_{16}
125.1325	152311.0	12.72	125.1325	-0.01	1.5	C_9H_{17}
126.1397	54371.0	4.54	126.1403	-0.64	1.0	C_9H_{18}
127.1487	89120.0	7.44	127.1481	0.53	0.5	C_9H_{19}
128.0629	75338.0	6.29	128.0621	0.84	7.0	$C_{10}H_8$
129.0687	63657.0	5.32	129.0699	-1.19	6.5	$C_{10}H_9$
129.0915	150150.0	12.54	129.0910	0.51	1.5	$C_7H_{13}O_2$
131.0869	69147.0	5.77	131.0855	1.38	5.5	$C_{10}H_{11}$
132.0909	38778.0	3.24	132.0934	-2.50	5.0	$C_{10}H_{12}$
133.1013	43041.0	3.59	133.1012	0.15	4.5	$C_{10}H_{13}$
135.0799	53612.0	4.48	135.0804	-0.53	4.5	$C_9H_{11}O_1$
135.1162	51042.0	4.26	135.1168	-0.58	3.5	$C_{10}H_{15}$
137.1344	63248.0	5.28	137.1325	1.88	2.5	$C_{10}H_{17}$
138.1421	36792.0	3.07	138.1403	1.85	2.0	$C_{10}H_{18}$
139.1482	55831.0	4.66	139.1481	0.03	1.5	$C_{10}H_{19}$
141.1665	67161.0	5.61	141.1638	2.69	0.5	$C_{10}H_{21}$
147.0680	164984.0	13.78	147.0679	0.15	6.0	$C_9H_9O_1N_1$
147.0833	35975.0	3.00	147.0804	2.87	5.5	$C_{10}H_{11}O_1$
147.1184	84390.0	7.05	147.1168	1.59	4.5	$C_{11}H_{15}$

EIH-2019022508-ZWL-DLX_DLX-02-79A1-0 -c1#11 RT: 7.85
T: + c EI Full ms [49.50-800.50]
m/z= 93-530

m/z	Intensity	Relative	Theo. Mass	Delta (mmu)	RDB equiv.	Composition
149.1354	46779.0	3.91	149.1325	2.92	3.5	$C_{11}H_{17}$
151.1487	35274.0	2.95	151.1481	0.61	2.5	$C_{11}H_{19}$
153.1662	44910.0	3.75	153.1638	2.37	1.5	$C_{11}H_{21}$
155.1805	39304.0	3.28	155.1794	1.08	0.5	$C_{11}H_{23}$
157.1205	41406.0	3.46	157.1223	-1.82	1.5	$C_{9}H_{17}O_{2}$
161.0957	37201.0	3.11	161.0961	-0.39	5.5	$C_{11}H_{13}O_{1}$
163.1090	65117.0	5.44	163.1117	-2.78	4.5	$C_{11}H_{15}O_{1}$
171.1360	38895.0	3.25	171.1380	-1.98	1.5	$C_{10}H_{19}O_{2}$
175.1115	43801.0	3.66	175.1117	-0.23	5.5	$C_{12}H_{15}O_{1}$
175.1493	82871.0	6.92	175.1481	1.17	4.5	$C_{13}H_{19}$
185.1537	58284.0	4.87	185.1536	0.13	1.5	$C_{11}H_{21}O_{2}$
191.0018	136134.0	11.37	191.0002	1.61	13.0	$C_{12}H_{1}O_{2}N_{1}$
191.1443	660988.0	55.20	191.1430	1.26	4.5	$C_{13}H_{19}O_{1}$
192.1481	57700.0	4.82	192.1509	-2.76	4.0	$C_{13}H_{20}O_{1}$
206.1669	81762.0	6.83	206.1665	0.38	4.0	$C_{14}H_{22}O_{1}$
207.0325	1197465.0	100.00	207.0315	1.05	12.0	$C_{13}H_{5}O_{2}N_{1}$
208.0325	245052.0	20.46	208.0308	1.72	16.0	$C_{17}H_{4}$
209.0310	151902.0	12.69	209.0319	-0.86	7.0	$C_{9}H_{7}O_{5}N_{1}$
213.1857	46721.0	3.90	213.1849	0.75	1.5	$C_{13}H_{25}O_{2}$
221.0844	186067.0	15.54	221.0835	0.87	11.0	$C_{15}H_{11}O_{1}N_{1}$
222.0862	41873.0	3.50	222.0887	-2.41	6.0	$C_{12}H_{14}O_{4}$
223.0714	37143.0	3.10	223.0687	2.79	2.0	$C_{7}H_{13}O_{7}N_{1}$
265.0201	49933.0	4.17	265.0217	-1.59	9.0	$C_{11}H_{7}O_{7}N_{1}$
267.9996	39479.0	3.30	268.0002	-0.64	13.0	$C_{14}H_{4}O_{6}$
281.0511	412899.0	34.48	281.0530	-1.87	8.0	$C_{12}H_{11}O_{7}N_{1}$
282.0521	133097.0	11.11	282.0523	-0.21	12.0	$C_{16}H_{10}O_{5}$
283.0530	89646.0	7.49	283.0542	-1.23	20.5	$C_{23}H_{7}$
295.1010	54079.0	4.52	295.0992	1.88	16.0	$C_{21}H_{13}O_{1}N_{1}$
324.1845	45728.0	3.82	324.1873	-2.77	14.0	$C_{25}H_{24}$
342.0191	43625.0	3.64	342.0186	0.54	22.5	$C_{23}H_{4}O_{3}N_{1}$
355.0765	240264.0	20.06	355.0754	1.15	21.5	$C_{26}H_{11}O_{2}$
356.0766	90464.0	7.55	356.0765	0.10	12.5	$C_{18}H_{14}O_{7}N_{1}$
357.0742	49174.0	4.11	357.0758	-1.54	16.5	$C_{22}H_{13}O_{5}$
367.1073	51393.0	4.29	367.1050	2.23	13.0	$C_{20}H_{17}O_{6}N_{1}$
369.1198	45494.0	3.80	369.1207	-0.91	12.0	$C_{20}H_{19}O_{6}N_{1}$
385.1964	38837.0	3.24	385.1951	1.31	18.5	$C_{30}H_{25}$
415.0371	36325.0	3.03	415.0390	-1.82	27.5	$C_{30}H_{7}O_{3}$
423.1605	48765.0	4.07	423.1591	1.38	17.5	$C_{28}H_{23}O_{4}$
429.0833	107867.0	9.01	429.0843	-0.99	18.0	$C_{24}H_{15}O_{7}N_{1}$
430.0846	47714.0	3.98	430.0836	1.01	22.0	$C_{28}H_{14}O_{5}$
431.0806	41815.0	3.49	431.0788	1.77	22.0	$C_{27}H_{13}O_{5}N_{1}$
439.2705	50867.0	4.25	439.2717	-1.17	10.0	$C_{27}H_{37}O_{4}N_{1}$
442.2926	59511.0	4.97	442.2925	0.06	4.0	$C_{24}H_{42}O_{7}$
503.0990	40355.0	3.37	503.1000	-0.98	23.0	$C_{30}H_{17}O_{7}N_{1}$

EIH-2018122408-ZWL_DLX-02-79A2 -c1#2 RT: 6.20
T: + c EI Full ms [49.50-800.50]
m/z= 48-803

m/z	Intensity	Relative	Theo. Mass	Delta (mmu)	RDB equiv.	Composition
50.0020	54671.0	6.12	50.0025	-0.48	4.5	$C_3 N_1$
50.9908	8566.0	0.96	50.9899	0.87	-0.5	$H_3 O_1 {}^{32}S_1$
51.0226	68617.0	7.68	51.0229	-0.36	3.5	$C_4 H_3$
51.9919	277480.0	31.08	51.9944	-2.42	4.0	$C_3 O_1$
52.0337	8566.0	0.96	52.0308	2.97	3.0	$C_4 H_4$
55.0023	15290.0	1.71	55.0053	-2.99	3.0	$C_2 H_1 O_1 N_1$
58.0770	6344.0	0.71	58.0777	-0.70	0.0	$C_4 H_{10}$
59.0157	4707.0	0.53	59.0128	2.98	1.5	$C_2 H_3 O_2$
59.0520	24353.0	2.73	59.0491	2.88	0.5	$C_3 H_7 O_1$
60.0019	6256.0	0.70	60.0028	-0.95	1.0	$C_2 H_4 {}^{32}S_1$
60.0229	150946.0	16.91	60.0206	2.36	1.0	$C_2 H_4 O_2$
61.0093	17307.0	1.94	61.0106	-1.39	0.5	$C_2 H_5 {}^{32}S_1$
61.0304	14296.0	1.60	61.0284	2.03	0.5	$C_2 H_5 O_2$
61.9977	10671.0	1.20	61.9998	-2.12	1.0	$C_1 H_2 O_3$
62.0164	28739.0	3.22	62.0151	1.34	5.0	$C_5 H_2$
63.0239	71423.0	8.00	63.0229	0.97	4.5	$C_5 H_3$
63.9620	38387.0	4.30	63.9614	0.67	1.0	$O_2 {}^{32}S_1$
64.0316	12454.0	1.39	64.0308	0.83	4.0	$C_5 H_4$
65.0392	41720.0	4.67	65.0386	0.67	3.5	$C_5 H_5$
66.0465	7601.0	0.85	66.0464	0.11	3.0	$C_5 H_6$
67.0549	38942.0	4.36	67.0542	0.63	2.5	$C_5 H_7$
68.0617	13653.0	1.53	68.0621	-0.34	2.0	$C_5 H_8$
69.0335	9706.0	1.09	69.0335	-0.02	2.5	$C_4 H_5 O_1$
69.0700	77066.0	8.63	69.0699	0.15	1.5	$C_5 H_9$
70.0398	7484.0	0.84	70.0413	-1.55	2.0	$C_4 H_6 O_1$
70.0761	30522.0	3.42	70.0777	-1.61	1.0	$C_5 H_{10}$
71.0473	26254.0	2.94	71.0491	-1.83	1.5	$C_4 H_7 O_1$
71.0837	63150.0	7.07	71.0855	-1.79	0.5	$C_5 H_{11}$
72.0557	4853.0	0.54	72.0570	-1.26	1.0	$C_4 H_8 O_1$
73.0063	4414.0	0.49	73.0073	-1.00	6.5	$C_6 H_1$
73.0271	108144.0	12.11	73.0284	-1.29	1.5	$C_3 H_5 O_2$
73.0635	8303.0	0.93	73.0648	-1.28	0.5	$C_4 H_9 O_1$
74.0136	23242.0	2.60	74.0151	-1.51	6.0	$C_6 H_2$
74.0349	25727.0	2.88	74.0362	-1.32	1.0	$C_3 H_6 O_2$
75.0222	28826.0	3.23	75.0229	-0.75	5.5	$C_6 H_3$
75.0428	19442.0	2.18	75.0441	-1.22	0.5	$C_3 H_7 O_2$
76.0306	30317.0	3.40	76.0308	-0.14	5.0	$C_6 H_4$
77.0040	16576.0	1.86	77.0056	-1.53	0.5	$C_2 H_5 O_1 {}^{32}S_1$
77.0378	108349.0	12.13	77.0386	-0.73	4.5	$C_6 H_5$
78.0461	16547.0	1.85	78.0464	-0.34	4.0	$C_6 H_6$
79.0539	29206.0	3.27	79.0542	-0.28	3.5	$C_6 H_7$
80.0614	4794.0	0.54	80.0621	-0.63	3.0	$C_6 H_8$
81.0705	30990.0	3.47	81.0699	0.60	2.5	$C_6 H_9$
82.0772	17073.0	1.91	82.0777	-0.50	2.0	$C_6 H_{10}$
83.0490	21371.0	2.39	83.0491	-0.10	2.5	$C_5 H_7 O_1$
83.0855	58443.0	6.55	83.0855	-0.03	1.5	$C_6 H_{11}$
84.0567	10028.0	1.12	84.0570	-0.30	2.0	$C_5 H_8 O_1$
84.0925	11635.0	1.30	84.0934	-0.89	1.0	$C_6 H_{12}$
85.0285	8858.0	0.99	85.0284	0.11	2.5	$C_4 H_5 O_2$
85.0642	4940.0	0.55	85.0648	-0.56	1.5	$C_5 H_9 O_1$
85.1015	31516.0	3.53	85.1012	0.32	0.5	$C_6 H_{13}$
86.0146	8420.0	0.94	86.0151	-0.48	7.0	$C_7 H_2$
87.0225	10466.0	1.17	87.0229	-0.47	6.5	$C_7 H_3$
87.0437	32510.0	3.64	87.0441	-0.33	1.5	$C_4 H_7 O_2$

EIH-2018122408-ZWL_DLX-02-79A2 -c1#2 RT: 6.20
T: + c EI Full ms [49.50-800.50]
m/z= 48-803

m/z	Intensity	Relative	Theo. Mass	Delta (mmu)	RDB equiv.	Composition
88.0302	8536.0	0.96	88.0308	-0.58	6.0	C_7H_4
88.0519	13799.0	1.55	88.0519	-0.03	1.0	$C_4H_8O_2$
89.0385	96040.0	10.76	89.0386	-0.11	5.5	C_7H_5
90.0464	36545.0	4.09	90.0464	0.02	5.0	C_7H_6
91.0542	71277.0	7.98	91.0542	0.01	4.5	C_7H_7
92.0603	5496.0	0.62	92.0621	-1.79	4.0	C_7H_8
93.0329	6519.0	0.73	93.0335	-0.63	4.5	$C_6H_5O_1$
93.0688	7718.0	0.86	93.0699	-1.11	3.5	C_7H_9
95.0495	6315.0	0.71	95.0491	0.41	3.5	$C_6H_7O_1$
95.0852	24587.0	2.75	95.0855	-0.31	2.5	C_7H_{11}
96.0921	8010.0	0.90	96.0934	-1.22	2.0	C_7H_{12}
97.0655	8010.0	0.90	97.0648	0.73	2.5	$C_6H_9O_1$
97.1014	29879.0	3.35	97.1012	0.20	1.5	C_7H_{13}
98.0732	10291.0	1.15	98.0726	0.59	2.0	$C_6H_{10}O_1$
98.1092	8098.0	0.91	98.1090	0.24	1.0	C_7H_{14}
99.0441	6841.0	0.77	99.0441	0.01	2.5	$C_5H_7O_2$
99.1174	7045.0	0.79	99.1168	0.54	0.5	C_7H_{15}
100.0523	4531.0	0.51	100.0519	0.46	2.0	$C_5H_8O_2$
101.0384	5525.0	0.62	101.0386	-0.13	6.5	C_8H_5
101.0606	16050.0	1.80	101.0597	0.86	1.5	$C_5H_9O_2$
102.0473	4794.0	0.54	102.0464	0.88	6.0	C_8H_6
103.0546	8449.0	0.95	103.0542	0.39	5.5	C_8H_7
104.0270	14998.0	1.68	104.0257	1.34	6.0	$C_7H_4O_1$
105.0353	55577.0	6.22	105.0369	-1.59	0.5	$C_4H_9O_1{}^{32}S_1$
105.0726	10320.0	1.16	105.0732	-0.67	-0.5	$C_5H_{13}{}^{32}S_1$
106.0401	6110.0	0.68	106.0413	-1.18	5.0	$C_7H_6O_1$
107.0516	114196.0	12.79	107.0525	-0.90	-0.5	$C_4H_{11}O_1{}^{32}S_1$
107.0880	4911.0	0.55	107.0855	2.49	3.5	C_8H_{11}
108.0562	9852.0	1.10	108.0570	-0.75	4.0	$C_7H_8O_1$
108.9935	4853.0	0.54	108.9920	1.44	5.5	$C_5H_1O_3$
109.0677	4414.0	0.49	109.0648	2.88	3.5	$C_7H_9O_1$
113.0160	4823.0	0.54	113.0141	1.90	-1.0	$C_1H_7O_3N_1{}^{32}S_1$
113.0406	7133.0	0.80	113.0419	-1.39	2.5	$C_6H_9{}^{32}S_1$
114.0477	7923.0	0.89	114.0464	1.31	7.0	C_9H_6
115.0553	31487.0	3.53	115.0542	1.07	6.5	C_9H_7
115.0760	12746.0	1.43	115.0754	0.69	1.5	$C_6H_{11}O_2$
116.0501	8507.0	0.95	116.0495	0.58	6.5	$C_8H_6N_1$
121.0116	5028.0	0.56	121.0106	0.96	5.5	$C_7H_5{}^{32}S_1$
121.0273	14530.0	1.63	121.0284	-1.07	5.5	$C_7H_5O_2$
121.1013	5993.0	0.67	121.1012	0.12	3.5	C_9H_{13}
122.0354	31487.0	3.53	122.0362	-0.81	5.0	$C_7H_6O_2$
125.0151	96625.0	10.82	125.0141	0.98	0.0	$C_2H_7O_3N_1{}^{32}S_1$
126.0192	6081.0	0.68	126.0186	0.60	4.5	$C_5H_4O_3N_1$
126.0436	8361.0	0.94	126.0464	-2.76	8.0	$C_{10}H_6$
127.0113	27920.0	3.13	127.0111	0.12	0.0	$C_1H_5O_6N_1$
127.0527	5934.0	0.66	127.0542	-1.54	7.5	$C_{10}H_7$
128.0504	5671.0	0.64	128.0495	0.91	7.5	$C_9H_6N_1$
128.0594	5116.0	0.57	128.0621	-2.69	7.0	$C_{10}H_8$
129.0557	42714.0	4.78	129.0546	1.05	2.5	$C_6H_9O_3$
129.0698	5233.0	0.59	129.0699	-0.12	6.5	$C_{10}H_9$
129.0907	17132.0	1.92	129.0910	-0.35	1.5	$C_7H_{13}O_2$
130.0613	4707.0	0.53	130.0624	-1.17	2.0	$C_6H_{10}O_3$
139.0541	10437.0	1.17	139.0542	-0.09	8.5	$C_{11}H_7$
140.0496	7513.0	0.84	140.0495	0.15	8.5	$C_{10}H_6N_1$

EIH-2018122408-ZWL_DLX-02-79A2 -c1#2 RT: 6.20
T: + c EI Full ms [49.50-800.50]
m/z= 48-803

m/z	Intensity	Relative	Theo. Mass	Delta (mmu)	RDB equiv.	Composition
141.0577	15524.0	1.74	141.0573	0.40	8.0	$C_{10}H_7N_1$
141.0692	6402.0	0.72	141.0699	-0.72	7.5	$C_{11}H_9$
142.0150	8712.0	0.98	142.0135	1.50	4.5	$C_5H_4O_4N_1$
142.0651	70605.0	7.91	142.0651	-0.05	7.5	$C_{10}H_8N_1$
143.0706	36603.0	4.10	143.0703	0.35	2.5	$C_7H_{11}O_3$
146.9849	4970.0	0.56	146.9832	1.68	-1.0	$H_5O_6N_1{}^{32}S_1$
149.0232	85486.0	9.57	149.0233	-0.12	6.5	$C_8H_5O_3$
150.0282	5320.0	0.60	150.0311	-2.95	6.0	$C_8H_6O_3$
150.0457	9472.0	1.06	150.0464	-0.69	10.0	$C_{12}H_6$
151.0535	12922.0	1.45	151.0542	-0.73	9.5	$C_{12}H_7$
152.0621	13302.0	1.49	152.0621	0.09	9.0	$C_{12}H_8$
153.0584	8887.0	1.00	153.0580	0.42	-0.5	$C_5H_{13}O_3{}^{32}S_1$
153.0692	11168.0	1.25	153.0699	-0.70	8.5	$C_{12}H_9$
154.0667	6402.0	0.72	154.0658	0.86	-1.0	$C_5H_{14}O_3{}^{32}S_1$
155.0146	7016.0	0.79	155.0161	-1.55	4.5	$C_7H_7O_2{}^{32}S_1$
157.1215	4794.0	0.54	157.1223	-0.77	1.5	$C_9H_{17}O_2$
164.0495	8244.0	0.92	164.0495	-0.02	10.5	$C_{12}H_6N_1$
164.0615	5788.0	0.65	164.0621	-0.60	10.0	$C_{13}H_8$
165.0694	9677.0	1.08	165.0699	-0.53	9.5	$C_{13}H_9$
166.0654	8156.0	0.91	166.0651	0.31	9.5	$C_{12}H_8N_1$
167.0334	5028.0	0.56	167.0339	-0.47	5.5	$C_8H_7O_4$
167.0744	5701.0	0.64	167.0736	0.79	-0.5	$C_6H_{15}O_3{}^{32}S_1$
169.0311	25113.0	2.81	169.0318	-0.66	4.5	$C_8H_9O_2{}^{32}S_1$
170.0389	7367.0	0.83	170.0396	-0.67	4.0	$C_8H_{10}O_2{}^{32}S_1$
176.0547	4736.0	0.53	176.0553	-0.62	2.5	$C_6H_{10}O_5N_1$
177.0574	7045.0	0.79	177.0573	0.10	11.0	$C_{13}H_7N_1$
178.0603	9881.0	1.11	178.0624	-2.18	6.0	$C_{10}H_{10}O_3$
178.0634	10086.0	1.13	178.0624	0.96	6.0	$C_{10}H_{10}O_3$
185.1180	4970.0	0.56	185.1172	0.76	2.5	$C_{10}H_{17}O_3$
188.9782	47040.0	5.27	188.9793	-1.19	13.5	$C_{13}H_1{}^{32}S_1$
190.0660	10700.0	1.20	190.0658	0.19	2.0	$C_8H_{14}O_3{}^{32}S_1$
190.9749	15904.0	1.78	190.9730	1.84	0.0	$C_1H_5O_8N_1{}^{32}S_1$
191.0757	5701.0	0.64	191.0763	-0.61	6.0	$C_{11}H_{13}N_1{}^{32}S_1$
191.1444	13624.0	1.53	191.1430	1.35	4.5	$C_{13}H_{19}O_1$
193.0496	5584.0	0.63	193.0495	0.02	6.5	$C_{10}H_9O_4$
195.0687	4970.0	0.56	195.0686	0.18	0.5	$C_7H_{15}O_4{}^{32}S_1$
202.0640	5584.0	0.63	202.0651	-1.11	12.5	$C_{15}H_8N_1$
203.0728	5262.0	0.59	203.0730	-0.19	12.0	$C_{15}H_9N_1$
204.0003	19500.0	2.18	203.9995	0.81	18.0	C_{17}
204.0791	6490.0	0.73	204.0781	0.96	7.0	$C_{12}H_{12}O_3$
206.0592	6695.0	0.75	206.0600	-0.84	11.5	$C_{14}H_8O_1N_1$
207.0656	6987.0	0.78	207.0652	0.41	6.5	$C_{11}H_{11}O_4$
220.0752	6519.0	0.73	220.0757	-0.52	11.5	$C_{15}H_{10}O_1N_1$
222.0543	6548.0	0.73	222.0550	-0.63	11.5	$C_{14}H_8O_2N_1$
223.0627	4677.0	0.52	223.0628	-0.05	11.0	$C_{14}H_9O_2N_1$
224.0689	4707.0	0.53	224.0679	0.94	6.0	$C_{11}H_{12}O_5$
231.0682	4560.0	0.51	231.0679	0.33	13.0	$C_{16}H_9O_1N_1$
235.0652	7162.0	0.80	235.0662	-0.97	7.0	$C_{12}H_{13}O_2N_1{}^{32}S_1$
236.0731	7601.0	0.85	236.0740	-0.83	6.5	$C_{12}H_{14}O_2N_1{}^{32}S_1$
237.0748	7396.0	0.83	237.0758	-0.98	6.5	$C_{12}H_{13}O_5$
247.0624	6139.0	0.69	247.0628	-0.36	13.0	$C_{16}H_9O_2N_1$
248.0694	5554.0	0.62	248.0706	-1.20	12.5	$C_{16}H_{10}O_2N_1$
249.0744	5204.0	0.58	249.0732	1.15	11.5	$C_{17}H_{13}{}^{32}S_1$
250.0456	7542.0	0.84	250.0447	0.88	12.0	$C_{16}H_{10}O_1{}^{32}S_1$

EIH-2018122408-ZWL_DLX-02-79A2 -c1#2 RT: 6.20
T: + c EI Full ms [49.50-800.50]
m/z= 48-803

m/z	Intensity	Relative	Theo. Mass	Delta (mmu)	RDB equiv.	Composition
250.0798	5876.0	0.66	250.0811	-1.26	11.0	$C_{17} H_{14} {}^{32}S_1$
251.0538	6315.0	0.71	251.0525	1.25	11.5	$C_{16} H_{11} O_1 {}^{32}S_1$
252.0651	6841.0	0.77	252.0655	-0.42	11.5	$C_{15} H_{10} O_3 N_1$
254.0813	5554.0	0.62	254.0812	0.15	10.5	$C_{15} H_{12} O_3 N_1$
264.0662	12483.0	1.40	264.0662	-0.02	3.0	$C_{10} H_{16} O_6 {}^{32}S_1$
265.0755	5817.0	0.65	265.0767	-1.26	7.0	$C_{13} H_{15} O_3 N_1 {}^{32}S_1$
266.0831	8800.0	0.99	266.0819	1.24	2.0	$C_{10} H_{18} O_6 {}^{32}S_1$
267.0855	6168.0	0.69	267.0863	-0.85	6.5	$C_{13} H_{15} O_6$
268.0963	10349.0	1.16	268.0968	-0.50	10.5	$C_{16} H_{14} O_3 N_1$
277.0745	5847.0	0.65	277.0740	0.45	3.5	$C_{11} H_{17} O_6 {}^{32}S_1$
278.0808	11372.0	1.27	278.0812	-0.39	12.5	$C_{17} H_{12} O_3 N_1$
280.0603	6402.0	0.72	280.0604	-0.16	12.5	$C_{16} H_{10} O_4 N_1$
280.0929	7250.0	0.81	280.0941	-1.21	7.0	$C_{14} H_{16} O_6$
281.0658	8273.0	0.93	281.0656	0.25	7.5	$C_{13} H_{13} O_7$
282.0779	11402.0	1.28	282.0768	1.16	2.0	$C_{10} H_{18} O_7 {}^{32}S_1$
283.0850	4414.0	0.49	283.0846	0.36	1.5	$C_{10} H_{19} O_7 {}^{32}S_1$
291.0676	15670.0	1.76	291.0679	-0.30	18.0	$C_{21} H_9 O_1 N_1$
292.0617	12893.0	1.44	292.0611	0.58	4.0	$C_{11} H_{16} O_7 {}^{32}S_1$
293.0668	8683.0	0.97	293.0656	1.25	8.5	$C_{14} H_{13} O_7$
294.0754	16079.0	1.80	294.0761	-0.69	12.5	$C_{17} H_{12} O_4 N_1$
295.0772	5408.0	0.61	295.0787	-1.51	11.5	$C_{18} H_{15} O_2 {}^{32}S_1$
296.0915	16518.0	1.85	296.0917	-0.22	11.5	$C_{17} H_{14} O_4 N_1$
297.1020	8186.0	0.92	297.1029	-0.96	6.0	$C_{14} H_{19} O_4 N_1 {}^{32}S_1$
298.1059	6636.0	0.74	298.1047	1.16	6.0	$C_{14} H_{18} O_7$
306.0764	15612.0	1.75	306.0761	0.29	13.5	$C_{18} H_{12} O_4 N_1$
308.0912	21868.0	2.45	308.0917	-0.52	12.5	$C_{18} H_{14} O_4 N_1$
309.0956	7864.0	0.88	309.0969	-1.23	7.5	$C_{15} H_{17} O_7$
310.0719	11372.0	1.27	310.0717	0.17	3.0	$C_{11} H_{18} O_8 {}^{32}S_1$
310.0994	6578.0	0.74	310.0988	0.54	16.0	$C_{22} H_{14} O_2$
312.0830	7542.0	0.84	312.0840	-0.92	7.0	$C_{14} H_{16} O_8$
320.0849	6139.0	0.69	320.0866	-1.68	13.0	$C_{20} H_{16} O_2 {}^{32}S_1$
321.1000	4765.0	0.53	321.1003	-0.29	3.5	$C_{13} H_{21} O_7 {}^{32}S_1$
322.0733	7864.0	0.88	322.0744	-1.08	8.5	$C_{15} H_{16} O_5 N_1 {}^{32}S_1$
322.1043	16255.0	1.82	322.1047	-0.43	8.0	$C_{16} H_{18} O_7$
324.0842	12483.0	1.40	324.0841	0.07	16.5	$C_{22} H_{14} N_1 {}^{32}S_1$
325.0929	9267.0	1.04	325.0920	0.91	16.0	$C_{22} H_{15} N_1 {}^{32}S_1$
334.0736	7221.0	0.81	334.0744	-0.80	9.5	$C_{16} H_{16} O_5 N_1 {}^{32}S_1$
335.0805	7250.0	0.81	335.0795	1.02	4.5	$C_{13} H_{19} O_8 {}^{32}S_1$
336.0884	12951.0	1.45	336.0873	1.01	4.0	$C_{13} H_{20} O_8 {}^{32}S_1$
337.0933	17834.0	2.00	337.0945	-1.13	13.0	$C_{19} H_{15} O_5 N_1$
338.1026	10174.0	1.14	338.1023	0.32	12.5	$C_{19} H_{16} O_5 N_1$
350.1029	58179.0	6.52	350.1030	-0.10	4.0	$C_{14} H_{22} O_8 {}^{32}S_1$
351.1107	99666.0	11.16	351.1108	-0.10	3.5	$C_{14} H_{23} O_8 {}^{32}S_1$
352.1166	68441.0	7.67	352.1154	1.16	16.5	$C_{24} H_{18} N_1 {}^{32}S_1$
353.1192	15348.0	1.72	353.1199	-0.68	21.0	$C_{27} H_{15} N_1$
359.9842	11928.0	1.34	359.9842	-0.02	27.0	$C_{26} O_3$
366.9793	43561.0	4.88	366.9781	1.19	16.0	$C_{17} H_5 O_7 N_1 {}^{32}S_1$
367.1093	7923.0	0.89	367.1084	0.92	8.0	$C_{17} H_{21} O_6 N_1 {}^{32}S_1$
382.1317	29206.0	3.27	382.1319	-0.22	7.5	$C_{18} H_{24} O_6 N_1 {}^{32}S_1$
383.1303	8858.0	0.99	383.1305	-0.15	21.0	$C_{28} H_{17} O_1 N_1$
405.9789	6578.0	0.74	405.9778	1.14	18.0	$C_{20} H_6 O_8 {}^{32}S_1$
520.1422	112910.0	12.65	520.1425	-0.25	16.5	$C_{28} H_{26} O_7 N_1 {}^{32}S_1$
551.1607	5262.0	0.59	551.1608	-0.14	16.0	$C_{29} H_{29} O_8 N_1 {}^{32}S_1$

m/z	Intensity	Relative	Theo. Mass	Delta (mmu)	RDB equiv.	Composition

23f HREI-MS _DLX-02-59A1 -c1 12/6/2018 4:22:23 PM

EIH-2018120512-ZWL-DLX_DLX-02-59A1 -c1#2 RT: 7.85
T: + c EI Full ms [49.50-800.50]
m/z= 48-803

m/z	Intensity	Relative	Theo. Mass	Delta (mmu)	RDB equiv.	Composition
51.9964	556426.0	30.58	51.9944	2.02	4.0	$C_3 O_1$
62.0170	118269.0	6.50	62.0151	1.92	5.0	$C_5 H_2$
63.0248	273752.0	15.04	63.0229	1.90	4.5	$C_5 H_3$
65.0393	155834.0	8.56	65.0386	0.70	3.5	$C_5 H_5$
75.0220	115335.0	6.34	75.0229	-0.89	5.5	$C_6 H_3$
75.0435	235688.0	12.95	75.0441	-0.60	0.5	$C_3 H_7 O_2$
77.0380	209481.0	11.51	77.0386	-0.55	4.5	$C_6 H_5$
89.0383	295733.0	16.25	89.0386	-0.29	5.5	$C_7 H_5$
90.0466	371655.0	20.42	90.0464	0.16	5.0	$C_7 H_6$
91.0539	429880.0	23.62	91.0542	-0.33	4.5	$C_7 H_7$
115.0542	120148.0	6.60	115.0542	0.01	6.5	$C_9 H_7$
118.0412	278917.0	15.33	118.0413	-0.16	6.0	$C_8 H_6 O_1$
119.0483	382748.0	21.03	119.0491	-0.82	5.5	$C_8 H_7 O_1$
121.0640	449220.0	24.69	121.0648	-0.76	4.5	$C_8 H_9 O_1$
139.0542	114982.0	6.32	139.0542	-0.01	8.5	$C_{11} H_7$
149.0601	611892.0	33.63	149.0597	0.41	5.5	$C_9 H_9 O_2$
150.0662	143626.0	7.89	150.0675	-1.30	5.0	$C_9 H_{10} O_2$
178.0646	115511.0	6.35	178.0651	-0.49	10.5	$C_{13} H_8 N_1$
191.1433	276833.0	15.21	191.1430	0.25	4.5	$C_{13} H_{19} O_1$
222.0546	119913.0	6.59	222.0550	-0.40	11.5	$C_{14} H_8 O_2 N_1$
236.0705	128746.0	7.08	236.0706	-0.15	11.5	$C_{15} H_{10} O_2 N_1$
264.0674	197566.0	10.86	264.0655	1.90	12.5	$C_{16} H_{10} O_3 N_1$
265.0736	137316.0	7.55	265.0733	0.25	12.0	$C_{16} H_{11} O_3 N_1$
278.0814	136846.0	7.52	278.0812	0.21	12.5	$C_{17} H_{12} O_3 N_1$
282.0755	174616.0	9.60	282.0761	-0.55	11.5	$C_{16} H_{12} O_4 N_1$
292.0591	191726.0	10.54	292.0604	-1.31	13.5	$C_{17} H_{10} O_4 N_1$
293.0662	180691.0	9.93	293.0656	0.62	8.5	$C_{14} H_{13} O_7$
294.0748	221073.0	12.15	294.0761	-1.24	12.5	$C_{17} H_{12} O_4 N_1$
296.0910	191139.0	10.50	296.0917	-0.73	11.5	$C_{17} H_{14} O_4 N_1$
306.0750	190405.0	10.46	306.0761	-1.08	13.5	$C_{18} H_{12} O_4 N_1$
307.0837	121908.0	6.70	307.0839	-0.24	13.0	$C_{18} H_{13} O_4 N_1$
308.0906	338375.0	18.60	308.0917	-1.11	12.5	$C_{18} H_{14} O_4 N_1$
309.0956	118357.0	6.50	309.0969	-1.29	7.5	$C_{15} H_{17} O_7$
310.0713	115569.0	6.35	310.0710	0.31	12.5	$C_{17} H_{12} O_5 N_1$
321.0968	117037.0	6.43	321.0969	-0.11	8.5	$C_{16} H_{17} O_7$
322.0714	165196.0	9.08	322.0710	0.43	13.5	$C_{18} H_{12} O_5 N_1$
322.1054	261895.0	14.39	322.1047	0.72	8.0	$C_{16} H_{18} O_7$
324.0860	292387.0	16.07	324.0866	-0.70	12.5	$C_{18} H_{14} O_5 N_1$
325.0924	117888.0	6.48	325.0918	0.63	7.5	$C_{15} H_{17} O_8$
336.0860	302923.0	16.65	336.0866	-0.61	13.5	$C_{19} H_{14} O_5 N_1$
337.0922	294823.0	16.20	337.0918	0.44	8.5	$C_{16} H_{17} O_8$
338.1015	208043.0	11.43	338.1023	-0.79	12.5	$C_{19} H_{16} O_5 N_1$
350.1019	898851.0	49.40	350.1023	-0.38	13.5	$C_{20} H_{16} O_5 N_1$
351.1093	1229067.0	67.54	351.1101	-0.84	13.0	$C_{20} H_{17} O_5 N_1$
352.1167	970165.0	53.32	352.1179	-1.24	12.5	$C_{20} H_{18} O_5 N_1$
353.1206	212504.0	11.68	353.1199	0.72	21.0	$C_{27} H_{15} N_1$
367.1127	155100.0	8.52	367.1117	0.96	21.5	$C_{28} H_{15} O_1$
368.1174	122672.0	6.74	368.1196	-2.17	21.0	$C_{28} H_{16} O_1$
382.1277	458464.0	25.20	382.1285	-0.82	12.5	$C_{21} H_{20} O_6 N_1$
383.1328	159209.0	8.75	383.1305	2.35	21.0	$C_{28} H_{17} O_1 N_1$
500.1692	1819654.0	100.00	500.1704	-1.20	17.5	$C_{29} H_{26} O_7 N_1$
517.1739	177668.0	9.76	517.1731	0.80	17.0	$C_{29} H_{27} O_8 N_1$
531.1882	120735.0	6.64	531.1888	-0.54	17.0	$C_{30} H_{29} O_8 N_1$

m/z	Intensity	Relative	Theo. Mass	Delta (mmu)	RDB equiv.	Composition

23g HREI-MS

EIH-2018120513-ZWL-DLX_DLX-02-59A2-c1#6 RT: 8.05
T: + c EI Full ms [49.50-800.50]
m/z= 48-803

m/z	Intensity	Relative	Theo. Mass	Delta (mmu)	RDB equiv.	Composition
50.0019	158534.0	6.56	50.0025	-0.58	4.5	C_3N_1
51.0230	199356.0	8.25	51.0229	0.09	3.5	C_4H_3
51.9916	192694.0	7.97	51.9944	-2.73	4.0	C_3O_1
62.0165	173237.0	7.17	62.0151	1.38	5.0	C_5H_2
63.0240	368690.0	15.25	63.0229	1.05	4.5	C_5H_3
75.0221	185915.0	7.69	75.0229	-0.81	5.5	C_6H_3
75.0432	182687.0	7.56	75.0441	-0.86	0.5	$C_3H_7O_2$
76.0301	153457.0	6.35	76.0308	-0.63	5.0	C_6H_4
89.0386	594607.0	24.60	89.0386	0.00	5.5	C_7H_5
116.0488	816238.0	33.77	116.0495	-0.66	6.5	$C_8H_6N_1$
117.0558	223362.0	9.24	117.0546	1.15	1.5	$C_5H_9O_3$
139.0547	154895.0	6.41	139.0542	0.49	8.5	$C_{11}H_7$
151.0540	169334.0	7.00	151.0542	-0.24	9.5	$C_{12}H_7$
164.0486	151168.0	6.25	164.0495	-0.83	10.5	$C_{12}H_6N_1$
178.0649	200736.0	8.30	178.0651	-0.26	10.5	$C_{13}H_8N_1$
206.0610	157360.0	6.51	206.0600	0.95	11.5	$C_{14}H_8O_1N_1$
222.0546	166282.0	6.88	222.0550	-0.38	11.5	$C_{14}H_8O_2N_1$
235.0620	159092.0	6.58	235.0628	-0.74	12.0	$C_{15}H_9O_2N_1$
236.0705	192577.0	7.97	236.0706	-0.06	11.5	$C_{15}H_{10}O_2N_1$
252.0649	152195.0	6.30	252.0655	-0.64	11.5	$C_{15}H_{10}O_3N_1$
264.0678	309379.0	12.80	264.0682	-0.40	17.0	$C_{19}H_8N_2$
265.0750	190112.0	7.86	265.0760	-1.01	16.5	$C_{19}H_9N_2$
266.0838	197595.0	8.17	266.0838	-0.07	16.0	$C_{19}H_{10}N_2$
268.0958	201939.0	8.35	268.0968	-1.04	10.5	$C_{16}H_{14}O_3N_1$
278.0814	242321.0	10.02	278.0812	0.19	12.5	$C_{17}H_{12}O_3N_1$
281.0672	160354.0	6.63	281.0683	-1.11	12.0	$C_{16}H_{11}O_4N_1$
282.0736	162907.0	6.74	282.0761	-2.47	11.5	$C_{16}H_{12}O_4N_1$
292.0602	345946.0	14.31	292.0604	-0.19	13.5	$C_{17}H_{10}O_4N_1$
293.0666	247075.0	10.22	293.0683	-1.71	13.0	$C_{17}H_{11}O_4N_1$
296.0906	294618.0	12.19	296.0917	-1.09	11.5	$C_{17}H_{14}O_4N_1$
306.0761	326782.0	13.52	306.0761	0.00	13.5	$C_{18}H_{12}O_4N_1$
307.0822	203758.0	8.43	307.0839	-1.74	13.0	$C_{18}H_{13}O_4N_1$
308.0915	412888.0	17.08	308.0917	-0.27	12.5	$C_{18}H_{14}O_4N_1$
321.0989	210860.0	8.72	321.0996	-0.65	13.0	$C_{19}H_{15}O_4N_1$
322.0721	162731.0	6.73	322.0710	1.09	13.5	$C_{18}H_{12}O_5N_1$
322.1079	482969.0	19.98	322.1074	0.48	12.5	$C_{19}H_{16}O_4N_1$
324.0877	385771.0	15.96	324.0866	1.09	12.5	$C_{18}H_{14}O_5N_1$
334.0711	186179.0	7.70	334.0710	0.14	14.5	$C_{19}H_{12}O_5N_1$
335.0774	202849.0	8.39	335.0788	-1.39	14.0	$C_{19}H_{13}O_5N_1$
336.0862	351346.0	14.53	336.0866	-0.43	13.5	$C_{19}H_{14}O_5N_1$
337.0939	244551.0	10.12	337.0945	-0.62	13.0	$C_{19}H_{15}O_5N_1$
338.1009	194015.0	8.03	338.1023	-1.39	12.5	$C_{19}H_{16}O_5N_1$
350.1020	1669718.0	69.07	350.1023	-0.26	13.5	$C_{20}H_{16}O_5N_1$
351.1092	2229725.0	92.24	351.1101	-0.94	13.0	$C_{20}H_{17}O_5N_1$
352.1152	1207585.0	49.96	352.1179	-2.75	12.5	$C_{20}H_{18}O_5N_1$
353.1197	248630.0	10.29	353.1199	-0.20	21.0	$C_{27}H_{15}N_1$
367.1039	168835.0	6.98	367.1050	-1.18	13.0	$C_{20}H_{17}O_6N_1$
382.1282	577673.0	23.90	382.1285	-0.27	12.5	$C_{21}H_{20}O_6N_1$
383.1340	179958.0	7.44	383.1363	-2.30	12.0	$C_{21}H_{21}O_6N_1$
467.1605	2417342.0	100.00	467.1601	0.38	18.5	$C_{28}H_{23}O_5N_2$
468.1654	786392.0	32.53	468.1680	-2.57	18.0	$C_{28}H_{24}O_5N_2$
498.1785	203347.0	8.41	498.1785	-0.07	18.0	$C_{29}H_{26}O_6N_2$

EIH-2018120513-ZWL-DLX_DLX-02-59A2-c1#6 RT: 8.05
T: + c EI Full ms [49.50-800.50]
m/z= 48-803

m/z	Intensity	Relative	Theo. Mass	Delta (mmu)	RDB equiv.	Composition
50.0019	158534.0	6.56	50.0025	-0.58	4.5	$C_3 N_1$
51.0230	199356.0	8.25	51.0229	0.09	3.5	$C_4 H_3$
51.9916	192694.0	7.97	51.9944	-2.73	4.0	$C_3 O_1$
62.0165	173237.0	7.17	62.0151	1.38	5.0	$C_5 H_2$
63.0240	368690.0	15.25	63.0229	1.05	4.5	$C_5 H_3$
75.0221	185915.0	7.69	75.0229	-0.81	5.5	$C_6 H_3$
75.0432	182687.0	7.56	75.0441	-0.86	0.5	$C_3 H_7 O_2$
76.0301	153457.0	6.35	76.0308	-0.63	5.0	$C_6 H_4$
89.0386	594607.0	24.60	89.0386	0.00	5.5	$C_7 H_5$
116.0488	816238.0	33.77	116.0495	-0.66	6.5	$C_8 H_6 N_1$
117.0558	223362.0	9.24	117.0546	1.15	1.5	$C_5 H_9 O_3$
139.0547	154895.0	6.41	139.0542	0.49	8.5	$C_{11} H_7$
151.0540	169334.0	7.00	151.0542	-0.24	9.5	$C_{12} H_7$
164.0486	151168.0	6.25	164.0495	-0.83	10.5	$C_{12} H_6 N_1$
178.0649	200736.0	8.30	178.0651	-0.26	10.5	$C_{13} H_8 N_1$
206.0610	157360.0	6.51	206.0600	0.95	11.5	$C_{14} H_8 O_1 N_1$
222.0546	166282.0	6.88	222.0550	-0.38	11.5	$C_{14} H_8 O_2 N_1$
235.0620	159092.0	6.58	235.0628	-0.74	12.0	$C_{15} H_9 O_2 N_1$
236.0705	192577.0	7.97	236.0706	-0.06	11.5	$C_{15} H_{10} O_2 N_1$
252.0649	152195.0	6.30	252.0655	-0.64	11.5	$C_{15} H_{10} O_3 N_1$
264.0678	309379.0	12.80	264.0682	-0.40	17.0	$C_{19} H_8 N_2$
265.0750	190112.0	7.86	265.0760	-1.01	16.5	$C_{19} H_9 N_2$
266.0838	197595.0	8.17	266.0838	-0.07	16.0	$C_{19} H_{10} N_2$
268.0958	201939.0	8.35	268.0968	-1.04	10.5	$C_{16} H_{14} O_3 N_1$
278.0814	242321.0	10.02	278.0812	0.19	12.5	$C_{17} H_{12} O_3 N_1$
281.0672	160354.0	6.63	281.0683	-1.11	12.0	$C_{16} H_{11} O_4 N_1$
282.0736	162907.0	6.74	282.0761	-2.47	11.5	$C_{16} H_{12} O_4 N_1$
292.0602	345946.0	14.31	292.0604	-0.19	13.5	$C_{17} H_{10} O_4 N_1$
293.0666	247075.0	10.22	293.0683	-1.71	13.0	$C_{17} H_{11} O_4 N_1$
296.0906	294618.0	12.19	296.0917	-1.09	11.5	$C_{17} H_{14} O_4 N_1$
306.0761	326782.0	13.52	306.0761	0.00	13.5	$C_{18} H_{12} O_4 N_1$
307.0822	203758.0	8.43	307.0839	-1.74	13.0	$C_{18} H_{13} O_4 N_1$
308.0915	412888.0	17.08	308.0917	-0.27	12.5	$C_{18} H_{14} O_4 N_1$
321.0989	210860.0	8.72	321.0996	-0.65	13.0	$C_{19} H_{15} O_4 N_1$
322.0721	162731.0	6.73	322.0710	1.09	13.5	$C_{18} H_{12} O_5 N_1$
322.1079	482969.0	19.98	322.1074	0.48	12.5	$C_{19} H_{16} O_4 N_1$
324.0877	385771.0	15.96	324.0866	1.09	12.5	$C_{18} H_{14} O_5 N_1$
334.0711	186179.0	7.70	334.0710	0.14	14.5	$C_{19} H_{12} O_5 N_1$
335.0774	202849.0	8.39	335.0788	-1.39	14.0	$C_{19} H_{13} O_5 N_1$
336.0862	351346.0	14.53	336.0866	-0.43	13.5	$C_{19} H_{14} O_5 N_1$
337.0939	244551.0	10.12	337.0945	-0.62	13.0	$C_{19} H_{15} O_5 N_1$
338.1009	194015.0	8.03	338.1023	-1.39	12.5	$C_{19} H_{16} O_5 N_1$
350.1020	1669718.0	69.07	350.1023	-0.26	13.5	$C_{20} H_{16} O_5 N_1$
351.1092	2229725.0	92.24	351.1101	-0.94	13.0	$C_{20} H_{17} O_5 N_1$
352.1152	1207585.0	49.96	352.1179	-2.75	12.5	$C_{20} H_{18} O_5 N_1$
353.1197	248630.0	10.29	353.1199	-0.20	21.0	$C_{27} H_{15} N_1$
367.1039	168835.0	6.98	367.1050	-1.18	13.0	$C_{20} H_{17} O_6 N_1$
382.1282	577673.0	23.90	382.1285	-0.27	12.5	$C_{21} H_{20} O_6 N_1$
383.1340	179958.0	7.44	383.1363	-2.30	12.0	$C_{21} H_{21} O_6 N_1$
467.1605	2417342.0	100.00	467.1601	0.38	18.5	$C_{28} H_{23} O_5 N_2$
468.1654	786392.0	32.53	468.1680	-2.57	18.0	$C_{28} H_{24} O_5 N_2$
498.1785	203347.0	8.41	498.1785	-0.07	18.0	$C_{29} H_{26} O_6 N_2$

23h HREI-MS E0-DLX-02-51-0... 1/22/2019 3:59:29 PM

EIH-2019012107-ZWL-DLX_E0-DLX-02-51-0 -c1#35 RT: 20.86
T: + c EI Full ms [49.50-800.50]
m/z= 48-803

m/z	Intensity	Relative	Theo. Mass	Delta (mmu)	RDB equiv.	Composition
51.0319	1039216.0	15.74	51.0315	0.40	-1.0	$H_5 O_2 N_1$
65.0411	736663.0	11.16	65.0386	2.51	3.5	$C_5 H_5$
67.0554	582014.0	8.82	67.0542	1.15	2.5	$C_5 H_7$
75.0190	422213.0	6.40	75.0189	0.14	1.5	$C_1 H_3 O_2 N_2$
76.0270	540219.0	8.18	76.0267	0.32	1.0	$C_1 H_4 O_2 N_2$
77.0344	1261233.0	19.10	77.0346	-0.19	0.5	$C_1 H_5 O_2 N_2$
78.0426	1582553.0	23.97	78.0424	0.26	0.0	$C_1 H_6 O_2 N_2$
79.0508	708131.0	10.73	79.0502	0.62	-0.5	$C_1 H_7 O_2 N_2$
83.0828	1246474.0	18.88	83.0855	-2.75	1.5	$C_6 H_{11}$
89.0364	837111.0	12.68	89.0346	1.89	1.5	$C_2 H_5 O_2 N_2$
91.0542	995131.0	15.07	91.0542	-0.06	4.5	$C_7 H_7$
121.0530	735072.0	11.13	121.0522	0.78	5.0	$C_7 H_7 O_1 N_1$
132.0703	3678066.0	55.71	132.0682	2.14	6.0	$C_8 H_8 N_2$
133.0778	4218859.0	63.90	133.0760	1.73	5.5	$C_8 H_9 N_2$
152.0609	519640.0	7.87	152.0621	-1.15	9.0	$C_{12} H_8$
159.0924	715033.0	10.83	159.0917	0.70	6.5	$C_{10} H_{11} N_2$
165.0657	750626.0	11.37	165.0659	-0.20	5.5	$C_8 H_9 O_2 N_2$
178.0750	1082283.0	16.39	178.0737	1.31	6.0	$C_9 H_{10} O_2 N_2$
179.0824	1195168.0	18.10	179.0815	0.89	5.5	$C_9 H_{11} O_2 N_2$
183.0350	730460.0	11.06	183.0374	-2.33	1.0	$C_4 H_9 O_7 N_1$
189.0665	440916.0	6.68	189.0659	0.67	7.5	$C_{10} H_9 O_2 N_2$
190.0700	521325.0	7.90	190.0710	-0.98	2.5	$C_7 H_{12} O_5 N_1$
191.0816	608033.0	9.21	191.0815	0.05	6.5	$C_{10} H_{11} O_2 N_2$
199.1251	729442.0	11.05	199.1230	2.15	7.5	$C_{13} H_{15} N_2$
201.0483	641144.0	9.71	201.0479	0.38	0.0	$C_4 H_{11} O_8 N_1$
202.0752	562230.0	8.52	202.0737	1.55	8.0	$C_{11} H_{10} O_2 N_2$
203.0801	496897.0	7.53	203.0788	1.32	3.0	$C_8 H_{13} O_5 N_1$
205.1002	469384.0	7.11	205.1012	-0.94	10.5	$C_{16} H_{13}$
207.0380	472437.0	7.16	207.0374	0.65	3.0	$C_6 H_9 O_7 N_1$
207.0441	508761.0	7.71	207.0441	0.08	11.5	$C_{14} H_7 O_2$
207.0496	476858.0	7.22	207.0499	-0.29	2.5	$C_7 H_{11} O_7$
209.0505	710199.0	10.76	209.0530	-2.50	2.0	$C_6 H_{11} O_7 N_1$
214.1483	3831920.0	58.04	214.1465	1.81	7.0	$C_{14} H_{18} N_2$
215.1547	1160117.0	17.57	215.1543	0.40	6.5	$C_{14} H_{19} N_2$
222.0569	472342.0	7.15	222.0550	1.94	11.5	$C_{14} H_8 O_2 N_1$
223.0672	479117.0	7.26	223.0687	-1.44	2.0	$C_7 H_{13} O_7 N_1$
225.0810	509016.0	7.71	225.0784	2.62	10.0	$C_{14} H_{11} O_2 N_1$
227.0650	455547.0	6.90	227.0662	-1.23	5.5	$C_9 H_{11} O_5 N_2$
235.0625	491999.0	7.45	235.0628	-0.32	12.0	$C_{15} H_9 O_2 N_1$
235.1497	635896.0	9.63	235.1481	1.60	9.5	$C_{18} H_{19}$
236.0706	484111.0	7.33	236.0706	-0.00	11.5	$C_{15} H_{10} O_2 N_1$
241.0766	486146.0	7.36	241.0760	0.62	14.5	$C_{17} H_9 N_2$
243.0937	812269.0	12.30	243.0949	-1.19	0.0	$C_7 H_{17} O_8 N_1$
251.0579	444447.0	6.73	251.0577	0.22	12.0	$C_{15} H_9 O_3 N_1$
252.0700	457201.0	6.93	252.0714	-1.36	2.5	$C_8 H_{14} O_8 N_1$
253.0790	462386.0	7.00	253.0792	-0.21	2.0	$C_8 H_{15} O_8 N_1$
263.1808	2262344.0	34.27	263.1794	1.40	9.5	$C_{20} H_{23}$
264.0685	590857.0	8.95	264.0682	0.29	17.0	$C_{19} H_8 N_2$
264.1886	552274.0	8.37	264.1873	1.30	9.0	$C_{20} H_{24}$
265.0788	432646.0	6.55	265.0792	-0.40	3.0	$C_9 H_{15} O_8 N_1$
267.0971	594928.0	9.01	267.0975	-0.42	6.5	$C_{12} H_{15} O_5 N_2$
267.1321	509238.0	7.71	267.1313	0.85	1.0	$C_{10} H_{21} O_7 N_1$
268.0987	420273.0	6.37	268.0995	-0.81	15.0	$C_{19} H_{12} N_2$
269.1090	1021881.0	15.48	269.1105	-1.50	1.0	$C_9 H_{19} O_8 N_1$
278.2030	3727082.0	56.45	278.2029	0.12	9.0	$C_{21} H_{26}$

EIH-2019012107-ZWL-DLX_E0-DLX-02-51-0 -c1#35 RT: 20.86
T: + c EI Full ms [49.50-800.50]
m/z= 48-803

m/z	Intensity	Relative	Theo. Mass	Delta (mmu)	RDB equiv.	Composition
279.2040	883836.0	13.39	279.2040	-0.02	0.0	$C_{13}H_{29}O_5N_1$
280.0641	432805.0	6.56	280.0631	0.99	17.0	$C_{19}H_8O_1N_2$
282.0793	789495.0	11.96	282.0788	0.56	16.0	$C_{19}H_{10}O_1N_2$
283.1238	752566.0	11.40	283.1230	0.83	14.5	$C_{20}H_{15}N_2$
285.1351	769615.0	11.66	285.1359	-0.86	9.0	$C_{17}H_{19}O_3N_1$
285.1392	779635.0	11.81	285.1386	0.61	13.5	$C_{20}H_{17}N_2$
292.0597	524570.0	7.95	292.0604	-0.73	13.5	$C_{17}H_{10}O_4N_1$
293.0707	662296.0	10.03	293.0709	-0.21	17.5	$C_{20}H_9O_1N_2$
294.0768	861444.0	13.05	294.0761	0.71	12.5	$C_{17}H_{12}O_4N_1$
296.0909	510256.0	7.73	296.0917	-0.84	11.5	$C_{17}H_{14}O_4N_1$
297.0993	508602.0	7.70	297.0996	-0.28	11.0	$C_{17}H_{15}O_4N_1$
306.0761	449122.0	6.80	306.0761	0.04	13.5	$C_{18}H_{12}O_4N_1$
308.0890	1358341.0	20.57	308.0891	-0.06	8.0	$C_{15}H_{16}O_7$
322.0912	772605.0	11.70	322.0921	-0.92	8.5	$C_{15}H_{16}O_7N_1$
324.0915	613154.0	9.29	324.0934	-1.84	21.0	$C_{26}H_{12}$
336.0880	1499122.0	22.71	336.0866	1.33	13.5	$C_{19}H_{14}O_5N_1$
337.0920	1678166.0	25.42	337.0918	0.19	8.5	$C_{16}H_{17}O_8$
338.1011	601735.0	9.11	338.1023	-1.24	12.5	$C_{19}H_{16}O_5N_1$
350.1032	2014690.0	30.52	350.1023	0.90	13.5	$C_{20}H_{16}O_5N_1$
351.1091	2565502.0	38.86	351.1101	-0.99	13.0	$C_{20}H_{17}O_5N_1$
352.1157	6602167.0	100.00	352.1153	0.46	8.0	$C_{17}H_{20}O_8$
353.1205	1630519.0	24.70	353.1199	0.57	21.0	$C_{27}H_{15}N_1$
365.2071	568114.0	8.60	365.2071	0.03	6.5	$C_{19}H_{29}O_5N_2$
366.9859	428352.0	6.49	366.9873	-1.47	20.5	$C_{21}H_3O_7$
367.2225	6179127.0	93.59	367.2227	-0.25	5.5	$C_{19}H_{31}O_5N_2$
368.2261	1716049.0	25.99	368.2247	1.36	14.0	$C_{26}H_{28}N_2$
382.1313	815259.0	12.35	382.1312	0.16	17.0	$C_{24}H_{18}O_3N_2$
383.1395	587135.0	8.89	383.1390	0.45	16.5	$C_{24}H_{19}O_3N_2$
449.2986	2513973.0	38.08	449.2983	0.29	2.0	$C_{22}H_{43}O_8N_1$
450.3044	723462.0	10.96	450.3061	-1.76	1.5	$C_{22}H_{44}O_8N_1$
518.1688	444796.0	6.74	518.1684	0.42	17.0	$C_{28}H_{26}O_8N_2$

23i HREI-MS LX_DLX-02-60A1 -c2 12/11/2018 2:08:38 PM

EIH-2018120606-ZWL-DLX_DLX-02-60A1 -c2#5 RT: 6.81
T: + c EI Full ms [49.50-800.50]
m/z= 48-803

m/z	Intensity	Relative	Theo. Mass	Delta (mmu)	RDB equiv.	Composition
51.9926	222893.0	7.73	51.9944	-1.78	4.0	C_3O_1
63.0236	180955.0	6.28	63.0241	-0.48	0.5	$C_2H_4O_1F_1$
75.0439	194396.0	6.74	75.0441	-0.14	0.5	$C_3H_7O_2$
83.0861	359593.0	12.47	83.0855	0.56	1.5	C_6H_{11}
109.0481	345888.0	12.00	109.0495	-1.40	-0.5	$C_3H_9O_4$
159.0394	599478.0	20.79	159.0416	-2.22	4.5	$C_8H_6F_3$
159.0914	206458.0	7.16	159.0890	2.39	2.0	$C_7H_{13}O_3N_1$
165.0688	187529.0	6.50	165.0699	-1.03	9.5	$C_{13}H_9$
179.0840	240296.0	8.33	179.0855	-1.53	9.5	$C_{14}H_{11}$
183.0356	191022.0	6.63	183.0338	1.86	2.0	$C_5H_7O_4N_1F_2$
199.1244	207867.0	7.21	199.1214	2.97	-1.0	$C_7H_{18}O_4N_1F_1$
201.0459	165431.0	5.74	201.0443	1.56	1.0	$C_5H_9O_5N_1F_2$
209.0519	177082.0	6.14	209.0506	1.29	-1.0	$C_4H_{10}O_5N_1F_3$
215.1532	330803.0	11.47	215.1516	1.62	2.0	$C_{11}H_{21}O_3N_1$
243.0925	214646.0	7.45	243.0913	1.20	1.0	$C_8H_{15}O_5N_1F_2$
263.1819	600799.0	20.84	263.1817	0.23	1.5	$C_{14}H_{25}O_2F_2$
264.0670	233517.0	8.10	264.0667	0.33	8.5	$C_{13}H_{11}O_4N_1F_1$
268.0962	226913.0	7.87	268.0968	-0.62	10.5	$C_{16}H_{14}O_3N_1$
269.1067	233869.0	8.11	269.1069	-0.19	2.0	$C_{10}H_{17}O_5N_1F_2$
278.0808	203259.0	7.05	278.0812	-0.35	12.5	$C_{17}H_{12}O_3N_1$
278.2026	1107393.0	38.41	278.2029	-0.29	9.0	$C_{21}H_{26}$
283.1241	196715.0	6.82	283.1226	1.54	2.0	$C_{11}H_{19}O_5N_1F_2$
285.1385	274955.0	9.54	285.1382	0.32	1.0	$C_{11}H_{21}O_5N_1F_2$
292.0600	265417.0	9.21	292.0604	-0.40	13.5	$C_{17}H_{10}O_4N_1$
294.0747	177522.0	6.16	294.0736	1.02	9.5	$C_{15}H_{11}O_2N_1F_3$
296.0913	271668.0	9.42	296.0917	-0.48	11.5	$C_{17}H_{14}O_4N_1$
306.0763	265476.0	9.21	306.0761	0.19	13.5	$C_{18}H_{12}O_4N_1$
307.0832	167397.0	5.81	307.0839	-0.74	13.0	$C_{18}H_{13}O_4N_1$
308.0905	359681.0	12.48	308.0893	1.16	9.5	$C_{16}H_{13}O_2N_1F_3$
321.0999	194925.0	6.76	321.0996	0.38	13.0	$C_{19}H_{15}O_4N_1$
322.1082	457496.0	15.87	322.1085	-0.38	8.5	$C_{16}H_{17}O_5N_1F_1$
324.0871	321617.0	11.16	324.0866	0.46	12.5	$C_{18}H_{14}O_5N_1$
335.0753	180662.0	6.27	335.0752	0.03	15.0	$C_{20}H_{11}O_2N_1F_2$
336.0855	264214.0	9.16	336.0866	-1.11	13.5	$C_{19}H_{14}O_5N_1$
337.0919	167602.0	5.81	337.0920	-0.12	10.0	$C_{17}H_{14}O_3N_1F_3$
350.1009	1618536.0	56.14	350.0999	1.06	10.5	$C_{18}H_{15}O_3N_1F_3$
351.1075	1896456.0	65.78	351.1077	-0.16	10.0	$C_{18}H_{16}O_3N_1F_3$
352.1141	835108.0	28.97	352.1144	-0.22	13.5	$C_{21}H_{16}O_2N_1F_2$
366.9798	201410.0	6.99	366.9826	-2.78	25.5	$C_{25}O_3F_1$
367.2203	1899567.0	65.89	367.2232	-2.84	10.5	$C_{25}H_{29}F_2$
368.2240	521708.0	18.10	368.2243	-0.34	1.5	$C_{17}H_{32}O_5N_1F_2$
382.1274	633668.0	21.98	382.1285	-1.12	12.5	$C_{21}H_{20}O_6N_1$
449.2970	720477.0	24.99	449.2959	1.10	-1.0	$C_{20}H_{42}O_6N_1F_3$
450.2996	240266.0	8.33	450.3003	-0.69	10.5	$C_{29}H_{40}O_3N_1$
510.1526	2883026.0	100.00	510.1523	0.29	16.5	$C_{28}H_{23}O_5N_1F_3$
541.1710	257170.0	8.92	541.1707	0.37	16.0	$C_{29}H_{26}O_6N_1F_3$

23j HREI-MS X-02-79A4 -c1 12/26/2018 3:59:19 PM

EIH-2018122410-ZWL_DLX-02-79A4 -c1#6 RT: 6.20
T: + c EI Full ms [49.50-800.50]
m/z= 48-803

m/z	Intensity	Relative	Theo. Mass	Delta (mmu)	RDB equiv.	Composition
52.0290	78528.0	1.12	52.0308	-1.74	3.0	C_4H_4
53.0393	84434.0	1.21	53.0386	0.77	2.5	C_4H_5
55.0198	133784.0	1.91	55.0178	1.95	2.5	$C_3H_3O_1$
55.0561	643517.0	9.20	55.0542	1.89	1.5	C_4H_7
56.0639	198162.0	2.83	56.0621	1.88	1.0	C_4H_8
57.0353	128843.0	1.84	57.0335	1.78	1.5	$C_3H_5O_1$
57.0716	905619.0	12.95	57.0699	1.73	0.5	C_4H_9
60.0218	416235.0	5.95	60.0206	1.24	1.0	$C_2H_4O_2$
61.0084	143695.0	2.05	61.0073	1.10	5.5	C_5H_1
62.0158	286046.0	4.09	62.0151	0.73	5.0	C_5H_2
63.0235	601358.0	8.60	63.0229	0.56	4.5	C_5H_3
64.0306	94228.0	1.35	64.0308	-0.13	4.0	C_5H_4
65.0387	217955.0	3.12	65.0386	0.12	3.5	C_5H_5
67.0543	151735.0	2.17	67.0542	0.11	2.5	C_5H_7
69.0698	309961.0	4.43	69.0699	-0.11	1.5	C_5H_9
70.0763	93877.0	1.34	70.0777	-1.36	1.0	C_5H_{10}
71.0478	85661.0	1.22	71.0491	-1.37	1.5	$C_4H_7O_1$
71.0841	254880.0	3.64	71.0855	-1.44	0.5	C_5H_{11}
73.0270	346360.0	4.95	73.0284	-1.37	1.5	$C_3H_5O_2$
74.0135	181673.0	2.60	74.0151	-1.57	6.0	C_6H_2
75.0219	208950.0	2.99	75.0229	-1.06	5.5	C_6H_3
75.0431	159687.0	2.28	75.0441	-0.94	0.5	$C_3H_7O_2$
76.0296	189333.0	2.71	76.0308	-1.16	5.0	C_6H_4
77.0374	276954.0	3.96	77.0386	-1.17	4.5	C_6H_5
79.0536	89082.0	1.27	79.0542	-0.64	3.5	C_6H_7
81.0697	159249.0	2.28	81.0699	-0.19	2.5	C_6H_9
82.0767	78411.0	1.12	82.0777	-0.99	2.0	C_6H_{10}
83.0850	260318.0	3.72	83.0855	-0.54	1.5	C_6H_{11}
85.1010	144338.0	2.06	85.1012	-0.20	0.5	C_6H_{13}
86.0149	118289.0	1.69	86.0151	-0.16	7.0	C_7H_2
87.0229	187433.0	2.68	87.0229	0.02	6.5	C_7H_3
87.0444	103934.0	1.49	87.0441	0.32	1.5	$C_4H_7O_2$
88.0309	202957.0	2.90	88.0308	0.17	6.0	C_7H_4
89.0387	463773.0	6.63	89.0386	0.16	5.5	C_7H_5
91.0541	168985.0	2.42	91.0542	-0.09	4.5	C_7H_7
92.0022	120569.0	1.72	92.0005	1.64	6.0	$C_4O_1N_2$
95.0864	84755.0	1.21	95.0855	0.84	2.5	C_7H_{11}
97.1019	112179.0	1.60	97.1012	0.71	1.5	C_7H_{13}
101.0361	86889.0	1.24	101.0346	1.50	2.5	$C_3H_5O_2N_2$
102.0417	91070.0	1.30	102.0424	-0.67	2.0	$C_3H_6O_2N_2$
105.0234	141269.0	2.02	105.0209	2.53	6.0	$C_6H_3O_1N_1$
113.0192	331391.0	4.74	113.0193	-0.05	-0.5	$H_5O_5N_2$
114.0174	85720.0	1.23	114.0186	-1.15	3.5	$C_4H_4O_3N_1$
114.0292	448570.0	6.41	114.0311	-1.93	3.0	$C_5H_6O_3$
115.0287	75721.0	1.08	115.0291	-0.38	7.5	$C_7H_3N_2$
115.0411	1322059.0	18.91	115.0417	-0.58	7.0	$C_8H_5N_1$
126.0492	95426.0	1.36	126.0464	2.84	8.0	$C_{10}H_6$
127.0572	98087.0	1.40	127.0542	2.94	7.5	$C_{10}H_7$
128.0523	99286.0	1.42	128.0495	2.82	7.5	$C_9H_6N_1$
129.0576	244765.0	3.50	129.0573	0.34	7.0	$C_9H_7N_1$
129.0928	89784.0	1.28	129.0910	1.82	1.5	$C_7H_{13}O_2$
130.0657	521748.0	7.46	130.0651	0.56	6.5	$C_9H_8N_1$
139.0540	148402.0	2.12	139.0542	-0.26	8.5	$C_{11}H_7$
140.0498	462866.0	6.62	140.0495	0.35	8.5	$C_{10}H_6N_1$
141.0574	1121265.0	16.03	141.0573	0.06	8.0	$C_{10}H_7N_1$

EIH-2018122410-ZWL_DLX-02-79A4 -c1#6 RT: 6.20
T: + c EI Full ms [49.50-800.50]
m/z= 48-803

m/z	Intensity	Relative	Theo. Mass	Delta (mmu)	RDB equiv.	Composition
142.0652	6992764.0	100.00	142.0651	0.09	7.5	$C_{10}H_8N_1$
143.0712	1547031.0	22.12	143.0703	0.93	2.5	$C_7H_{11}O_3$
144.0761	152320.0	2.18	144.0781	-1.97	2.0	$C_7H_{12}O_3$
149.0231	353991.0	5.06	149.0233	-0.22	6.5	$C_8H_5O_3$
151.0530	102677.0	1.47	151.0542	-1.23	9.5	$C_{12}H_7$
152.0499	121037.0	1.73	152.0495	0.45	9.5	$C_{11}H_6N_1$
152.0604	107764.0	1.54	152.0621	-1.61	9.0	$C_{12}H_8$
153.0563	107939.0	1.54	153.0573	-0.95	9.0	$C_{11}H_7N_1$
154.0643	181907.0	2.60	154.0651	-0.87	8.5	$C_{11}H_8N_1$
164.0487	104870.0	1.50	164.0495	-0.73	10.5	$C_{12}H_6N_1$
165.0556	80457.0	1.15	165.0546	0.99	5.5	$C_9H_9O_3$
166.0636	112851.0	1.61	166.0624	1.11	5.0	$C_9H_{10}O_3$
167.0721	112646.0	1.61	167.0730	-0.82	9.0	$C_{12}H_9N_1$
168.0789	75604.0	1.08	168.0781	0.81	4.0	$C_9H_{12}O_3$
177.0343	1052297.0	15.05	177.0335	0.82	11.5	$C_{13}H_5O_1$
177.0585	76130.0	1.09	177.0573	1.25	11.0	$C_{13}H_7N_1$
178.0372	155741.0	2.23	178.0373	-0.07	7.0	$C_8H_6O_3N_2$
178.0642	126066.0	1.80	178.0651	-0.90	10.5	$C_{13}H_8N_1$
179.0312	315312.0	4.51	179.0299	1.33	2.5	$C_4H_7O_6N_2$
182.0613	100250.0	1.43	182.0600	1.29	9.5	$C_{12}H_8O_1N_1$
190.0664	79376.0	1.14	190.0651	1.32	11.5	$C_{14}H_8N_1$
191.1411	93263.0	1.33	191.1430	-1.91	4.5	$C_{13}H_{19}O_1$
194.0590	88585.0	1.27	194.0600	-1.03	10.5	$C_{13}H_8O_1N_1$
195.0669	88000.0	1.26	195.0679	-1.00	10.0	$C_{13}H_9O_1N_1$
198.0535	137585.0	1.97	198.0523	1.25	5.0	$C_9H_{10}O_5$
198.0901	76072.0	1.09	198.0913	-1.20	8.5	$C_{13}H_{12}O_1N_1$
206.0587	91333.0	1.31	206.0600	-1.33	11.5	$C_{14}H_8O_1N_1$
207.0339	95251.0	1.36	207.0315	2.38	12.0	$C_{13}H_5O_2N_1$
209.0489	95719.0	1.37	209.0471	1.74	11.0	$C_{13}H_7O_2N_1$
210.0570	103846.0	1.49	210.0550	2.04	10.5	$C_{13}H_8O_2N_1$
211.0606	79054.0	1.13	211.0601	0.46	5.5	$C_{10}H_{11}O_5$
212.0693	76803.0	1.10	212.0706	-1.29	9.5	$C_{13}H_{10}O_2N_1$
222.0561	209009.0	2.99	222.0550	1.12	11.5	$C_{14}H_8O_2N_1$
223.0638	142467.0	2.04	223.0628	1.07	11.0	$C_{14}H_9O_2N_1$
224.0706	138257.0	1.98	224.0706	-0.00	10.5	$C_{14}H_{10}O_2N_1$
225.0795	113377.0	1.62	225.0784	1.11	10.0	$C_{14}H_{11}O_2N_1$
235.0641	98876.0	1.41	235.0628	1.32	12.0	$C_{15}H_9O_2N_1$
236.0692	129867.0	1.86	236.0679	1.25	7.0	$C_{12}H_{12}O_5$
238.0525	89170.0	1.28	238.0525	-0.10	16.0	$C_{17}H_6N_2$
240.0668	142000.0	2.03	240.0655	1.28	10.5	$C_{14}H_{10}O_3N_1$
241.0722	86802.0	1.24	241.0733	-1.11	10.0	$C_{14}H_{11}O_3N_1$
250.0507	105484.0	1.51	250.0499	0.84	12.5	$C_{15}H_8O_3N_1$
251.0584	196730.0	2.81	251.0577	0.69	12.0	$C_{15}H_9O_3N_1$
252.0641	182842.0	2.61	252.0628	1.28	7.0	$C_{12}H_{12}O_6$
253.0686	91070.0	1.30	253.0707	-2.04	6.5	$C_{12}H_{13}O_6$
254.0793	139690.0	2.00	254.0785	0.83	6.0	$C_{12}H_{14}O_6$
255.0539	157378.0	2.25	255.0526	1.25	11.0	$C_{14}H_9O_4N_1$
264.0684	183807.0	2.63	264.0682	0.17	17.0	$C_{19}H_8N_2$
265.0758	131036.0	1.87	265.0760	-0.22	16.5	$C_{19}H_9N_2$
266.0833	108670.0	1.55	266.0838	-0.57	16.0	$C_{19}H_{10}N_2$
267.0515	74727.0	1.07	267.0526	-1.13	12.0	$C_{15}H_9O_4N_1$
268.0617	157729.0	2.26	268.0604	1.28	11.5	$C_{15}H_{10}O_4N_1$
269.0675	188748.0	2.70	269.0683	-0.75	11.0	$C_{15}H_{11}O_4N_1$
270.0763	296688.0	4.24	270.0761	0.26	10.5	$C_{15}H_{12}O_4N_1$
278.0807	74406.0	1.06	278.0812	-0.50	12.5	$C_{17}H_{12}O_3N_1$

EIH-2018122410-ZWL_DLX-02-79A4 -c1#6 RT: 6.20
T: + c EI Full ms [49.50-800.50]
m/z= 48-803

m/z	Intensity	Relative	Theo. Mass	Delta (mmu)	RDB equiv.	Composition
279.0540	188222.0	2.69	279.0553	-1.33	17.5	$C_{19} H_{7} O_{1} N_{2}$
280.0614	173750.0	2.48	280.0604	0.99	12.5	$C_{16} H_{10} O_{4} N_{1}$
280.0979	92181.0	1.32	280.0968	1.10	11.5	$C_{17} H_{14} O_{3} N_{1}$
281.0675	131094.0	1.87	281.0683	-0.73	12.0	$C_{16} H_{11} O_{4} N_{1}$
282.0756	250963.0	3.59	282.0761	-0.52	11.5	$C_{16} H_{12} O_{4} N_{1}$
283.0835	152671.0	2.18	283.0839	-0.37	11.0	$C_{16} H_{13} O_{4} N_{1}$
284.0906	249939.0	3.57	284.0917	-1.13	10.5	$C_{16} H_{14} O_{4} N_{1}$
292.0597	149221.0	2.13	292.0604	-0.75	13.5	$C_{17} H_{10} O_{4} N_{1}$
294.0751	411323.0	5.88	294.0761	-0.97	12.5	$C_{17} H_{12} O_{4} N_{1}$
295.0825	161208.0	2.31	295.0839	-1.38	12.0	$C_{17} H_{13} O_{4} N_{1}$
297.0612	138901.0	1.99	297.0632	-1.93	12.0	$C_{16} H_{11} O_{5} N_{1}$
297.0981	104080.0	1.49	297.0996	-1.45	11.0	$C_{17} H_{15} O_{4} N_{1}$
298.0693	434800.0	6.22	298.0710	-1.66	11.5	$C_{16} H_{12} O_{5} N_{1}$
298.1048	205793.0	2.94	298.1074	-2.58	10.5	$C_{17} H_{16} O_{4} N_{1}$
299.0712	84053.0	1.20	299.0703	0.95	15.5	$C_{20} H_{11} O_{3}$
306.0748	107735.0	1.54	306.0761	-1.30	13.5	$C_{18} H_{12} O_{4} N_{1}$
307.0827	121622.0	1.74	307.0839	-1.17	13.0	$C_{18} H_{13} O_{4} N_{1}$
308.0896	313294.0	4.48	308.0917	-2.18	12.5	$C_{18} H_{14} O_{4} N_{1}$
309.0614	76130.0	1.09	309.0632	-1.77	13.0	$C_{17} H_{11} O_{5} N_{1}$
309.0932	96333.0	1.38	309.0910	2.17	16.5	$C_{22} H_{13} O_{2}$
310.0713	264382.0	3.78	310.0710	0.27	12.5	$C_{17} H_{12} O_{5} N_{1}$
310.1076	90398.0	1.29	310.1074	0.22	11.5	$C_{18} H_{16} O_{4} N_{1}$
311.0775	159717.0	2.28	311.0788	-1.34	12.0	$C_{17} H_{13} O_{5} N_{1}$
312.0865	451347.0	6.45	312.0866	-0.14	11.5	$C_{17} H_{14} O_{5} N_{1}$
313.0927	442343.0	6.33	313.0945	-1.79	11.0	$C_{17} H_{15} O_{5} N_{1}$
314.0940	99607.0	1.42	314.0937	0.28	15.0	$C_{21} H_{14} O_{3}$
321.0674	96128.0	1.37	321.0659	1.55	18.5	$C_{21} H_{9} O_{2} N_{2}$
321.0978	76657.0	1.10	321.0996	-1.74	13.0	$C_{19} H_{15} O_{4} N_{1}$
322.0719	444448.0	6.36	322.0710	0.92	13.5	$C_{18} H_{12} O_{5} N_{1}$
322.1069	99549.0	1.42	322.1074	-0.51	12.5	$C_{19} H_{16} O_{4} N_{1}$
323.0789	183281.0	2.62	323.0788	0.03	13.0	$C_{18} H_{13} O_{5} N_{1}$
324.0869	348904.0	4.99	324.0866	0.29	12.5	$C_{18} H_{14} O_{5} N_{1}$
325.0944	284555.0	4.07	325.0945	-0.11	12.0	$C_{18} H_{15} O_{5} N_{1}$
326.1018	309172.0	4.42	326.1023	-0.54	11.5	$C_{18} H_{16} O_{5} N_{1}$
327.1094	319112.0	4.56	327.1101	-0.76	11.0	$C_{18} H_{17} O_{5} N_{1}$
336.0846	321422.0	4.60	336.0866	-2.03	13.5	$C_{19} H_{14} O_{5} N_{1}$
337.0919	487513.0	6.97	337.0945	-2.54	13.0	$C_{19} H_{15} O_{5} N_{1}$
338.0995	393723.0	5.63	338.1023	-2.77	12.5	$C_{19} H_{16} O_{5} N_{1}$
339.1067	238830.0	3.42	339.1043	2.40	21.0	$C_{26} H_{13} N_{1}$
340.1143	96771.0	1.38	340.1121	2.27	20.5	$C_{26} H_{14} N_{1}$
350.1021	220411.0	3.15	350.1023	-0.15	13.5	$C_{20} H_{16} O_{5} N_{1}$
351.1102	346740.0	4.96	351.1101	0.03	13.0	$C_{20} H_{17} O_{5} N_{1}$
352.1187	1081533.0	15.47	352.1179	0.74	12.5	$C_{20} H_{18} O_{5} N_{1}$
353.1210	240906.0	3.45	353.1199	1.08	21.0	$C_{27} H_{15} N_{1}$
354.0998	105893.0	1.51	354.0999	-0.09	17.0	$C_{22} H_{14} O_{3} N_{2}$
367.1089	92620.0	1.32	367.1077	1.17	17.5	$C_{23} H_{15} O_{3} N_{2}$
368.1131	162786.0	2.33	368.1129	0.21	12.5	$C_{20} H_{18} O_{6} N_{1}$
369.1216	211494.0	3.02	369.1207	0.95	12.0	$C_{20} H_{19} O_{6} N_{1}$
382.1285	308529.0	4.41	382.1285	0.01	12.5	$C_{21} H_{20} O_{6} N_{1}$
383.1365	141912.0	2.03	383.1363	0.17	12.0	$C_{21} H_{21} O_{6} N_{1}$
383.1965	204068.0	2.92	383.1965	0.01	10.5	$C_{22} H_{27} O_{4} N_{2}$
431.2884	199887.0	2.86	431.2904	-2.01	7.5	$C_{25} H_{39} O_{4} N_{2}$
433.3034	449067.0	6.42	433.3061	-2.73	6.5	$C_{25} H_{41} O_{4} N_{2}$
434.3058	130334.0	1.86	434.3054	0.45	10.5	$C_{29} H_{40} O_{2} N_{1}$
435.1354	76978.0	1.10	435.1339	1.52	19.5	$C_{27} H_{19} O_{4} N_{2}$

EIH-2018122410-ZWL_DLX-02-79A4 -c1#6 RT: 6.20
T: + c EI Full ms [49.50-800.50]
m/z= 48-803

m/z	Intensity	Relative	Theo. Mass	Delta (mmu)	RDB equiv.	Composition
437.1185	93555.0	1.34	437.1172	1.27	23.5	$C_{31}H_{17}O_3$
451.1533	103759.0	1.48	451.1540	-0.65	18.5	$C_{29}H_{23}O_5$
453.1426	109664.0	1.57	453.1445	-1.88	18.5	$C_{27}H_{21}O_5N_2$
454.1518	327298.0	4.68	454.1523	-0.49	18.0	$C_{27}H_{22}O_5N_2$
455.1543	97736.0	1.40	455.1516	2.69	22.0	$C_{31}H_{21}O_3N_1$
468.1690	336976.0	4.82	468.1680	1.01	18.0	$C_{28}H_{24}O_5N_2$
478.1534	93672.0	1.34	478.1523	1.09	20.0	$C_{29}H_{22}O_5N_2$
493.1782	829956.0	11.87	493.1758	2.44	19.5	$C_{30}H_{25}O_5N_2$
510.1756	346477.0	4.95	510.1785	-2.95	19.0	$C_{30}H_{26}O_6N_2$
524.1951	76306.0	1.09	524.1942	0.94	19.0	$C_{31}H_{28}O_6N_2$

23k HREI-MS _DLX-02-52A1-0 -c1 2/26/2019 2:27:31 PM

EIH-2019022507-ZWL-DLX_DLX-02-52A1-0 -c1#4 RT: 6.15
T: + c EI Full ms [49.50-800.50]
m/z= 48-803

m/z	Intensity	Relative	Theo. Mass	Delta (mmu)	RDB equiv.	Composition
50.0229	3690277.0	31.93	50.0237	-0.75	-0.5	$H_4 O_2 N_1$
51.9956	1323554.0	11.45	51.9944	1.24	4.0	$C_3 O_1$
60.0226	1011573.0	8.75	60.0206	1.98	1.0	$C_2 H_4 O_2$
61.0299	127841.0	1.11	61.0284	1.51	0.5	$C_2 H_5 O_2$
62.0160	204055.0	1.77	62.0151	0.89	5.0	$C_5 H_2$
63.0237	529234.0	4.58	63.0229	0.74	4.5	$C_5 H_3$
65.0386	445720.0	3.86	65.0386	0.00	3.5	$C_5 H_5$
67.0541	640197.0	5.54	67.0542	-0.13	2.5	$C_5 H_7$
68.0619	396663.0	3.43	68.0621	-0.13	2.0	$C_5 H_8$
69.0698	1607794.0	13.91	69.0699	-0.04	1.5	$C_5 H_9$
70.0762	788070.0	6.82	70.0777	-1.52	1.0	$C_5 H_{10}$
71.0475	205573.0	1.78	71.0491	-1.66	1.5	$C_4 H_7 O_1$
71.0840	1276074.0	11.04	71.0855	-1.54	0.5	$C_5 H_{11}$
73.0275	941024.0	8.14	73.0284	-0.93	1.5	$C_3 H_5 O_2$
74.0142	208785.0	1.81	74.0151	-0.95	6.0	$C_6 H_2$
74.0350	260120.0	2.25	74.0362	-1.25	1.0	$C_3 H_6 O_2$
75.0229	206624.0	1.79	75.0229	-0.07	5.5	$C_6 H_3$
75.0443	116452.0	1.01	75.0441	0.23	0.5	$C_3 H_7 O_2$
76.0304	218947.0	1.89	76.0308	-0.35	5.0	$C_6 H_4$
77.0386	2017306.0	17.46	77.0386	0.04	4.5	$C_6 H_5$
78.0459	676582.0	5.85	78.0464	-0.50	4.0	$C_6 H_6$
79.0547	1987638.0	17.20	79.0542	0.45	3.5	$C_6 H_7$
80.0604	173394.0	1.50	80.0621	-1.67	3.0	$C_6 H_8$
81.0710	660346.0	5.71	81.0699	1.15	2.5	$C_6 H_9$
82.0785	501260.0	4.34	82.0777	0.85	2.0	$C_6 H_{10}$
83.0494	193601.0	1.68	83.0491	0.26	2.5	$C_5 H_7 O_1$
83.0861	1271518.0	11.00	83.0855	0.60	1.5	$C_6 H_{11}$
84.0574	153362.0	1.33	84.0570	0.41	2.0	$C_5 H_8 O_1$
84.0929	370791.0	3.21	84.0934	-0.44	1.0	$C_6 H_{12}$
85.1018	763717.0	6.61	85.1012	0.60	0.5	$C_6 H_{13}$
87.0443	257025.0	2.22	87.0441	0.22	1.5	$C_4 H_7 O_2$
89.0388	283305.0	2.45	89.0386	0.21	5.5	$C_7 H_5$
91.0543	1125982.0	9.74	91.0542	0.03	4.5	$C_7 H_7$
93.0691	182096.0	1.58	93.0699	-0.77	3.5	$C_7 H_9$
95.0876	469490.0	4.06	95.0855	2.08	2.5	$C_7 H_{11}$
96.0956	308769.0	2.67	96.0934	2.27	2.0	$C_7 H_{12}$
97.0670	129009.0	1.12	97.0648	2.16	2.5	$C_6 H_9 O_1$
97.1035	980094.0	8.48	97.1012	2.34	1.5	$C_7 H_{13}$
98.0749	182096.0	1.58	98.0726	2.29	2.0	$C_6 H_{10} O_1$
98.1104	217195.0	1.88	98.1090	1.35	1.0	$C_7 H_{14}$
99.1176	231795.0	2.01	99.1168	0.81	0.5	$C_7 H_{15}$
103.0245	1795614.0	15.54	103.0264	-1.94	2.0	$C_3 H_5 O_3 N_1$
104.9810	127140.0	1.10	104.9818	-0.85	2.5	$C_2 H_1 O_5$
105.0178	11556096.0	100.00	105.0182	-0.42	1.5	$C_3 H_5 O_4$
106.0121	1199801.0	10.38	106.0135	-1.39	1.5	$C_2 H_4 O_4 N_1$
107.0134	143434.0	1.24	107.0128	0.64	5.5	$C_6 H_3 O_2$
109.0156	273377.0	2.37	109.0158	-0.21	5.0	$C_5 H_3 O_2 N_1$
110.0186	132396.0	1.15	110.0210	-2.34	0.0	$C_2 H_6 O_5$
111.0245	453780.0	3.93	111.0229	1.56	8.5	$C_9 H_3$
112.0299	157275.0	1.36	112.0308	-0.86	8.0	$C_9 H_4$
125.0327	276239.0	2.39	125.0319	0.85	0.0	$C_2 H_7 O_5 N_1$
141.0419	189746.0	1.64	141.0420	-0.14	4.0	$C_6 H_7 O_3 N_1$
142.0349	646797.0	5.60	142.0346	0.33	-0.5	$C_2 H_8 O_6 N_1$
149.0234	358468.0	3.10	149.0233	0.08	6.5	$C_8 H_5 O_3$
191.1428	227182.0	1.97	191.1430	-0.27	4.5	$C_{13} H_{19} O_1$

EIH-2019022507-ZWL-DLX_DLX-02-52A1-0 -c1#4 RT: 6.15
T: + c EI Full ms [49.50-800.50]
m/z= 48-803

m/z	Intensity	Relative	Theo. Mass	Delta (mmu)	RDB equiv.	Composition
207.0321	268413.0	2.32	207.0315	0.65	12.0	$C_{13}H_{5}O_{2}N_{1}$
264.0664	146587.0	1.27	264.0655	0.86	12.5	$C_{16}H_{10}O_{3}N_{1}$
282.0739	138528.0	1.20	282.0761	-2.18	11.5	$C_{16}H_{12}O_{4}N_{1}$
292.0589	146120.0	1.26	292.0604	-1.54	13.5	$C_{17}H_{10}O_{4}N_{1}$
294.0736	146529.0	1.27	294.0761	-2.52	12.5	$C_{17}H_{12}O_{4}N_{1}$
297.0993	114291.0	0.99	297.0996	-0.25	11.0	$C_{17}H_{15}O_{4}N_{1}$
306.0777	110904.0	0.96	306.0761	1.57	13.5	$C_{18}H_{12}O_{4}N_{1}$
308.0922	284181.0	2.46	308.0917	0.42	12.5	$C_{18}H_{14}O_{4}N_{1}$
322.1079	166678.0	1.44	322.1074	0.49	12.5	$C_{19}H_{16}O_{4}N_{1}$
324.0882	205281.0	1.78	324.0866	1.53	12.5	$C_{18}H_{14}O_{5}N_{1}$
336.0873	337560.0	2.92	336.0866	0.62	13.5	$C_{19}H_{14}O_{5}N_{1}$
337.0947	317938.0	2.75	337.0945	0.19	13.0	$C_{19}H_{15}O_{5}N_{1}$
338.1028	111138.0	0.96	338.1023	0.54	12.5	$C_{19}H_{16}O_{5}N_{1}$
350.1033	509495.0	4.41	350.1023	0.96	13.5	$C_{20}H_{16}O_{5}N_{1}$
351.1110	588220.0	5.09	351.1101	0.83	13.0	$C_{20}H_{17}O_{5}N_{1}$
352.1183	1222753.0	10.58	352.1179	0.33	12.5	$C_{20}H_{18}O_{5}N_{1}$
353.1234	315835.0	2.73	353.1258	-2.33	12.0	$C_{20}H_{19}O_{5}N_{1}$
367.1016	147171.0	1.27	367.0992	2.40	22.0	$C_{27}H_{13}O_{1}N_{1}$
382.1287	543718.0	4.71	382.1285	0.21	12.5	$C_{21}H_{20}O_{6}N_{1}$
383.1384	194185.0	1.68	383.1363	2.05	12.0	$C_{21}H_{21}O_{6}N_{1}$
456.1830	1947925.0	16.86	456.1805	2.48	16.5	$C_{28}H_{26}O_{5}N_{1}$
457.1872	609537.0	5.27	457.1884	-1.17	16.0	$C_{28}H_{27}O_{5}N_{1}$
487.1989	198915.0	1.72	487.1989	-0.01	16.0	$C_{29}H_{29}O_{6}N_{1}$

23l HREI-MS X_DLX-02-60A2-c1 12/11/2018 2:19:18 PM

EIH-2018120607-ZWL-DLX_DLX-02-60A2-c1#8 RT: 6.19
T: + c EI Full ms [49.50-800.50]
m/z= 48-803

m/z	Intensity	Relative	Theo. Mass	Delta (mmu)	RDB equiv.	Composition
51.0224	268587.0	3.01	51.0229	-0.52	3.5	C_4H_3
51.9918	551935.0	6.19	51.9944	-2.61	4.0	C_3O_1
63.0237	453475.0	5.09	63.0229	0.81	4.5	C_5H_3
75.0221	208395.0	2.34	75.0229	-0.81	5.5	C_6H_3
76.0297	301514.0	3.38	76.0308	-1.02	5.0	C_6H_4
77.0377	210215.0	2.36	77.0386	-0.92	4.5	C_6H_5
82.0306	365139.0	4.10	82.0287	1.90	3.5	$C_4H_4O_1N_1$
83.0378	228792.0	2.57	83.0366	1.27	3.0	$C_4H_5O_1N_1$
89.0380	276276.0	3.10	89.0386	-0.54	5.5	C_7H_5
115.0536	384157.0	4.31	115.0542	-0.66	6.5	C_9H_7
128.0622	202232.0	2.27	128.0621	0.14	7.0	$C_{10}H_8$
139.0540	344772.0	3.87	139.0542	-0.23	8.5	$C_{11}H_7$
151.0541	317215.0	3.56	151.0542	-0.16	9.5	$C_{12}H_7$
152.0622	1426135.0	16.00	152.0621	0.11	9.0	$C_{12}H_8$
163.0532	259166.0	2.91	163.0542	-1.04	10.5	$C_{13}H_7$
165.0688	2230400.0	25.02	165.0699	-1.08	9.5	$C_{13}H_9$
167.0849	8913152.0	100.00	167.0855	-0.68	8.5	$C_{13}H_{11}$
169.0941	185240.0	2.08	169.0945	-0.37	-1.0	$C_5H_{15}O_5N_1$
252.0653	188644.0	2.12	252.0655	-0.22	11.5	$C_{15}H_{10}O_3N_1$
282.0750	321412.0	3.61	282.0761	-1.10	11.5	$C_{16}H_{12}O_4N_1$
292.0619	204463.0	2.29	292.0604	1.46	13.5	$C_{17}H_{10}O_4N_1$
293.0667	248924.0	2.79	293.0683	-1.54	13.0	$C_{17}H_{11}O_4N_1$
294.0762	413269.0	4.64	294.0761	0.10	12.5	$C_{17}H_{12}O_4N_1$
296.0917	194895.0	2.19	296.0917	-0.03	11.5	$C_{17}H_{14}O_4N_1$
297.0999	197683.0	2.22	297.0996	0.29	11.0	$C_{17}H_{15}O_4N_1$
308.0909	528017.0	5.92	308.0917	-0.87	12.5	$C_{18}H_{14}O_4N_1$
322.0710	296525.0	3.33	322.0710	-0.02	13.5	$C_{18}H_{12}O_5N_1$
324.0859	319651.0	3.59	324.0866	-0.79	12.5	$C_{18}H_{14}O_5N_1$
336.0847	538494.0	6.04	336.0866	-1.92	13.5	$C_{19}H_{14}O_5N_1$
337.0928	630264.0	7.07	337.0945	-1.63	13.0	$C_{19}H_{15}O_5N_1$
338.0994	244551.0	2.74	338.1023	-2.91	12.5	$C_{19}H_{16}O_5N_1$
350.1028	581400.0	6.52	350.1023	0.55	13.5	$C_{20}H_{16}O_5N_1$
351.1098	755548.0	8.48	351.1101	-0.28	13.0	$C_{20}H_{17}O_5N_1$
352.1172	2671901.0	29.98	352.1179	-0.78	12.5	$C_{20}H_{18}O_5N_1$
353.1208	636162.0	7.14	353.1199	0.87	21.0	$C_{27}H_{15}N_1$
380.1118	256437.0	2.88	380.1129	-1.03	13.5	$C_{21}H_{18}O_6N_1$
382.1277	824573.0	9.25	382.1285	-0.77	12.5	$C_{21}H_{20}O_6N_1$
383.1328	350642.0	3.93	383.1305	2.33	21.0	$C_{28}H_{17}O_1N_1$
518.1937	2223972.0	24.95	518.1962	-2.48	20.5	$C_{33}H_{28}O_5N_1$
549.2146	256994.0	2.88	549.2146	0.03	20.0	$C_{34}H_{31}O_6N_1$

23m HREI-MS LX-02-62A2 -c1 12/11/2018 3:45:30 PM

EIH-2018120716-ZWL-DLX_DLX-02-62A2 -c1#10 RT: 6.81
T: + c EI Full ms [49.50-800.50]
m/z= 48-803

m/z	Intensity	Relative	Theo. Mass	Delta (mmu)	RDB equiv.	Composition
50.0034	276041.0	5.02	50.0025	0.86	4.5	$C_3 N_1$
52.0282	81791.0	1.49	52.0308	-2.50	3.0	$C_4 H_4$
53.0019	46192.0	0.84	53.0022	-0.26	3.5	$C_3 H_1 O_1$
53.0384	121204.0	2.20	53.0386	-0.21	2.5	$C_4 H_5$
55.0188	76831.0	1.40	55.0178	0.95	2.5	$C_3 H_3 O_1$
55.0550	388353.0	7.06	55.0542	0.78	1.5	$C_4 H_7$
56.0632	81497.0	1.48	56.0621	1.15	1.0	$C_4 H_8$
57.0236	32986.0	0.60	57.0209	2.71	2.0	$C_2 H_3 O_1 N_1$
57.0709	321177.0	5.84	57.0699	1.00	0.5	$C_4 H_9$
58.0307	31812.0	0.58	58.0287	2.00	1.5	$C_2 H_4 O_1 N_1$
58.0418	48159.0	0.88	58.0413	0.53	1.0	$C_3 H_6 O_1$
60.0001	40264.0	0.73	59.9995	0.62	6.0	C_5
60.0209	167954.0	3.05	60.0206	0.29	1.0	$C_2 H_4 O_2$
61.0077	131945.0	2.40	61.0073	0.39	5.5	$C_5 H_1$
62.0151	184096.0	3.35	62.0151	-0.04	5.0	$C_5 H_2$
63.0228	472903.0	8.60	63.0229	-0.12	4.5	$C_5 H_3$
64.0308	194103.0	3.53	64.0308	0.01	4.0	$C_5 H_4$
65.0388	445874.0	8.11	65.0386	0.21	3.5	$C_5 H_5$
66.0461	45018.0	0.82	66.0464	-0.29	3.0	$C_5 H_6$
67.0541	56434.0	1.03	67.0542	-0.08	2.5	$C_5 H_7$
69.0701	103889.0	1.89	69.0699	0.26	1.5	$C_5 H_9$
70.0766	37359.0	0.68	70.0777	-1.12	1.0	$C_5 H_{10}$
71.0482	33778.0	0.61	71.0491	-0.92	1.5	$C_4 H_7 O_1$
71.0842	67939.0	1.24	71.0855	-1.30	0.5	$C_5 H_{11}$
73.0061	41849.0	0.76	73.0073	-1.14	6.5	$C_6 H_1$
73.0278	131036.0	2.38	73.0284	-0.64	1.5	$C_3 H_5 O_2$
74.0142	160177.0	2.91	74.0151	-0.86	6.0	$C_6 H_2$
75.0224	180926.0	3.29	75.0229	-0.49	5.5	$C_6 H_3$
75.0436	58724.0	1.07	75.0441	-0.42	0.5	$C_3 H_7 O_2$
76.0304	171417.0	3.12	76.0308	-0.31	5.0	$C_6 H_4$
77.0384	414590.0	7.54	77.0386	-0.13	4.5	$C_6 H_5$
78.0460	144447.0	2.63	78.0464	-0.37	4.0	$C_6 H_6$
79.0180	31959.0	0.58	79.0178	0.12	4.5	$C_5 H_3 O_1$
79.0545	142921.0	2.60	79.0542	0.28	3.5	$C_6 H_7$
81.0698	45429.0	0.83	81.0699	-0.04	2.5	$C_6 H_9$
83.0860	65004.0	1.18	83.0855	0.44	1.5	$C_6 H_{11}$
85.0075	40499.0	0.74	85.0073	0.20	7.5	$C_7 H_1$
85.0294	39413.0	0.72	85.0284	0.95	2.5	$C_4 H_5 O_2$
85.1016	44255.0	0.80	85.1012	0.42	0.5	$C_6 H_{13}$
86.0156	71372.0	1.30	86.0151	0.45	7.0	$C_7 H_2$
87.0234	107910.0	1.96	87.0229	0.43	6.5	$C_7 H_3$
87.0449	36244.0	0.66	87.0441	0.80	1.5	$C_4 H_7 O_2$
88.0301	69817.0	1.27	88.0308	-0.60	6.0	$C_7 H_4$
89.0384	281089.0	5.11	89.0386	-0.13	5.5	$C_7 H_5$
90.0350	37770.0	0.69	90.0338	1.15	5.5	$C_6 H_4 N_1$
90.0441	52766.0	0.96	90.0464	-2.30	5.0	$C_7 H_6$
91.0543	882064.0	16.04	91.0542	0.04	4.5	$C_7 H_7$
92.0256	37388.0	0.68	92.0257	-0.10	5.0	$C_6 H_4 O_1$
93.0703	119502.0	2.17	93.0699	0.43	3.5	$C_7 H_9$
97.1015	34365.0	0.63	97.1012	0.32	1.5	$C_7 H_{13}$
98.0155	47102.0	0.86	98.0151	0.40	8.0	$C_8 H_2$
99.0231	40352.0	0.73	99.0229	0.15	7.5	$C_8 H_3$
101.0388	64945.0	1.18	101.0386	0.19	6.5	$C_8 H_5$
102.0463	105180.0	1.91	102.0464	-0.12	6.0	$C_8 H_6$
103.0550	159502.0	2.90	103.0542	0.76	5.5	$C_8 H_7$

EIH-2018120716-ZWL-DLX_DLX-02-62A2 -c1#10 RT: 6.81
T: + c EI Full ms [49.50-800.50]
m/z= 48-803

m/z	Intensity	Relative	Theo. Mass	Delta (mmu)	RDB equiv.	Composition
104.0626	248983.0	4.53	104.0621	0.53	5.0	C_8H_8
105.0340	85664.0	1.56	105.0335	0.48	5.5	$C_7H_5O_1$
105.0708	1135537.0	20.65	105.0699	0.88	4.5	C_8H_9
107.0863	183010.0	3.33	107.0855	0.78	3.5	C_8H_{11}
111.0257	42406.0	0.77	111.0229	2.77	8.5	C_9H_3
112.0320	33133.0	0.60	112.0308	1.24	8.0	C_9H_4
113.0385	79560.0	1.45	113.0386	-0.12	7.5	C_9H_5
114.0453	55848.0	1.02	114.0464	-1.14	7.0	C_9H_6
115.0538	758658.0	13.80	115.0542	-0.47	6.5	C_9H_7
116.0607	210097.0	3.82	116.0621	-1.34	6.0	C_9H_8
117.0696	1921284.0	34.95	117.0699	-0.32	5.5	C_9H_9
119.0854	823311.0	14.98	119.0855	-0.17	4.5	C_9H_{11}
125.0397	52795.0	0.96	125.0386	1.12	8.5	$C_{10}H_5$
126.0345	55877.0	1.02	126.0338	0.64	8.5	$C_9H_4N_1$
126.0461	93031.0	1.69	126.0464	-0.30	8.0	$C_{10}H_6$
127.0532	143332.0	2.61	127.0542	-0.98	7.5	$C_{10}H_7$
128.0621	141278.0	2.57	128.0621	0.10	7.0	$C_{10}H_8$
129.0696	126223.0	2.30	129.0699	-0.28	6.5	$C_{10}H_9$
130.0757	32106.0	0.58	130.0777	-1.99	6.0	$C_{10}H_{10}$
131.0478	46339.0	0.84	131.0491	-1.32	6.5	$C_9H_7O_1$
131.0848	334090.0	6.08	131.0855	-0.68	5.5	$C_{10}H_{11}$
132.0932	1667722.0	30.33	132.0934	-0.19	5.0	$C_{10}H_{12}$
133.0993	504598.0	9.18	133.1012	-1.86	4.5	$C_{10}H_{13}$
138.0448	55114.0	1.00	138.0464	-1.64	9.0	$C_{11}H_6$
139.0535	166634.0	3.03	139.0542	-0.77	8.5	$C_{11}H_7$
140.0503	112253.0	2.04	140.0495	0.80	8.5	$C_{10}H_6N_1$
141.0581	52267.0	0.95	141.0573	0.77	8.0	$C_{10}H_7N_1$
141.0684	38855.0	0.71	141.0699	-1.48	7.5	$C_{11}H_9$
142.0632	32223.0	0.59	142.0624	0.78	3.0	$C_7H_{10}O_3$
145.1008	117594.0	2.14	145.1012	-0.34	5.5	$C_{11}H_{13}$
147.0808	57080.0	1.04	147.0804	0.32	5.5	$C_{10}H_{11}O_1$
147.1166	5497877.0	100.00	147.1168	-0.21	4.5	$C_{11}H_{15}$
149.0245	33544.0	0.61	149.0233	1.14	6.5	$C_8H_5O_3$
150.0480	56611.0	1.03	150.0464	1.56	10.0	$C_{12}H_6$
151.0543	82172.0	1.49	151.0542	0.12	9.5	$C_{12}H_7$
152.0592	98489.0	1.79	152.0621	-2.81	9.0	$C_{12}H_8$
153.0578	77389.0	1.41	153.0573	0.48	9.0	$C_{11}H_7N_1$
154.0653	86134.0	1.57	154.0651	0.16	8.5	$C_{11}H_8N_1$
156.0426	32634.0	0.59	156.0417	0.87	4.0	$C_7H_8O_4$
163.0519	49831.0	0.91	163.0542	-2.31	10.5	$C_{13}H_7$
164.0482	86310.0	1.57	164.0495	-1.31	10.5	$C_{12}H_6N_1$
165.0586	67821.0	1.23	165.0573	1.31	10.0	$C_{12}H_7N_1$
165.0678	64916.0	1.18	165.0699	-2.06	9.5	$C_{13}H_9$
166.0654	118035.0	2.15	166.0651	0.30	9.5	$C_{12}H_8N_1$
167.0700	101013.0	1.84	167.0703	-0.31	4.5	$C_9H_{11}O_3$
168.0467	42788.0	0.78	168.0444	2.35	9.5	$C_{11}H_6O_1N_1$
169.0548	61130.0	1.11	169.0522	2.59	9.0	$C_{11}H_7O_1N_1$
170.0615	54938.0	1.00	170.0600	1.48	8.5	$C_{11}H_8O_1N_1$
176.0503	31841.0	0.58	176.0495	0.81	11.5	$C_{13}H_6N_1$
177.0574	66207.0	1.20	177.0573	0.10	11.0	$C_{13}H_7N_1$
178.0657	99399.0	1.81	178.0651	0.62	10.5	$C_{13}H_8N_1$
179.0699	37711.0	0.69	179.0703	-0.39	5.5	$C_{10}H_{11}O_3$
179.0804	35451.0	0.64	179.0788	1.58	1.0	$C_6H_{13}O_5N_1$
180.0448	38034.0	0.69	180.0444	0.39	10.5	$C_{12}H_6O_1N_1$
180.0786	47660.0	0.87	180.0781	0.48	5.0	$C_{10}H_{12}O_3$

EIH-2018120716-ZWL-DLX_DLX-02-62A2 -c1#10 RT: 6.81
T: + c EI Full ms [49.50-800.50]
m/z= 48-803

m/z	Intensity	Relative	Theo. Mass	Delta (mmu)	RDB equiv.	Composition
181.0566	45517.0	0.83	181.0581	-1.52	1.0	$C_5H_{11}O_6N_1$
182.0597	82260.0	1.50	182.0600	-0.38	9.5	$C_{12}H_8O_1N_1$
183.0669	55671.0	1.01	183.0679	-0.96	9.0	$C_{12}H_9O_1N_1$
184.0761	33661.0	0.61	184.0757	0.44	8.5	$C_{12}H_{10}O_1N_1$
184.0776	31900.0	0.58	184.0757	1.93	8.5	$C_{12}H_{10}O_1N_1$
190.0659	56288.0	1.02	190.0651	0.78	11.5	$C_{14}H_8N_1$
191.0737	46867.0	0.85	191.0730	0.79	11.0	$C_{14}H_9N_1$
191.1429	32252.0	0.59	191.1430	-0.11	4.5	$C_{13}H_{19}O_1$
193.0514	36537.0	0.66	193.0522	-0.77	11.0	$C_{13}H_7O_1N_1$
194.0604	100045.0	1.82	194.0600	0.31	10.5	$C_{13}H_8O_1N_1$
195.0672	76391.0	1.39	195.0679	-0.71	10.0	$C_{13}H_9O_1N_1$
196.0747	53881.0	0.98	196.0757	-1.01	9.5	$C_{13}H_{10}O_1N_1$
198.0551	107763.0	1.96	198.0550	0.10	9.5	$C_{12}H_8O_2N_1$
206.0601	98753.0	1.80	206.0600	0.04	11.5	$C_{14}H_8O_1N_1$
207.0672	71255.0	1.30	207.0679	-0.69	11.0	$C_{14}H_9O_1N_1$
208.0761	65650.0	1.19	208.0757	0.39	10.5	$C_{14}H_{10}O_1N_1$
209.0474	38210.0	0.69	209.0471	0.28	11.0	$C_{13}H_7O_2N_1$
210.0539	81673.0	1.49	210.0550	-1.06	10.5	$C_{13}H_8O_2N_1$
211.0605	73133.0	1.33	211.0601	0.45	5.5	$C_{10}H_{11}O_5$
212.0688	94028.0	1.71	212.0679	0.92	5.0	$C_{10}H_{12}O_5$
213.0771	35539.0	0.65	213.0758	1.31	4.5	$C_{10}H_{13}O_5$
220.0764	34013.0	0.62	220.0757	0.70	11.5	$C_{15}H_{10}O_1N_1$
222.0557	172914.0	3.15	222.0550	0.74	11.5	$C_{14}H_8O_2N_1$
223.0624	119150.0	2.17	223.0628	-0.35	11.0	$C_{14}H_9O_2N_1$
224.0709	89538.0	1.63	224.0706	0.27	10.5	$C_{14}H_{10}O_2N_1$
225.0781	49597.0	0.90	225.0784	-0.30	10.0	$C_{14}H_{11}O_2N_1$
226.0500	58342.0	1.06	226.0499	0.08	10.5	$C_{13}H_8O_3N_1$
234.0544	51269.0	0.93	234.0550	-0.57	12.5	$C_{15}H_8O_2N_1$
235.0624	85782.0	1.56	235.0628	-0.38	12.0	$C_{15}H_9O_2N_1$
236.0702	120500.0	2.19	236.0706	-0.36	11.5	$C_{15}H_{10}O_2N_1$
237.0771	70052.0	1.27	237.0758	1.32	6.5	$C_{12}H_{13}O_5$
238.0505	89362.0	1.63	238.0499	0.62	11.5	$C_{14}H_8O_3N_1$
238.0864	60602.0	1.10	238.0863	0.13	10.5	$C_{15}H_{12}O_2N_1$
239.0567	75422.0	1.37	239.0577	-0.97	11.0	$C_{14}H_9O_3N_1$
240.0654	141718.0	2.58	240.0655	-0.09	10.5	$C_{14}H_{10}O_3N_1$
241.0731	89509.0	1.63	241.0733	-0.23	10.0	$C_{14}H_{11}O_3N_1$
248.0695	52766.0	0.96	248.0706	-1.09	12.5	$C_{16}H_{10}O_2N_1$
249.0774	32311.0	0.59	249.0784	-1.02	12.0	$C_{16}H_{11}O_2N_1$
250.0493	109935.0	2.00	250.0499	-0.57	12.5	$C_{15}H_8O_3N_1$
250.0837	36566.0	0.67	250.0836	0.15	7.0	$C_{13}H_{14}O_5$
251.0562	158769.0	2.89	251.0550	1.14	7.5	$C_{12}H_{11}O_6$
252.0651	149466.0	2.72	252.0655	-0.41	11.5	$C_{15}H_{10}O_3N_1$
253.0723	78680.0	1.43	253.0733	-1.07	11.0	$C_{15}H_{11}O_3N_1$
254.0458	40675.0	0.74	254.0448	0.97	11.5	$C_{14}H_8O_4N_1$
254.0813	118651.0	2.16	254.0812	0.10	10.5	$C_{15}H_{12}O_3N_1$
255.0507	56669.0	1.03	255.0526	-1.92	11.0	$C_{14}H_9O_4N_1$
262.0515	37535.0	0.68	262.0499	1.65	13.5	$C_{16}H_8O_3N_1$
263.0599	49949.0	0.91	263.0577	2.19	13.0	$C_{16}H_9O_3N_1$
264.0679	149818.0	2.73	264.0655	2.35	12.5	$C_{16}H_{10}O_3N_1$
266.0460	51827.0	0.94	266.0448	1.23	12.5	$C_{15}H_8O_4N_1$
266.0833	130096.0	2.37	266.0812	2.13	11.5	$C_{16}H_{12}O_3N_1$
267.0513	74014.0	1.35	267.0526	-1.36	12.0	$C_{15}H_9O_4N_1$
267.0865	65884.0	1.20	267.0863	0.18	6.5	$C_{13}H_{15}O_6$
268.0606	157448.0	2.86	268.0604	0.13	11.5	$C_{15}H_{10}O_4N_1$
268.0971	38445.0	0.70	268.0968	0.26	10.5	$C_{16}H_{14}O_3N_1$

EIH-2018120716-ZWL-DLX_DLX-02-62A2 -c1#10 RT: 6.81
T: + c EI Full ms [49.50-800.50]
m/z= 48-803

m/z	Intensity	Relative	Theo. Mass	Delta (mmu)	RDB equiv.	Composition
269.0681	216700.0	3.94	269.0683	-0.20	11.0	$C_{15} H_{11} O_4 N_1$
270.0756	103801.0	1.89	270.0761	-0.45	10.5	$C_{15} H_{12} O_4 N_1$
276.0654	35627.0	0.65	276.0655	-0.16	13.5	$C_{17} H_{10} O_3 N_1$
277.0720	44255.0	0.80	277.0733	-1.30	13.0	$C_{17} H_{11} O_3 N_1$
278.0815	63272.0	1.15	278.0812	0.33	12.5	$C_{17} H_{12} O_3 N_1$
278.2058	35921.0	0.65	278.2029	2.93	9.0	$C_{21} H_{26}$
279.0528	142745.0	2.60	279.0526	0.18	13.0	$C_{16} H_9 O_4 N_1$
279.0878	49039.0	0.89	279.0890	-1.20	12.0	$C_{17} H_{13} O_3 N_1$
280.0608	160324.0	2.92	280.0604	0.41	12.5	$C_{16} H_{10} O_4 N_1$
280.0965	73837.0	1.34	280.0968	-0.32	11.5	$C_{17} H_{14} O_3 N_1$
281.0676	98929.0	1.80	281.0683	-0.62	12.0	$C_{16} H_{11} O_4 N_1$
281.1036	55261.0	1.01	281.1046	-1.04	11.0	$C_{17} H_{15} O_3 N_1$
282.0746	208072.0	3.78	282.0761	-1.45	11.5	$C_{16} H_{12} O_4 N_1$
283.0815	148468.0	2.70	283.0839	-2.36	11.0	$C_{16} H_{13} O_4 N_1$
284.0908	140896.0	2.56	284.0917	-0.91	10.5	$C_{16} H_{14} O_4 N_1$
292.0596	123435.0	2.25	292.0604	-0.82	13.5	$C_{17} H_{10} O_4 N_1$
293.0666	145416.0	2.64	293.0683	-1.64	13.0	$C_{17} H_{11} O_4 N_1$
294.0756	283818.0	5.16	294.0761	-0.52	12.5	$C_{17} H_{12} O_4 N_1$
295.0815	115276.0	2.10	295.0839	-2.42	12.0	$C_{17} H_{13} O_4 N_1$
296.0552	50125.0	0.91	296.0553	-0.11	12.5	$C_{16} H_{10} O_5 N_1$
296.0893	104065.0	1.89	296.0917	-2.39	11.5	$C_{17} H_{14} O_4 N_1$
297.0629	133501.0	2.43	297.0632	-0.23	12.0	$C_{16} H_{11} O_5 N_1$
297.0981	46632.0	0.85	297.0996	-1.41	11.0	$C_{17} H_{15} O_4 N_1$
298.0701	118240.0	2.15	298.0710	-0.88	11.5	$C_{16} H_{12} O_5 N_1$
298.1067	214646.0	3.90	298.1074	-0.65	10.5	$C_{17} H_{16} O_4 N_1$
306.0770	74278.0	1.35	306.0761	0.87	13.5	$C_{18} H_{12} O_4 N_1$
307.0821	69934.0	1.27	307.0839	-1.83	13.0	$C_{18} H_{13} O_4 N_1$
308.0912	263568.0	4.79	308.0917	-0.48	12.5	$C_{18} H_{14} O_4 N_1$
309.0694	79061.0	1.44	309.0699	-0.49	21.5	$C_{25} H_9$
310.0696	205548.0	3.74	310.0710	-1.38	12.5	$C_{17} H_{12} O_5 N_1$
311.0764	102040.0	1.86	311.0788	-2.39	12.0	$C_{17} H_{13} O_5 N_1$
312.0849	370187.0	6.73	312.0866	-1.72	11.5	$C_{17} H_{14} O_5 N_1$
313.0890	135438.0	2.46	313.0886	0.43	20.0	$C_{24} H_{11} N_1$
320.0909	46427.0	0.84	320.0917	-0.86	13.5	$C_{19} H_{14} O_4 N_1$
321.0618	71548.0	1.30	321.0632	-1.42	14.0	$C_{18} H_{11} O_5 N_1$
321.0998	35363.0	0.64	321.0996	0.27	13.0	$C_{19} H_{15} O_4 N_1$
322.0692	220134.0	4.00	322.0710	-1.77	13.5	$C_{18} H_{12} O_5 N_1$
322.1061	61159.0	1.11	322.1074	-1.26	12.5	$C_{19} H_{16} O_4 N_1$
323.0773	146267.0	2.66	323.0788	-1.48	13.0	$C_{18} H_{13} O_5 N_1$
324.0840	201440.0	3.66	324.0866	-2.68	12.5	$C_{18} H_{14} O_5 N_1$
325.0902	98431.0	1.79	325.0886	1.57	21.0	$C_{25} H_{11} N_1$
326.1009	271668.0	4.94	326.1023	-1.36	11.5	$C_{18} H_{16} O_5 N_1$
327.1085	252269.0	4.59	327.1101	-1.63	11.0	$C_{18} H_{17} O_5 N_1$
328.1120	57784.0	1.05	328.1121	-0.05	19.5	$C_{25} H_{14} N_1$
335.0784	33896.0	0.62	335.0788	-0.39	14.0	$C_{19} H_{13} O_5 N_1$
336.0850	169510.0	3.08	336.0866	-1.63	13.5	$C_{19} H_{14} O_5 N_1$
337.0932	201352.0	3.66	337.0945	-1.30	13.0	$C_{19} H_{15} O_5 N_1$
338.1023	294764.0	5.36	338.1023	-0.05	12.5	$C_{19} H_{16} O_5 N_1$
339.1098	90184.0	1.64	339.1101	-0.30	12.0	$C_{19} H_{17} O_5 N_1$
340.1154	63126.0	1.15	340.1179	-2.59	11.5	$C_{19} H_{18} O_5 N_1$
350.1024	129803.0	2.36	350.1023	0.07	13.5	$C_{20} H_{16} O_5 N_1$
351.1092	174528.0	3.17	351.1101	-0.97	13.0	$C_{20} H_{17} O_5 N_1$
352.1181	289335.0	5.26	352.1179	0.14	12.5	$C_{20} H_{18} O_5 N_1$
353.1191	80881.0	1.47	353.1199	-0.84	21.0	$C_{27} H_{15} N_1$
354.0971	83522.0	1.52	354.0972	-0.07	12.5	$C_{19} H_{16} O_6 N_1$

EIH-2018120716-ZWL-DLX_DLX-02-62A2 -c1#10 RT: 6.81
T: + c EI Full ms [49.50-800.50]
m/z= 48-803

m/z	Intensity	Relative	Theo. Mass	Delta (mmu)	RDB equiv.	Composition
355.1038	65972.0	1.20	355.1050	-1.25	12.0	$C_{19}H_{17}O_6N_1$
367.1063	52355.0	0.95	367.1050	1.30	13.0	$C_{20}H_{17}O_6N_1$
368.1131	261514.0	4.76	368.1129	0.19	12.5	$C_{20}H_{18}O_6N_1$
369.1200	199591.0	3.63	369.1207	-0.74	12.0	$C_{20}H_{19}O_6N_1$
370.1230	45752.0	0.83	370.1226	0.31	20.5	$C_{27}H_{16}O_1N_1$
379.1063	40499.0	0.74	379.1050	1.22	14.0	$C_{21}H_{17}O_6N_1$
380.1135	88687.0	1.61	380.1129	0.63	13.5	$C_{21}H_{18}O_6N_1$
382.1266	152078.0	2.77	382.1285	-1.87	12.5	$C_{21}H_{20}O_6N_1$
383.1333	69523.0	1.26	383.1305	2.79	21.0	$C_{28}H_{17}O_1N_1$
458.1953	50858.0	0.93	458.1962	-0.90	15.5	$C_{28}H_{28}O_5N_1$
459.2020	79032.0	1.44	459.2040	-1.98	15.0	$C_{28}H_{29}O_5N_1$
473.2203	403350.0	7.34	473.2197	0.65	15.0	$C_{29}H_{31}O_5N_1$
484.2105	72810.0	1.32	484.2118	-1.39	16.5	$C_{30}H_{30}O_5N_1$
498.2279	345037.0	6.28	498.2275	0.39	16.5	$C_{31}H_{32}O_5N_1$
500.2164	48804.0	0.89	500.2193	-2.96	16.0	$C_{31}H_{32}O_6$
501.2151	90272.0	1.64	501.2146	0.53	16.0	$C_{30}H_{31}O_6N_1$
515.2304	356218.0	6.48	515.2302	0.11	16.0	$C_{31}H_{33}O_6N_1$
526.2218	90301.0	1.64	526.2224	-0.65	17.5	$C_{32}H_{32}O_6N_1$
529.2459	35803.0	0.65	529.2459	0.02	16.0	$C_{32}H_{35}O_6N_1$

23n HREI-MS _DLX-02-59A3-c1 12/6/2018 4:52:48 PM

EIH-2018120514-ZWL-DLX_DLX-02-59A3-c1#4 RT: 7.23
T: + c EI Full ms [49.50-800.50]
m/z= 48-803

m/z	Intensity	Relative	Theo. Mass	Delta (mmu)	RDB equiv.	Composition
50.0135	201675.0	4.05	50.0151	-1.58	4.0	C_4H_2
51.9966	227941.0	4.58	51.9944	2.26	4.0	C_3O_1
63.0239	378199.0	7.60	63.0229	0.96	4.5	C_5H_3
65.0388	252269.0	5.07	65.0386	0.22	3.5	C_5H_5
75.0227	177170.0	3.56	75.0229	-0.25	5.5	C_6H_3
75.0438	236862.0	4.76	75.0441	-0.21	0.5	$C_3H_7O_2$
77.0390	351493.0	7.07	77.0386	0.40	4.5	C_6H_5
89.0391	226502.0	4.55	89.0386	0.57	5.5	C_7H_5
91.0548	1111091.0	22.34	91.0542	0.62	4.5	C_7H_7
93.0701	207808.0	4.18	93.0699	0.20	3.5	C_7H_9
103.0552	177991.0	3.58	103.0542	1.02	5.5	C_8H_7
105.0708	1927946.0	38.76	105.0699	0.93	4.5	C_8H_9
115.0543	590762.0	11.88	115.0542	0.09	6.5	C_9H_7
117.0693	1287498.0	25.88	117.0699	-0.57	5.5	C_9H_9
118.0772	871235.0	17.52	118.0777	-0.49	5.0	C_9H_{10}
119.0847	291213.0	5.85	119.0855	-0.87	4.5	C_9H_{11}
131.0853	312520.0	6.28	131.0855	-0.26	5.5	$C_{10}H_{11}$
133.1006	4974026.0	100.00	133.1012	-0.62	4.5	$C_{10}H_{13}$
139.0534	176318.0	3.54	139.0542	-0.86	8.5	$C_{11}H_7$
178.0640	184330.0	3.71	178.0651	-1.11	10.5	$C_{13}H_8N_1$
222.0554	177287.0	3.56	222.0550	0.44	11.5	$C_{14}H_8O_2N_1$
236.0699	195776.0	3.94	236.0706	-0.66	11.5	$C_{15}H_{10}O_2N_1$
252.0644	176935.0	3.56	252.0655	-1.13	11.5	$C_{15}H_{10}O_3N_1$
264.0678	294794.0	5.93	264.0655	2.25	12.5	$C_{16}H_{10}O_3N_1$
265.0752	195658.0	3.93	265.0733	1.87	12.0	$C_{16}H_{11}O_3N_1$
278.0801	194749.0	3.92	278.0812	-1.05	12.5	$C_{17}H_{12}O_3N_1$
282.0741	248865.0	5.00	282.0761	-1.97	11.5	$C_{16}H_{12}O_4N_1$
292.0607	268088.0	5.39	292.0604	0.24	13.5	$C_{17}H_{10}O_4N_1$
293.0669	237596.0	4.78	293.0683	-1.33	13.0	$C_{17}H_{11}O_4N_1$
294.0752	285461.0	5.74	294.0761	-0.85	12.5	$C_{17}H_{12}O_4N_1$
296.0929	258051.0	5.19	296.0917	1.21	11.5	$C_{17}H_{14}O_4N_1$
306.0755	250685.0	5.04	306.0761	-0.62	13.5	$C_{18}H_{12}O_4N_1$
308.0910	502045.0	10.09	308.0917	-0.78	12.5	$C_{18}H_{14}O_4N_1$
322.0689	194132.0	3.90	322.0710	-2.09	13.5	$C_{18}H_{12}O_5N_1$
322.1045	312813.0	6.29	322.1074	-2.85	12.5	$C_{19}H_{16}O_4N_1$
324.0851	446021.0	8.97	324.0866	-1.51	12.5	$C_{18}H_{14}O_5N_1$
336.0865	381897.0	7.68	336.0866	-0.10	13.5	$C_{19}H_{14}O_5N_1$
337.0933	404641.0	8.14	337.0945	-1.15	13.0	$C_{19}H_{15}O_5N_1$
338.1010	238564.0	4.80	338.1023	-1.25	12.5	$C_{19}H_{16}O_5N_1$
350.1026	931015.0	18.72	350.1023	0.26	13.5	$C_{20}H_{16}O_5N_1$
351.1099	1212222.0	24.37	351.1101	-0.27	13.0	$C_{20}H_{17}O_5N_1$
352.1188	1652755.0	33.23	352.1179	0.83	12.5	$C_{20}H_{18}O_5N_1$
353.1215	410129.0	8.25	353.1199	1.62	21.0	$C_{27}H_{15}N_1$
367.1044	234309.0	4.71	367.1050	-0.64	13.0	$C_{20}H_{17}O_6N_1$
382.1284	1193293.0	23.99	382.1285	-0.08	12.5	$C_{21}H_{20}O_6N_1$
383.1336	447870.0	9.00	383.1363	-2.71	12.0	$C_{21}H_{21}O_6N_1$
484.2089	3257587.0	65.49	484.2118	-2.96	16.5	$C_{30}H_{30}O_5N_1$
515.2295	351023.0	7.06	515.2302	-0.73	16.0	$C_{31}H_{33}O_6N_1$

230 HREI-MS _DLX-02-62A1 -c1 12/11/2018 3:29:19 PM

EIH-2018120715-ZWL-DLX_DLX-02-62A1 -c1#3 RT: 7.02
T: + c EI Full ms [49.50-800.50]
m/z= 48-803

m/z	Intensity	Relative	Theo. Mass	Delta (mmu)	RDB equiv.	Composition
51.0238	346093.0	8.37	51.0229	0.89	3.5	C_4H_3
51.9931	1392679.0	33.67	51.9944	-1.26	4.0	C_3O_1
58.0773	78827.0	1.91	58.0777	-0.40	0.0	C_4H_{10}
60.0234	174411.0	4.22	60.0206	2.84	1.0	$C_2H_4O_2$
62.0164	117506.0	2.84	62.0151	1.34	5.0	C_5H_2
63.0240	261044.0	6.31	63.0229	1.12	4.5	C_5H_3
65.0394	357186.0	8.63	65.0386	0.79	3.5	C_5H_5
67.0544	210538.0	5.09	67.0542	0.17	2.5	C_5H_7
69.0701	422367.0	10.21	69.0699	0.19	1.5	C_5H_9
70.0762	201440.0	4.87	70.0777	-1.51	1.0	C_5H_{10}
71.0841	439154.0	10.62	71.0855	-1.45	0.5	C_5H_{11}
73.0270	154220.0	3.73	73.0284	-1.36	1.5	$C_3H_5O_2$
74.0135	113192.0	2.74	74.0151	-1.55	6.0	C_6H_2
75.0221	128658.0	3.11	75.0229	-0.78	5.5	C_6H_3
75.0430	160764.0	3.89	75.0441	-1.08	0.5	$C_3H_7O_2$
76.0300	122466.0	2.96	76.0308	-0.80	5.0	C_6H_4
77.0378	685055.0	16.56	77.0386	-0.74	4.5	C_6H_5
78.0455	299079.0	7.23	78.0464	-0.90	4.0	C_6H_6
79.0538	238828.0	5.77	79.0542	-0.45	3.5	C_6H_7
81.0699	173178.0	4.19	81.0699	0.05	2.5	C_6H_9
82.0775	93383.0	2.26	82.0777	-0.22	2.0	C_6H_{10}
83.0856	342483.0	8.28	83.0855	0.11	1.5	C_6H_{11}
84.0930	84138.0	2.03	84.0934	-0.33	1.0	C_6H_{12}
85.1011	278917.0	6.74	85.1012	-0.07	0.5	C_6H_{13}
89.0384	122965.0	2.97	89.0386	-0.21	5.5	C_7H_5
91.0544	715841.0	17.30	91.0542	0.17	4.5	C_7H_7
93.0701	78827.0	1.91	93.0699	0.20	3.5	C_7H_9
95.0858	127925.0	3.09	95.0855	0.30	2.5	C_7H_{11}
97.1016	276129.0	6.68	97.1012	0.44	1.5	C_7H_{13}
99.1175	101424.0	2.45	99.1168	0.65	0.5	C_7H_{15}
108.0464	147852.0	3.57	108.0444	2.03	4.5	$C_6H_6O_1N_1$
111.1055	147030.0	3.55	111.1043	1.28	2.0	$C_7H_{13}N_1$
121.0669	303451.0	7.34	121.0648	2.11	4.5	$C_8H_9O_1$
123.0829	86662.0	2.09	123.0804	2.45	3.5	$C_8H_{11}O_1$
128.0636	87748.0	2.12	128.0621	1.59	7.0	$C_{10}H_8$
129.0556	92502.0	2.24	129.0546	0.96	2.5	$C_6H_9O_3$
135.0437	104623.0	2.53	135.0441	-0.35	5.5	$C_8H_7O_2$
139.0536	85987.0	2.08	139.0542	-0.67	8.5	$C_{11}H_7$
149.0225	166428.0	4.02	149.0233	-0.80	6.5	$C_8H_5O_3$
151.0752	1759316.0	42.53	151.0754	-0.12	4.5	$C_9H_{11}O_2$
152.0807	294207.0	7.11	152.0832	-2.44	4.0	$C_9H_{12}O_2$
163.1113	88012.0	2.13	163.1117	-0.48	4.5	$C_{11}H_{15}O_1$
166.0645	86633.0	2.09	166.0651	-0.67	9.5	$C_{12}H_8N_1$
175.1111	83874.0	2.03	175.1117	-0.61	5.5	$C_{12}H_{15}O_1$
178.0648	80675.0	1.95	178.0651	-0.35	10.5	$C_{13}H_8N_1$
185.0357	206341.0	4.99	185.0386	-2.86	13.5	$C_{15}H_5$
186.0445	276716.0	6.69	186.0464	-1.94	13.0	$C_{15}H_6$
188.0413	86398.0	2.09	188.0401	1.25	-0.5	$C_3H_{10}O_8N_1$
191.1432	1015301.0	24.54	191.1430	0.15	4.5	$C_{13}H_{19}O_1$
206.1664	123024.0	2.97	206.1665	-0.08	4.0	$C_{14}H_{22}O_1$
236.0696	83845.0	2.03	236.0706	-0.98	11.5	$C_{15}H_{10}O_2N_1$
264.0676	132356.0	3.20	264.0655	2.09	12.5	$C_{16}H_{10}O_3N_1$
265.0730	88658.0	2.14	265.0733	-0.30	12.0	$C_{16}H_{11}O_3N_1$
278.0801	89656.0	2.17	278.0812	-1.07	12.5	$C_{17}H_{12}O_3N_1$
282.0774	78973.0	1.91	282.0761	1.32	11.5	$C_{16}H_{12}O_4N_1$

EIH-2018120715-ZWL-DLX_DLX-02-62A1 -c1#3 RT: 7.02
T: + c EI Full ms [49.50-800.50]
m/z= 48-803

m/z	Intensity	Relative	Theo. Mass	Delta (mmu)	RDB equiv.	Composition
292.0598	147529.0	3.57	292.0604	-0.61	13.5	$C_{17}H_{10}O_4N_1$
293.0672	106384.0	2.57	293.0683	-1.09	13.0	$C_{17}H_{11}O_4N_1$
294.0737	135731.0	3.28	294.0734	0.26	8.0	$C_{14}H_{14}O_7$
296.0908	106883.0	2.58	296.0917	-0.96	11.5	$C_{17}H_{14}O_4N_1$
306.0751	125900.0	3.04	306.0761	-1.01	13.5	$C_{18}H_{12}O_4N_1$
307.0825	97873.0	2.37	307.0812	1.23	8.5	$C_{15}H_{15}O_7$
308.0910	201880.0	4.88	308.0917	-0.69	12.5	$C_{18}H_{14}O_4N_1$
322.0723	88364.0	2.14	322.0710	1.35	13.5	$C_{18}H_{12}O_5N_1$
322.1074	166986.0	4.04	322.1074	-0.01	12.5	$C_{19}H_{16}O_4N_1$
324.0876	193252.0	4.67	324.0866	0.99	12.5	$C_{18}H_{14}O_5N_1$
336.0867	171770.0	4.15	336.0866	0.01	13.5	$C_{19}H_{14}O_5N_1$
337.0928	135643.0	3.28	337.0918	0.97	8.5	$C_{16}H_{17}O_8$
338.1032	101571.0	2.46	338.1023	0.88	12.5	$C_{19}H_{16}O_5N_1$
350.1030	544599.0	13.17	350.1023	0.71	13.5	$C_{20}H_{16}O_5N_1$
351.1089	658466.0	15.92	351.1101	-1.22	13.0	$C_{20}H_{17}O_5N_1$
352.1173	648283.0	15.67	352.1179	-0.66	12.5	$C_{20}H_{18}O_5N_1$
353.1206	131417.0	3.18	353.1199	0.75	21.0	$C_{27}H_{15}N_1$
367.1047	124344.0	3.01	367.1050	-0.32	13.0	$C_{20}H_{17}O_6N_1$
382.1275	392785.0	9.50	382.1285	-1.06	12.5	$C_{21}H_{20}O_6N_1$
383.1342	123581.0	2.99	383.1363	-2.11	12.0	$C_{21}H_{21}O_6N_1$
502.1865	1163681.0	28.13	502.1860	0.45	16.5	$C_{29}H_{28}O_7N_1$
530.1810	80881.0	1.96	530.1809	0.10	17.5	$C_{30}H_{28}O_8N_1$
533.2031	86662.0	2.09	533.2044	-1.33	16.0	$C_{30}H_{31}O_8N_1$
