# Supplementary material for: Discovery of dihydrooxazolo[2,3-a]isoquinoliniums as highly specific inhibitors of hCE2
Source: RSC Adv. 2019 Nov 4;9(61):35904–12. doi: 10.1039/c9ra07457k (PMC9074662; doi:10.1039/c9ra07457k)

Electronic Supplementary Material (ESI) for RSC Advances.
This journal is © The Royal Society of Chemistry 2019

Electronic Supplementary Material (ESI) for RSC Advances.

Supporting Information

Discovery of dihydrooxazolo[2,3-a]isoquinoliniums as highly specific inhibitors of hCE2

Lixia Ding† [a,c] , Lu Wang† [b] , Kun Zou† [a] , Bo Li*[a,d] , Yunqing Song [b], Qihua Zhang[a], Yitian Zhao [b], Zhijian Xu[a,d], Guangbo Ge*[b], Bo Zhao*[c], Weiliang Zhu*[a,d,e]

[a.]Key Laboratory of Receptor Research, Drug Discovery and Design Center, Shanghai Institute of Materia Medica, Chinese Academy of Sciences, 555 Zuchongzhi Road, Shanghai 201203, China

[b.]Shanghai University of Traditional Chinese Medicine, 1200 Cailun Road, Shanghai 201203, China

[c.]College of Chemistry and Environmental Science, Nanjing Normal University, 1 Wenyuan Road, Nanjing 210097, China.

[d.]University of Chinese Academy of Sciences, No.19A Yuquan Road, Beijing 100049, China

Open Studio for Druggability Research of Marine Natural Products, Pilot National Laboratory for [e.]Marine Science and Technology (Qingdao), 1 Wenhai Road, Aoshanwei, Jimo, Qingdao, 266237, China

† These authors made equal contributions to this work.

* Corresponding Authors: boli@simm.ac.cn (Bo Li), geguangbo@dicp.ac.cn (Guangbo Ge), zhaobo@njnu.edu.cn (Bo Zhao), wlzhu@simm.ac.cn (Weiliang Zhu).

Electronic Supplementary Information (ESI) available: See DOI:10.1039/x0xx00000x

1. Biological assay

Table S1. The chemical structures of probe substrates towards hCE1 and hCE2.

Probe	Substrate structure and hydrolytic site(s)	Target enzyme	Enzyme source	Detection conditions
**DME**	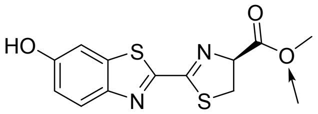	hCE1	HLM	Bioluminescence<br>λ_{em} = 580 nm (M)
**FD**	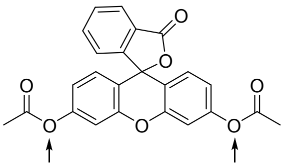	hCE2	HLM	Fluorescence<br>$\lambda_{ex/em}$ = 480/525 nm (M)

*M means the hydrolytic metabolite

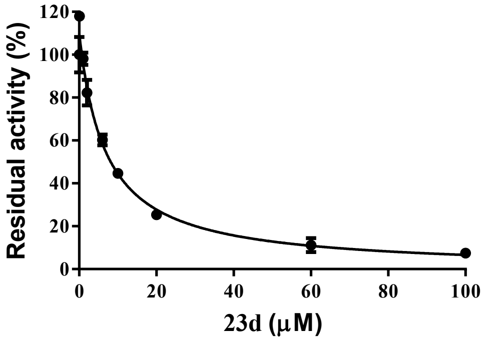

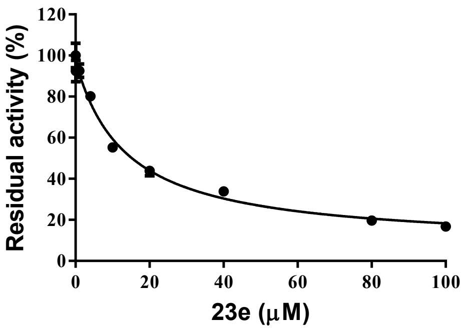

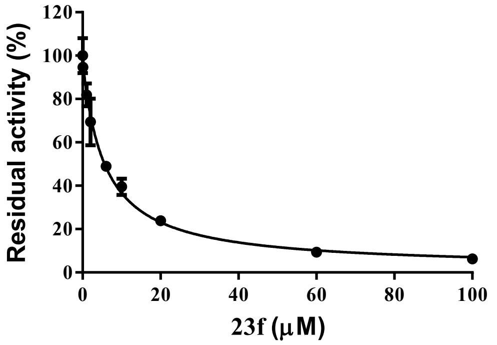

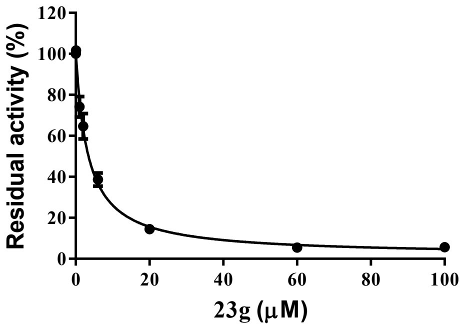

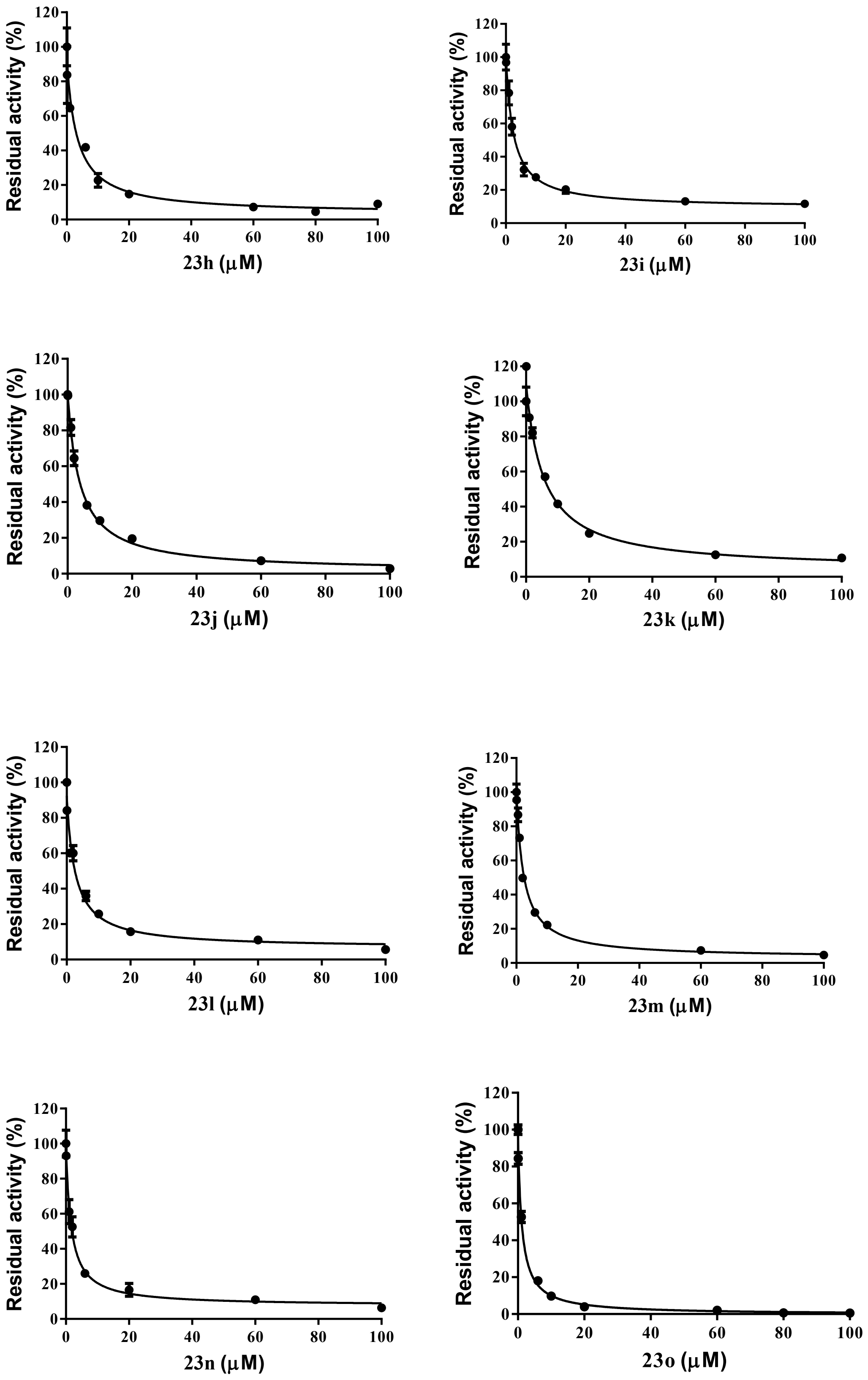

Fig. S1 The dose-inhibition dependent curves of compounds **23d-23o**.

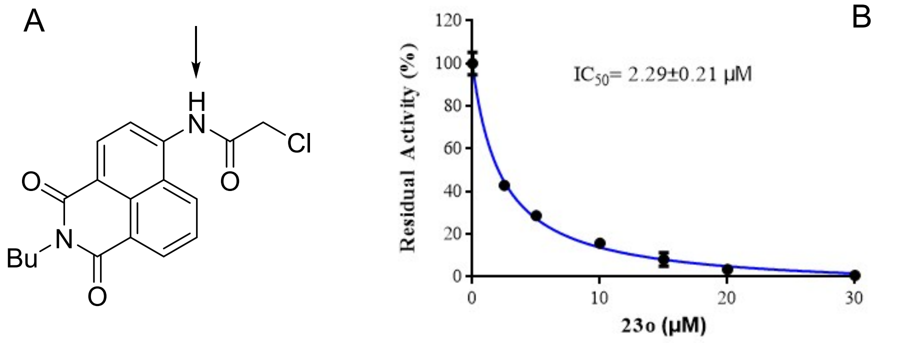

Fig S2 The structure and hydrolytic site of NCEN (A) and the dose inhibition curve of **23o** against intracellular hCE2 in living HepG2 cells (B).

2. Molecular Docking.

The crystal structure of hCE2 at 3.1 Å resolutions was selected for subsequent molecular docking. To generate the proper protonation states, we used the Epik 3.4 (Schrödinger, LLC, New York, NY, USA) of Ligprep 3.6 software (Schrödinger, LLC, New York, NY, USA) to pretreat compound **23o**. Molecular docking was performed by Glide 6.9 (Schrödinger, LLC, New York, NY, USA) in SP (standard precision) mode, and the other parameters were used with default values.

3. Experimental protocols

3.1 Chemistry general procedures

All starting materials were obtained from commercial suppliers and used without further purification. The ^{1}H NMR and ^{13}C NMR spectra were taken on Bruker Avance-500 or 400, Varian-MERCURY Plus-400 or 300 NMR spectrometer operating at 400 MHz or 300 MHz for ^{1}H NMR, 125 MHz or 100 MHz for ^{13}C NMR, using TMS as internal standard and $CDCl_3$ or Methanol-d_4 or DMSO-d_6 as solvent. ^{13}C NMR spectra were recorded with complete proton decoupling. The ESI-MS or EI-MS was recorded on Finnigan LCQ/DECA or Thermo-DFS, respectively. The HRMS were obtained from Micromass Ultra Q-TOF (ESI) or Thermo- DFS (EI) spectrometer. Flash column chromatography was carried out using silica gel (200-400 mesh). Thin layer chromatography (TLC) was used silica gel F254 fluorescent treated silica which was visualized under UV light (254 nm).

3.2 Synthesis and characterization of some compounds

1-(3,4-dimethoxyphenyl)-N, N-dimethylmethanamine (2).

To a solution of **1** (16.6 g, 100 mmol) in DMF (30.8 mL) was added 90% formic acid (2 mL). The reaction mixture was heated at 150℃ for 2 hours, and then the water of the reaction mixture was distilled out. Additional DMF (15 mL) and formic acid (1 mL) were added repeatedly until the starting materials were consumed completely. Then DMF of the reaction mixture was removed under vacuum and the concentrated hydrochloric acid was added to that to be acidity. Ethyl acetate (100 ml) and water (100 ml) were added for stirring about 20 min. After separating the organic phase, the water phase was adjusted to PH 6 with 40% NaOH and then it was extracted with ethyl acetate (150ml*3). The combined ethyl acetate phases were dried over Na_2SO_4 and evaporated under vacuum to give the yellowish oily product **2** (13 g, 75%). ^{1}H NMR (400 MHz, $CDCl_3$) δ 6.90 (s, 1H), 6.82 (d, *J* = 1.1 Hz, 2H), 3.91 (s, 3H), 3.89 (s, 3H), 3.37 (s, 2H), 2.24 (s, 6H). ^{13}C NMR (126 MHz, DMSO-d_6) δ 148.6, 148.2, 121.5, 112.8, 111.4, 62.1, 55.4 (d, *J* = 6.7 Hz), 43.8. HRMS (ESI) calcd for $C_{11}H_{18}NO_2$ 196.1332 $[M+H]^+$, found 196.1334.

Methyl 6-(chloromethyl)-2,3-dimethoxybenzoate（16a）

To a solution of **2** (2 g, 10 mmol) in anhydrous THF (20 mL) under nitrogen was added n-butyllithium (2.5M in THF, 5 mL, 12.5 mmol) under ice bath, stirring for 1 hour at room temperature. Methyl chloroformate (1.63 mL, 21 mmol) was instilled under -78℃, and then the reaction mixture was moved to room temperature. After stirring overnight, the reaction mixture was quenched with excess water and extracted with ethyl acetate. The combined organic layers were washed with water and brine, dried over Na_2SO_4, concentrated, and purified by silica gel (PE: EA = 10:1) to afford the colorless oily product **16a** (1.93 g, 80%). ^{1}H NMR (400 MHz, $CDCl_3$) δ 7.13 (d, *J* = 8.4 Hz, 1H), 6.94 (d, *J* = 8.4 Hz, 1H), 4.61 (s, 2H), 3.98 (s, 3H), 3.90 (s, 3H), 3.89 (s, 3H). ^{13}C NMR (150 MHz, $CDCl_3$) δ 166.7, 152.6, 146.3, 128.2, 126.9, 125.3, 112.9, 61.1, 55.5, 52.1, 43.2. HRMS (EI) calcd for $C_{11}H_{13}O_4Cl$ 244.0497 $[M]^+$, found 244.0497.

Methyl 6-(cyanomethyl)-2,3-dimethoxybenzoate(17a)

To a solution of **16** (488.1 mg, 2 mmol) in acetonitrile (10.0 mL) was added TMSCN (0.4 mL, 3 mmol) and TBAF (1M in THF, 3 mL, 3 mmol) under nitrogen, stirring overnight at 80℃.The reaction mixture was quenched with excess water at room temperature and extracted with ethyl acetate. The combined organic layers were washed with water and brine, dried over Na_2SO_4, concentrated, and purified by silica gel (PE: EA = 25:1) to afford the lilac oily product **17a** (385 mg, 82%). ^{1}H NMR (400 MHz, $CDCl_3$) δ 7.18 (d, *J* = 8.4 Hz, 1H), 6.99 (d, *J* = 8.4 Hz, 1H), 3.98 (s, 2H), 3.91 (s, 3H), 3.90 (s, 3H), 3.75 (s, 2H). ^{13}C NMR (125 MHz,

$CDCl_3$) δ 167.0, 152.8, 147.4, 127.9, 124.6, 119.9, 117.4, 114.1, 61.6, 56.0, 52.6, 21.2. HRMS (EI) calcd for $C_{12}H_{13}O_4N$ 235.0839 $[M]^+$, found 235.0845.

6-(cyanomethyl)-2,3-dimethoxybenzoic acid (15)

To a solution of **17** (2.35 g, 10 mmol) in 1, 4-dioxane (20.0 mL) was added NaOH（2.9M）to adjust the pH to about 10, stirring for 5h at 50℃. The reaction mixture was extracted with ethyl acetate and discarded the organic phase, modulating pH value of the water phase to about 3 with HCl (3M) before extracting with ethyl acetate. The combined organic layers were washed with water and brine, dried over Na_2SO_4, and concentrated to obtain the solid product **15** (1.95 g, 88%).^{1}H NMR (400 MHz, $CDCl_3$) δ 7.41 (d, J = 8.8 Hz, 1H), 7.15 (d, J = 8.8 Hz, 1H), 4.14 (s, 2H), 4.06 (s, 3H), 3.96 (s, 3H). ^{13}C NMR (125 MHz, $CDCl_3$) δ 166.0, 152.3, 148.6, 126.3, 124.0, 122.7, 117.9, 115.7, 62.4, 56.2, 22.8. HRMS (EI) calcd for $C_{14}H_{16}O_4$ 248.1043 $[M]^+$, found 248.1041.

1,3-dichloro-7,8-dimethoxyisoquinoline (12)

To a solution of **15** (2.21 g, 10 mmol) in 1, 4-dioxane (20.0 mL) was added phosphorus pentachloride (2.85 g, 13.7 mmol), stirring overnight at 90℃. The reaction mixture was quenched with excess iced water and extracted with ethyl acetate. The combined organic layers were washed with water and brine, dried over Na_2SO_4, concentrated, and purified by silica gel (PE: EA = 30:1) to afford the light yellow fluffy product **12** (1.4 g, 55%).^{1}H NMR (400 MHz, DMSO-d_6) δ 8.05 (s, 1H), 7.93 (d, J = 8.8 Hz, 1H), 7.87 (d, J = 8.8 Hz, 1H), 4.00 (s, 3H), 3.87 (s, 3H). ^{13}C NMR (125 MHz, $CDCl_3$) δ 151.9, 146.8, 144.2, 140.5, 135.6, 122.8, 120.9, 119.3, 61.7, 57.0. HRMS (ESI) calcd for $C_{11}H_{10}Cl_2NO_2$ 258.0083 $[M+H]^+$, found 258.0087.

Propyl 6-(chloromethyl)-2,3-dimethoxybenzoate (16b)

To a solution of **2** (2.0 g, 10 mmol) in anhydrous THF (20 mL) under nitrogen was dropwise added n-butyllithium (2.5M in THF, 5 mL, 12.5 mmol) under ice bath, stirring for 1 hour at room temperature. Propyl chlorocarbonate (2.25 mL, 20 mmol) was instilled under -78℃, and then the reaction mixture was moved to room temperature. After stirring overnight, the reaction mixture was quenched with excess water and extracted with ethyl acetate. The combined organic layers were washed with water and brine, dried over Na_2SO_4, concentrated, and purified by silica gel (PE: EA = 10:1) to afford the colorless oily product **16b** (2.1 g, 77%). ^{1}H NMR (400 MHz, $CDCl_3$) δ 7.12 (d, *J* = 8.4 Hz, 1H), 6.92 (d, *J* = 8.4 Hz, 1H), 4.62 (s, 2H), 4.34 (t, *J* = 6.8, 2H), 3.89 (s, 6H), 1.88 – 1.75 (m, 2H), 1.03 (t, *J* = 7.2, 3H). ^{13}C NMR (125 MHz, $CDCl_3$) δ 166.3, 152.6, 146.2, 128.6, 126.9,

125.3, 112.8, 66.8, 61.0, 55.4, 43.1, 21.5, 10.0. HRMS (EI) calcd for $C_{13}H_{17}O_4Cl$ 272.0810 $[M]^+$, found 272.0818.

n-Propyl 6-(cyanomethyl)-2,3-dimethoxybenzoate (17b)

To a solution of **16** (2.1 g, 7.7 mmol) in acetonitrile (10.0 mL) was added TMSCN (1.44 mL, 11.5 mmol) and TBAF (11.55 mL, 11.5 mmol) under nitrogen, stirring overnight at 80℃. The reaction mixture was quenched with excess water and extracted with ethyl acetate. The combined organic layers were washed with water and brine, dried over Na_2SO_4, concentrated, and purified by silica gel (PE: EA = 25:1) to afford the brown oily product **17b** (1.3 g, 64%). ^1H NMR (400 MHz, $CDCl_3$) δ 7.18 (d, J = 8.8 Hz, 1H), 6.99 (d, J = 8.4 Hz, 1H), 4.35 (t, J = 7.6 Hz, 2H), 3.91 (s, 3H), 3.90 (s, 3H), 3.75 (s, 2H), 1.82 (h, J = 7.2 Hz, 2H), 1.05 (t, J = 7.4 Hz, 3H). ^{13}C NMR (150 MHz, $CDCl_3$) δ 166.2, 152.3, 146.7, 127.8, 124.1, 119.4, 117.0, 113.4, 67.0, 61.1, 55.5, 21.5, 20.7, 10.0. HRMS (EI) calcd for $C_{14}H_{17}O_4 N_1$ 263.1152 $[M]^+$, found 263.1167.

6-(carboxymethyl)-2,3-dimethoxybenzoic acid (18)

To a solution of **17** (20 g, 76 mmol) in 1, 4-dioxane (50.0 mL) was added NaOH（10M）, stirring overnight at 100℃. The reaction mixture was extracted with ethyl acetate and then discarded the organic phase, modulating pH value of the water phase to about 3 with HCl (3M) before extracting with ethyl acetate. The combined organic layers were washed with water and brine, dried over Na_2SO_4, concentrated under vacuum to obtain the sandy brown solid product **18** (13.94 g, 76%). ^1H NMR (400 MHz, $CDCl_3$) δ 9.46 (s, 2H), δ 7.00 (d, J = 8.8 Hz, 1H), 6.98 (d, J = 8.4 Hz, 1H), 3.94 (s, 3H), 3.90 (s, 3H), 3.83 (s, 2H). ^{13}C NMR (125 MHz, $CDCl_3$) δ 166.0, 152.3, 148.6, 126.3, 124.0, 122.7, 117.9, 115.7, 62.4, 56.2, 22.8. HRMS (ESI) calcd for $C_{11}H_{13}O_6$ 241.0707 $[M+H]^+$, found 241.0708.

7,8-dimethoxyisochroman-1,3-dione (19)

To a solution of **18** (1.0 g, 4 mmol) in acetylchloride (5.0 mL) stirred for 5h at 50℃. Removing the superfluous acetyl chloride, washing the mixture with petroleum ether, and filtering, the sandy yellowish-brown product **19** was obtained (0.72 g, 78%).^1H NMR (400 MHz, $CDCl_3$) δ 7.23 (d, J = 7.6 Hz, 1H), 7.03 (d, J = 7.6 Hz, 1H), 4.02 (s, 2H), 4.00 (s, 3H), 3.94 (s, 3H). ^{13}C NMR (150 MHz, DMSO-d_6) δ 166.0, 157.7, 152.2, 150.4, 127.8, 122.9, 119.5, 115.7, 60.7, 56.3, 34.0. HRMS (EI) calcd for $C_{11}H_{10}O_5$ 222.0523 $[M]^+$, found 222.0524.

7,8-dimethoxyisoquinoline-1,3(2H,4H)-dione (20)

Compound **19** (500 mg, 2.2 mmol) and ammonium carbonate (650 mg, 6.7 mmol) was placed in a mortar and grind evenly, and then slowly heated to fusion at about 280℃. After

stirring for 2 h, the reaction mixture was cooled to room temperature. The residue was dissolved with dichloromethane and purified by silica gel (DCM: MeOH = 50:1) to afford the white solid produce **20** (114 mg, 22%). ^{1}H NMR (400 MHz, $CDCl_3$) δ 8.03 (s, 1H), 7.20 (d, *J* = 8.8 Hz, 1H), 7.03 (d, *J* = 8.8 Hz, 1H), 3.96 (s, 3H), 3.93 (s, 3H). ^{13}C NMR (125 MHz, DMSO-d_6) δ 171.2, 163.7, 152.7, 150.3, 129.3, 123.8, 119.3, 118.4, 61.1, 56.6, 36.2. HRMS (ESI) calcd for $C_{11}H_{11}NNaO_4$ 244.058 $[M+Na]^+$, found 244.0583.

3,7,8-trimethoxyisoquinolin-1(4H)-one (21)

To a solution of **17** (2.54 g, 9.6 mmol) in anhydrous methanol (50 mL) was added MeONa (1.39 g, 24 mmol) at room temperature, heating at 80℃ for 1 hour. The reaction mixture was quenched with excess water and extracted with ethyl acetate. The combined organic layers were washed with water and brine, dried over Na_2SO_4, concentrated, and purified by silica gel (PE: EA = 1:1) to afford the yellow flocculent solid product **21** (1.16 g, 51%). ^{1}H NMR (400 MHz, $CDCl_3$) δ 8.66 (s, 1H), 7.29 (d, *J* = 8.8 Hz, 1H), 7.15 (d, *J* = 8.8 Hz, 1H), 5.62 (s, 1H), 3.99 (s, 3H), 3.93 (s, 3H), 3.88 (s, 3H). ^{13}C NMR (125 MHz, DMSO-d_6) δ 160.6, 152.1, 149.3 (d, *J* = 4.0 Hz), 135.3, 121.5, 120.2, 117.1, 80.0, 61.3, 57.0, 55.8. HRMS (ESI) calcd for $C_{12}H_{14}NO_4$ 236.0917 $[M+H]^+$, found 236.0922.

3-chloro-1-(2,2-dimethoxyethoxy)-7,8-dimethoxyisoquinoline(10)

To a solution of glycolaldehyde dimethyl acetal (836μL, 6.54 mmol) in anhydrous THF (15 mL) was added NaH (262 mg, 6.54 mmol) under ice-water condition bath, stirring for 1 hour at room temperature. **12** (1.4 g, 5.45 mmol) was added and the reaction mixture was stirred overnight before quenching with saturated ammonium chloride. Water was added to the reaction mixture to separate out the white solid and then filter to gain the white solid (1.75 g, 98%). ^{1}H NMR (400 MHz, $CDCl_3$) δ 7.46 (d, *J* = 8.8 Hz, 1H), 7.43 (d, *J* = 8.8 Hz, 1H), 7.20 (s, 1H), 4.93 (t, *J* = 5.2 Hz, 1H), 4.58 (d, *J* = 5.6 Hz, 2H), 4.00 (s, 3H), 3.95 (s, 3H), 3.51 (s, 6H). ^{13}C NMR (150 MHz, DMSO-d_6) δ 158.4, 151.2, 144.2, 138.0, 135.2, 122.4, 120.4, 113.6, 113.1, 101.3, 65.4, 61.1, 56.7, 53.7. HRMS (EI) calcd for $C_{15}H_{18}O_5NCl$ 327.0868 $[M]^+$, found 327.0866.

1-(2,2-dimethoxyethoxy)-7,8-dimethoxy-3-(4-methoxy-3-((4-methoxybenzyl)oxy)phenyl)isoquinoline(22d)

To a solution of **10** (98 mg, 0.3 mmol) in 1, 4-dioxane (15 mL) was added (4-methoxy-3-((4-methoxybenzyl)oxy)phenyl)boronic acid (104 mg, 0.36 mmol), Xphos (28 mg, 0.06 mmol), $Pd_2(dba)_3$ (27 mg, 0.03 mmol) and K_3PO_4 (127 mg, 0.6 mmol) under nitrogen atmosphere, and then the reaction was raised to 100℃ for 9 hours. The reaction mixture was quenched with excess water and extracted with ethyl acetate. The combined organic

layers were washed with water and brine, dried over Na_2SO_4, concentrated, and purified by silica gel (PE: EA = 20:1) to afford the solid product **22d** (120 mg, 75%). ^{1}H NMR (400 MHz, $CDCl_3$) δ 7.77 (d, *J* = 2.0 Hz, 1H), 7.69 (dd, *J* = 8.4, 2.0 Hz, 1H), 7.53 (d, *J* = 8.8 Hz, 1H), 7.49 (s, 1H), 7.47 (d, *J* = 8.4 Hz, 2H), 7.43 (d, *J* = 8.8 Hz, 1H), 6.99 (d, *J* = 8.4 Hz, 1H), 6.95 (d, *J* = 8.4 Hz, 2H), 5.23 (s, 2H), 5.02 (t, *J* = 5.6 Hz, 1H), 4.69 (d, *J* = 5.2 Hz, 2H), 4.01 (s, 3H), 3.99 (s, 3H), 3.96 (s, 3H), 3.83 (s, 3H), 3.55 (s, 6H). ^{13}C NMR (125 MHz, $CDCl_3$) δ 159.4, 158.4, 150.8, 150.1, 148.3, 145.4, 145.1, 135.9, 131.9, 129.4, 129.2, 123.1, 119.2, 119.2, 114.0, 112.5, 111.8, 109.5, 102.3, 71.0, 65.1, 61.8, 57.2, 56.1, 55.3, 54.0. HRMS (EI) calcd for $C_{30}H_{33}O_8N$ 535.2201 $[M]^+$, found 535.2182.

1-(2,2-dimethoxyethoxy)-7,8-dimethoxy-3-(4-methoxy-3-((4-(methylsulfonyl)benzyl)ox-y)phenyl)isoquinoline(22e)

Compound **22e** was obtained from **10** (98 mg, 0.3 mmol) and (4-methoxy-3-((4-(methylsulfonyl) benzyl)oxy)phenyl)boronic acid (121 mg, 0.36 mmol) by following a similar procedure as that for **22d**, it was purified by silica gel (PE: EA = 15:1) as a solid (125 mg, 71%). ^{1}H NMR (400 MHz, $CDCl_3$) δ 8.01 (d, *J* = 8.0 Hz, 2H), 7.77 (s, 1H), 7.75 – 7.73 (m, 2H), 7.71 (dd, *J* = 8.4, 4.0 Hz, 1H), 7.54 (d, *J* = 9.2 Hz, 1H), 7.50 (s, 1H), 7.44 (d, *J* = 9.2 Hz, 1H), 7.02 (d, *J* = 8.4 Hz, 1H), 5.40 (s, 2H), 4.99 (t, *J* = 5.4 Hz, 1H), 4.61 (d, *J* = 5.2 Hz, 2H), 4.00 (s, 3H), 3.99 (s, 3H), 3.98 (s, 3H), 3.57 (s, 6H), 3.07 (s, 3H). ^{13}C NMR (125 MHz, $CDCl_3$) δ 158.4, 150.9, 150.0, 147.6, 145.4, 144.7, 143.9, 139.9, 135.7, 132.1, 127.9, 127.8, 123.1, 119.8, 119.2, 114.5, 112.5, 111.9, 109.5, 102.5, 70.2, 65.3, 61.8, 58.5, 57.1, 56.1, 54.2, 44.5, 18.5. HRMS (EI) calcd for $C_{30}H_{33}O_9NS$ 583.1871 $[M]^+$, found 583.1880.

Methyl4-((5-(1-(2,2-dimethoxyethoxy)-7,8-dimethoxyisoquinolin-3-yl)-2-methoxyphen-oxy)methyl)benzoate(22f)

Compound **22f** was obtained from **10** (130 mg, 0.4 mmol) and (4-methoxy-3-((4-(methoxycarbonyl)benzyl)oxy)phenyl)boronic acid (151 mg, 0.48 mmol) by following a similar procedure as that for **22d**, it was purified by silica gel (PE: EA = 20:1) as a solid (104 mg, 46%). ^{1}H NMR (400 MHz, $CDCl_3$) δ 8.09 (d, *J* = 8.0 Hz, 2H), 7.73 (d, *J* = 2.0 Hz, 1H), 7.71 (dd, *J* = 8.4, 2.0 Hz, 1H), 7.62 (d, *J* = 8.0 Hz, 2H), 7.52 (d, *J* = 8.8 Hz, 1H), 7.47 (s, 1H), 7.43 (d, *J* = 8.8 Hz, 1H), 7.01 (d, *J* = 8.4 Hz, 1H), 5.36 (s, 2H), 5.00 (t, *J* = 5.2 Hz, 1H), 4.64 (d, *J* = 5.2 Hz, 2H), 4.00 (s, 3H), 3.99 (s, 3H), 3.98 (s, 3H), 3.93 (s, 3H), 3.55 (s, 6H). ^{13}C NMR (125 MHz, $CDCl_3$) δ 166.9, 158.4, 150.9, 150.0, 147.9, 145.4, 144.8, 142.6, 135.8, 132.0, 130.0, 127.0, 123.1 119.6, 119.2, 112.5, 111.9, 109.4, 102.2, 70.6, 65.0, 61.8, 57.1, 56.1, 53.9, 52.1, 29.7. HRMS (EI) calcd for $C_{31}H_{33}O_9N$ 563.2150 $[M]^+$, found 563.2149.

4-((5-(1-(2,2-dimethoxyethoxy)-7,8-dimethoxyisoquinolin-3-yl)-2-methoxyphenoxy)me-thyl)benzonitrile(22g)

Compound **22g** was obtained from **10** (130 mg, 0.4 mmol) and (3-((4-cyanobenzyl)oxy)-4-methoxyphenyl)boronic acid (135 mg, 0.48 mmol) by following a similar procedure as that for **22d**, it was purified by silica gel (PE: EA = 20:1) as a solid (120 mg, 56%). ^{1}H NMR (400 MHz, $CDCl_3$) δ 7.75 – 7.68 (m, 4H), 7.66 (d, J = 8.0 Hz, 2H), 7.53 (d, J = 8.8 Hz, 1H), 7.49 (s, 1H), 7.43 (d, J = 8.8 Hz, 1H), 7.02 (d, J = 8.0 Hz, 1H), 5.36 (s, 2H), 4.99 (t, J = 5.2 Hz, 1H), 4.59 (d, J = 5.6 Hz, 2H), 4.00 (s, 3H), 3.99 (s, 3H), 3.98 (s, 3H), 3.57 (s, 6H). ^{13}C NMR (150 MHz, $CDCl_3$) δ 157.9, 150.4, 149.5, 147.1, 144.8, 144.1, 142.4, 135.2, 132.1, 131.5, 127.1, 122.6, 119.2, 118.6, 118.4, 114.0, 112.0, 111.3, 111.1, 109.1, 101.9, 69.7, 64.7, 61.4, 56.6, 55.6, 53.7, 29.3. HRMS (EI) calcd for $C_{30}H_{30}O_7N_2$ 530.2048 $[M]^+$, found 530.2054.

1-(2,2-dimethoxyethoxy)-7,8-dimethoxy-3-(4-methoxy-3-((4-nitrobenzyl)oxy)phenyl)is-oquinoline(22h)

Compound **22h** was obtained from **10** (98 mg, 0.3 mmol) and (4-methoxy-3-((4-nitrobenzyl)oxy)phenyl)boronic acid (138 mg, 0.36 mmol) by following a similar procedure as that for **22d**, it was purified by silica gel (PE: EA = 15:1) as a solid (144 mg, 87%). ^{1}H NMR (400 MHz, $CDCl_3$) δ 8.27 (d, J = 8.8 Hz, 2H), 7.75 – 7.63 (m, 4H), 7.50 (d, J = 8.8 Hz, 1H), 7.47 (s, 1H), 7.40 (d, J = 8.8 Hz, 1H), 7.00 (d, J = 8.4 Hz, 1H), 5.38 (s, 2H), 4.96 (t, J = 5.2 Hz, 1H), 4.56 (d, J = 5.2 Hz, 2H), 3.98 (s, 3H), 3.97 (s, 3H), 3.95 (s, 3H),, 3.53 (s, 6H). ^{13}C NMR (125 MHz, $CDCl_3$) δ 157.9, 150.4, 149.5, 147.1, 144.9, 144.5, 144.1, 135.2, 131.6, 127.1, 123.4, 122.6, 119.3, 118.7, 114.0, 112.1, 111.4, 109.0, 101.8, 69.5, 64.6, 61.3, 56.6, 55.6, 53.6. HRMS (EI) calcd for $C_{29}H_{30}O_9N_2$ 550.1946 $[M]^+$, found 550.1950.

1-(2,2-dimethoxyethoxy)-7,8-dimethoxy-3-(4-methoxy-3-((4-(trifluoromethyl)benzyl)o-xy)phenyl)isoquinoline(22i)

Compound **22i** was obtained from **10** (98 mg, 0.3 mmol) and (4-methoxy-3-((4-(trifluoromethyl)benzyl)oxy)phenyl)boronic acid (98 mg, 0.36 mmol) by following a similar procedure as that for **22d**, it was purified by silica gel (PE: EA = 15:1) as a solid (136 mg, 79%). ^{1}H NMR (400 MHz, $CDCl_3$) δ 7.81 – 7.63 (m, 6H), 7.53 (d, J = 8.8 Hz, 1H), 7.49 (s, 1H), 7.43 (d, J = 8.8 Hz, 1H), 7.02 (d, J = 8.0 Hz, 1H), 5.36 (s, 2H), 5.00 (t, J = 5.4 Hz, 1H), 4.64 (d, J = 5.6 Hz, 2H), 4.00 (s, 3H), 3.98 (s, 6H), 3.55 (s, 6H). ^{13}C NMR (125 MHz, $CDCl_3$) δ 157.9, 150.4, 149.5, 147.3, 144.9, 144.3, 141.0, 135.3, 131.5, 126.9, 125.1,

125.1, 122.6, 119.7, 119.1, 118.7, 113.9, 112.0, 111.4, 109.0, 101.8, 69.9, 64.6, 61.3, 56.6, 55.6, 53.5. HRMS (EI) calcd for $C_{30}H_{30}O_7NF_3$ 573.1969 $[M]^+$, found 573.1963.

7-((5-(1-(2,2-dimethoxyethoxy)-7,8-dimethoxyisoquinolin-3-yl)-2-methoxyphenoxy)me-thyl)quinolone(22j)

Compound **22j** was obtained from **10** (98 mg, 0.3 mmol) and (4-methoxy-3-(quinolin-7-ylmethoxy)phenyl)boronic acid (110 mg, 0.36 mmol) by following a similar procedure as that for **22d**, it was purified by silica gel (PE: EA = 15:1) as a solid (88 mg, 52%). ^{1}H NMR (400 MHz, $CDCl_3$) δ 9.01 (m, 1H), 8.22 (m, 1H), 8.07 (d, J = 7.1 Hz, 1H), 7.82 – 7.74 (m, 3H), 7.60 (t, J = 7.6 Hz, 1H), 7.52 – 7.46 (m, 2H), 7.41 (m, 2H), 7.04 (d, J = 8.8 Hz, 1H), 6.09 (s, 2H), 4.95 (m, 1H), 4.59 (d, J = 5.6 Hz, 2H), 4.02 (s, 3H), 3.99 (s, 3H), 3.96 (s, 3H), 3.50 (s, 6H). ^{13}C NMR (125 MHz, $CDCl_3$) δ 158.3, 150.8, 150.0, 149.5, 148.4, 145.7, 145.4, 145.2, 136.4, 135.8, 135.5, 132.1, 128.0, 127.6, 127.2, 126.6, 123.0, 121.1, 119.5, 119.2, 114.35, 112.0, 111.7, 109.5, 102.1, 67.0, 64.9, 61.8, 57.2, 56.2, 53.9. HRMS (EI) calcd for $C_{32}H_{32}O_7N_2$ 556.2204 $[M]^+$, found 556.2210.

1-(2,2-dimethoxyethoxy)-7,8-dimethoxy-3-(4-methoxy-3-((4-methylbenzyl)oxy)phenyl)-isoquinoline(22k)

Compound **22k** was obtained from **10** (98 mg, 0.3 mmol) and (4-methoxy-3-((4-methylbenzyl)oxy)phenyl)boronic acid (98 mg, 0.36 mmol) by following a similar procedure as that for **22d**, it was purified by silica gel (PE: EA = 30:1) as a solid (153 mg, 98%). ^{1}H NMR (400 MHz, $CDCl_3$) δ 7.76 (s, 1H), 7.69 (m, 1H), 7.53 (d, J = 8.8 Hz, 1H), 7.48 (s, 1H), 7.43 (d, J = 7.6 Hz, 4H), 7.22 (d, J = 7.6 Hz, 2H), 6.99 (d, J = 8.4 Hz, 1H), 5.27 (s, 2H), 5.01 (m, 1H), 4.68 (d, J = 5.6 Hz, 2H), 4.00 (s, 3H), 3.99 (s, 2H), 3.96 (s, 3H), 3.55 (s, 6H), 2.37 (s, 3H). ^{13}C NMR (125 MHz, $CDCl_3$) δ 157.8, 150.3, 149.6, 147.8, 144.9, 144.6, 137.0, 135.3, 133.8, 131.4, 128.8, 127.0, 122.6, 118.7, 113.9, 111.9, 111.3, 109.0, 101.7, 70.6, 64.6, 61.3, 56.7, 55.6, 53.4, 20.7. HRMS (EI) calcd for $C_{30}H_{33}O_7N$ 519.2252 $[M]^+$, found 519.2254.

3-(3-([1,1'-biphenyl]-4-ylmethoxy)-4-methoxyphenyl)-1-(2,2-dimethoxyethoxy)-7,8-dimethoxyisoquinoline(22l)

Compound **22l** was obtained from **10** (130 mg, 0.4 mmol) and (3-([1,1'-biphenyl]-4-ylmethoxy)-4-methoxyphenyl)boronic acid (160 mg, 0.48 mmol) by following a similar procedure as that for **22d**, it was purified by silica gel (PE: EA = 20:1) as a solid (119 mg, 51%). ^{1}H NMR (400 MHz, $CDCl_3$) δ 7.80 (d, J = 2.0 Hz, 1H), 7.71 (dd, J = 8.4, 2.0 Hz, 1H), 7.68 – 7.59 (m, 6H), 7.53 (d, J = 8.8 Hz, 1H), 7.50 (s, 1H), 7.45 (m, 2H), 7.37 (d, J = 7.6 Hz, 1H), 7.01 (d, J = 8.4 Hz, 1H), 5.35 (s, 2H), 5.01 (t, J = 5.6 Hz, 1H), 4.69 (d, J = 5.2

Hz, 2H), 4.00 (s, 3H), 3.99 (s, 3H), 3.98 (s, 3H), 3.54 (s, 6H). ^{13}C NMR (125 MHz, $CDCl_3$) δ 157.9, 150.3, 149.6, 147.7, 144.5, 140.4 (d, J = 14.4 Hz), 135.8, 135.3, 131.5, 128.2, 127.4, 126.9, 126.8, 126.6, 122.6, 118.9, 118.7, 113.9, 112.0, 111.31, 109.0, 101.8, 76.7, 70.5, 64.6, 61.3, 58.0, 56.6, 55.6, 53.4. HRMS (EI) calcd for $C_{35}H_{35}O_7N$ 581.2408 $[M]^+$, found 581.2417.

3-(3-((4-(tert-butyl)benzyl)oxy)-4-methoxyphenyl)-1-(2,2-dimethoxyethoxy)-7,8-dimet -hoxyisoquinoline(22m)

Compound **22m** was obtained from **10** (130 mg, 0.4 mmol) and (3-((4-(tert-butyl)benzyl)oxy)-4-methoxyphenyl)boronic acid (113 mg, 0.48 mmol) by following a similar procedure as that for **22d**, it was purified by silica gel (PE: EA = 20:1) as a solid (153 mg, 68%).^{1}H NMR (400 MHz, DMSO-d_6) δ 7.87 (s, 1H), 7.84 (d, J = 2.0 Hz, 1H), 7.74 – 7.64 (m, 3H), 7.44 (s, 4H), 7.09 (m, 1H), 5.18 (s, 2H), 4.95 (t, J = 5.6 Hz, 1H), 4.57 (d, J = 5.6 Hz, 2H), 3.93 (s, 3H), 3.83 (s, 6H), 3.43 (s, 6H), 1.29 (s, 9H). ^{13}C NMR (125 MHz, $CDCl_3$) δ 157.7, 150.2, 149.9, 149.7, 147.6, 144.9, 144.7, 138.7, 135.3, 131.2, 125.5, 124.9, 122.5, 118.7, 118.3, 113.8, 113.5, 111.6, 108.8, 101.8, 81.1, 72.3, 64.5, 61.3, 59.9, 56.7, 55.8, 53.5, 53.3, 37.7, 34.0, 30.9, 27.6, 22.1, 13.6. HRMS (EI) calcd for $C_{33}H_{39}O_7N$ 561.2721 $[M]^+$, found 561.2719.

1-(2,2-dimethoxyethoxy)-3-(3-((4-isopropylbenzyl)oxy)-4-methoxyphenyl)-7,8-dimeth-oxyisoquinoline(22n)

Compound **22n** was obtained from **10** (130 mg, 0.4 mmol) and (3-((4-isopropylbenzyl)oxy)-4-methoxyphenyl)boronic acid (135 mg, 0.48 mmol) by following a similar procedure as that for **22d**, it was purified by silica gel (PE: EA = 20:1) as a solid (163 mg, 74%). ^{1}H NMR (400 MHz, $CDCl_3$) δ 7.78 (d, J = 2.0 Hz, 1H), 7.70 (m, 1H), 7.53 (m, 1H), 7.49 (s, 2H), 7.43 (d, J = 8.8 Hz, 1H), 7.28 (s, 3H), 7.00 (d, J = 8.8 Hz, 1H), 5.26 (s, 2H), 5.02 (t, J = 5.2 Hz, 1H), 4.70 (d, J = 5.6 Hz, 2H), 4.00 (s, 3H), 3.99 (s, 3H), 3.96 (s, 3H), 3.55 (s, 6H), 2.94 (m, 1H), 1.28 (s, 3H), 1.26 (s, 3H). ^{13}C NMR (150 MHz, $CDCl_3$) δ 157.9, 150.3, 149.6, 148.1, 147.9, 144.6, 135.3, 134.1, 131.4, 127.3, 126.2, 122.6, 118.8, 118.6, 113.9, 111.7, 111.2, 109.0, 101.7, 70.6, 64.6, 61.4, 56.7, 55.6, 53.5, 33.4, 29.3, 23.6. HRMS (EI) calcd for $C_{32}H_{37}O_7N$ 547.2565 $[M]^+$, found 547.2565.

3-(3-((3,5-dimethoxybenzyl)oxy)-4-methoxyphenyl)-1-(2,2-dimethoxyethoxy)-7,8-dime -thoxyisoquinoline(22o)

Compound **22o** was obtained from **10** (98 mg, 0.3 mmol) and (3-((3,5-dimethoxybenzyl)oxy)-4-methoxyphenyl)boronic acid (114 mg, 0.36 mmol) by following a similar procedure as that for **22d**, it was purified by silica gel (PE: EA = 20:1) as a solid

(141 mg, 83%). ^{1}H NMR (400 MHz, $CDCl_3$) δ 7.74 (s, 1H), 7.55 (d, *J* = 8.0 Hz, 1H), 7.50 (d, *J* = 9.6 Hz, 1H), 7.46 (s, 1H), 7.43 (d, *J* = 9.2 Hz, 1H), 7.00 (d, *J* = 8.8 Hz, 1H), 6.71 (d, *J* = 2.0 Hz, 2H), 6.42 (s, 1H), 5.23 (s, 2H), 5.00 (t, *J* = 5.6 Hz, 1H), 4.69 (d, *J* = 5.2 Hz, 2H), 4.00 (s, 3H), 3.99 (s, 3H), 3.97 (s, 3H), 3.82 (s, 6H), 3.54 (s, 6H). ^{13}C NMR (125 MHz, $CDCl_3$) δ 160.5, 157.9, 150.3, 149.6, 147.7, 144.9, 144.6, 139.2, 135.3, 131.5, 122.5, 119.1, 118.7, 113.9, 112.1, 111.3, 109.0, 104.7, 101.7, 99.5, 70.8, 64.5, 61.3, 56.7, 55.6, 54.9, 53.4. HRMS (EI) calcd for $C_{31}H_{35}O_9N$ 565.2306 $[M]^+$, found 565.2299.

3.3 Characterizations of some representative compounds

Spectra of compound **23d**

^{1}H NMR spectrum

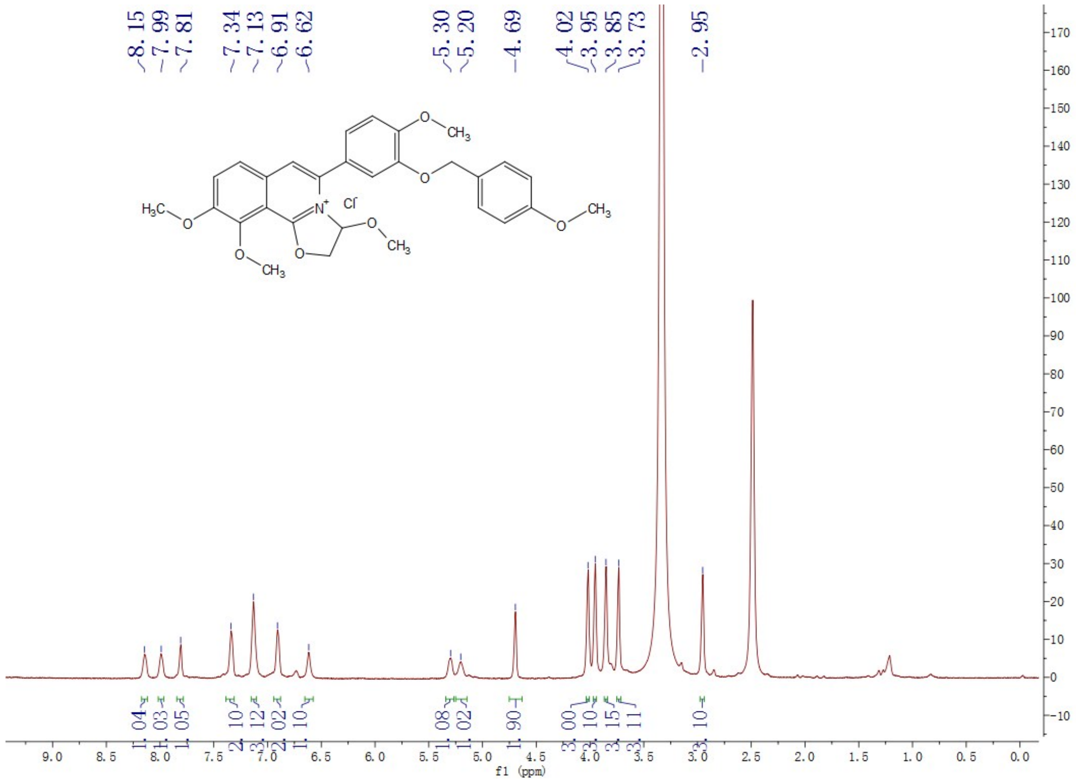

^{13}C NMR spectrum

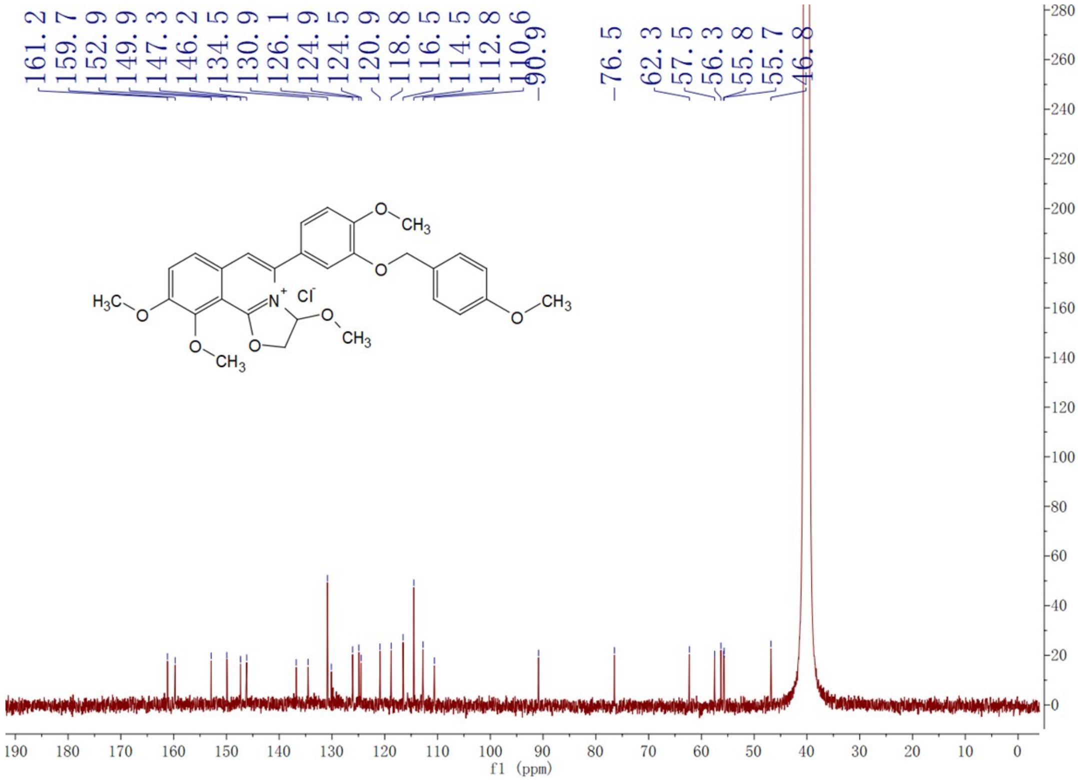

Spectra of compound **23e**

^{1}H NMR spectrum

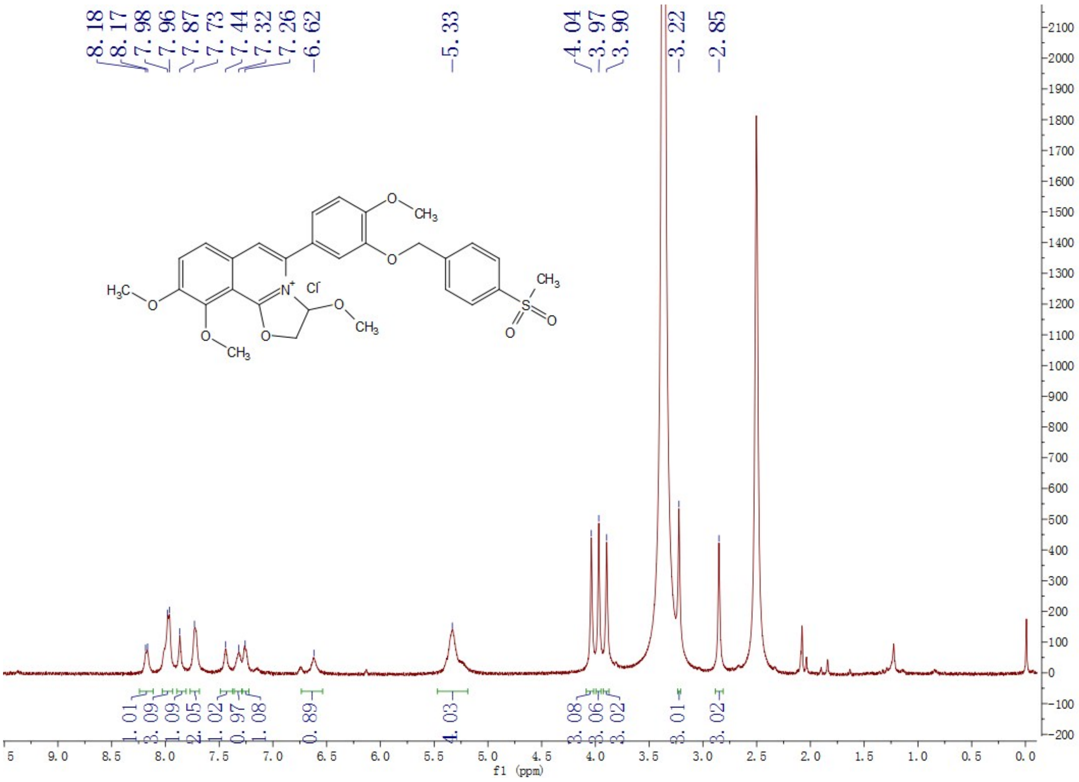

^{13}C NMR spectrum

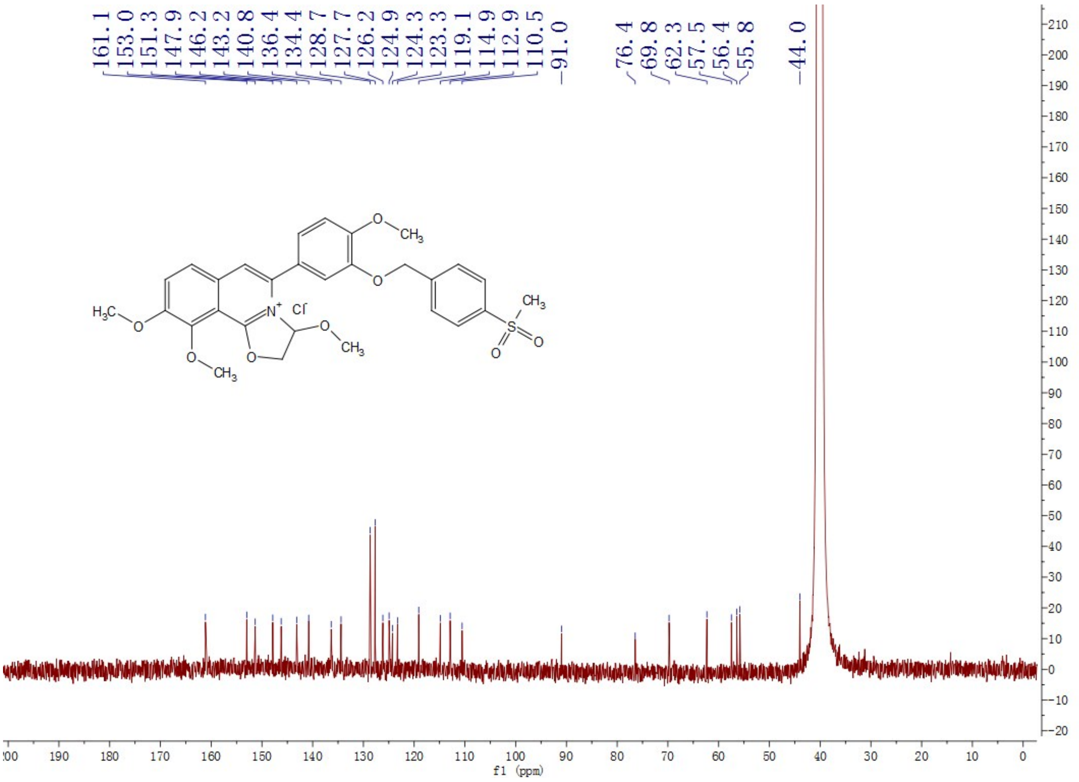

Spectra of compound **23f**

^{1}H NMR spectrum

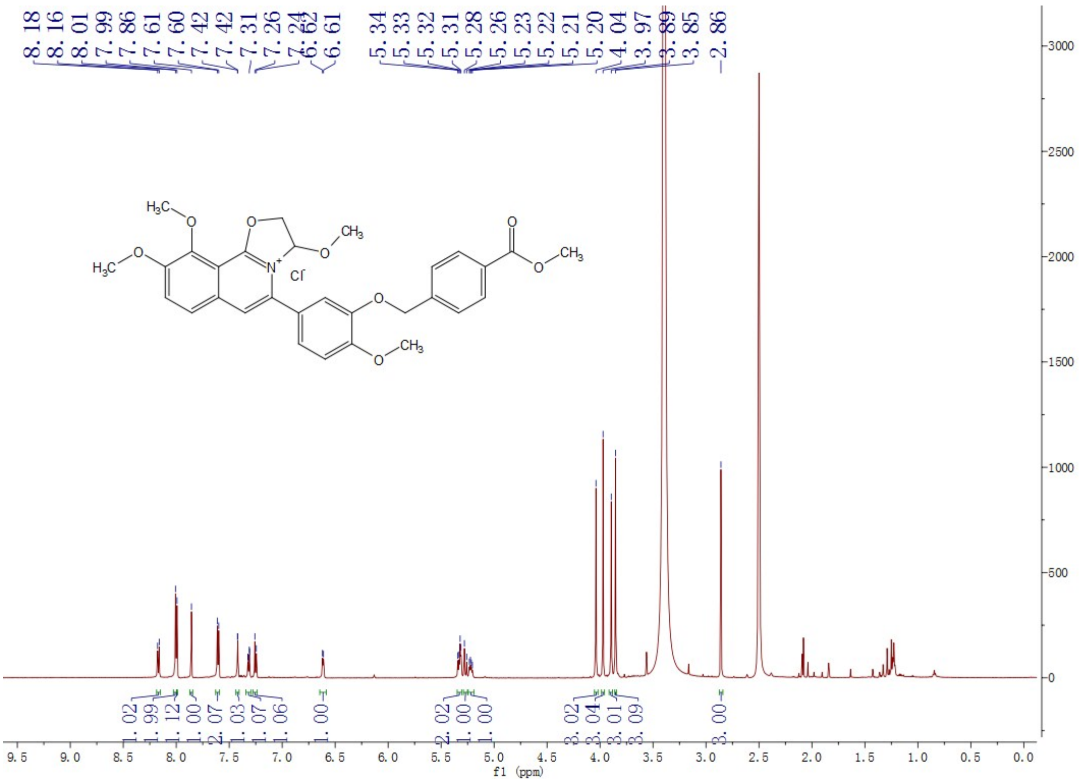

^{13}C NMR spectrum

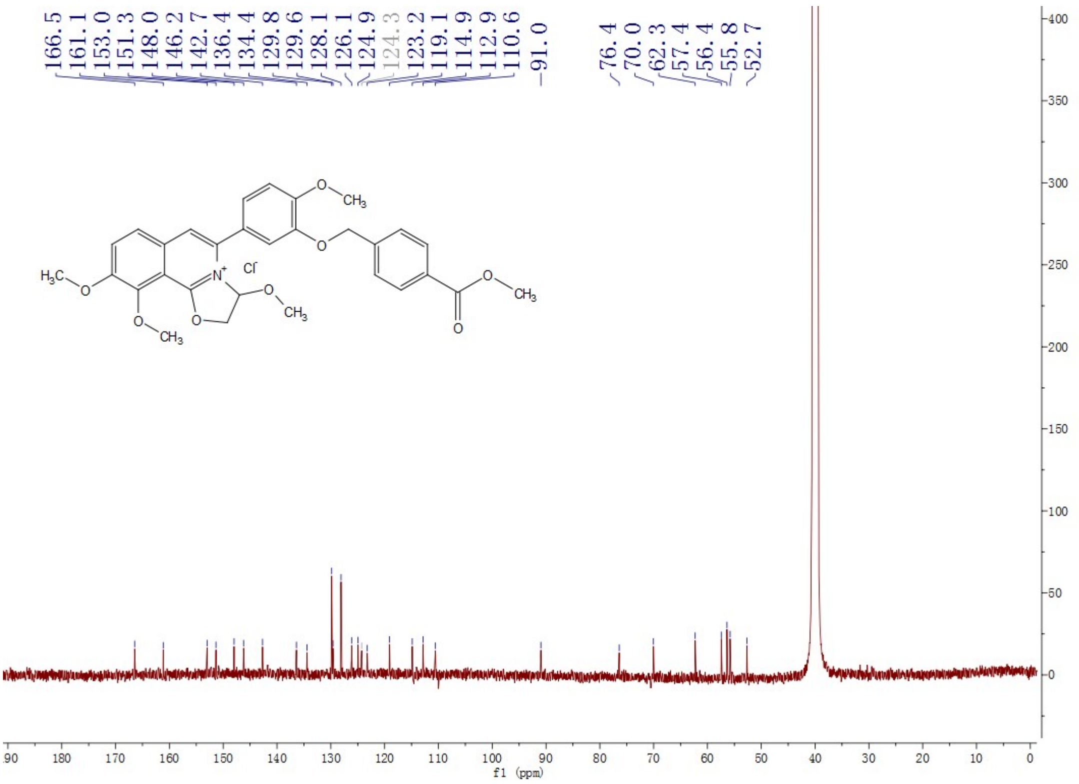

Spectra of compound **23g**

^{1}H NMR spectrum

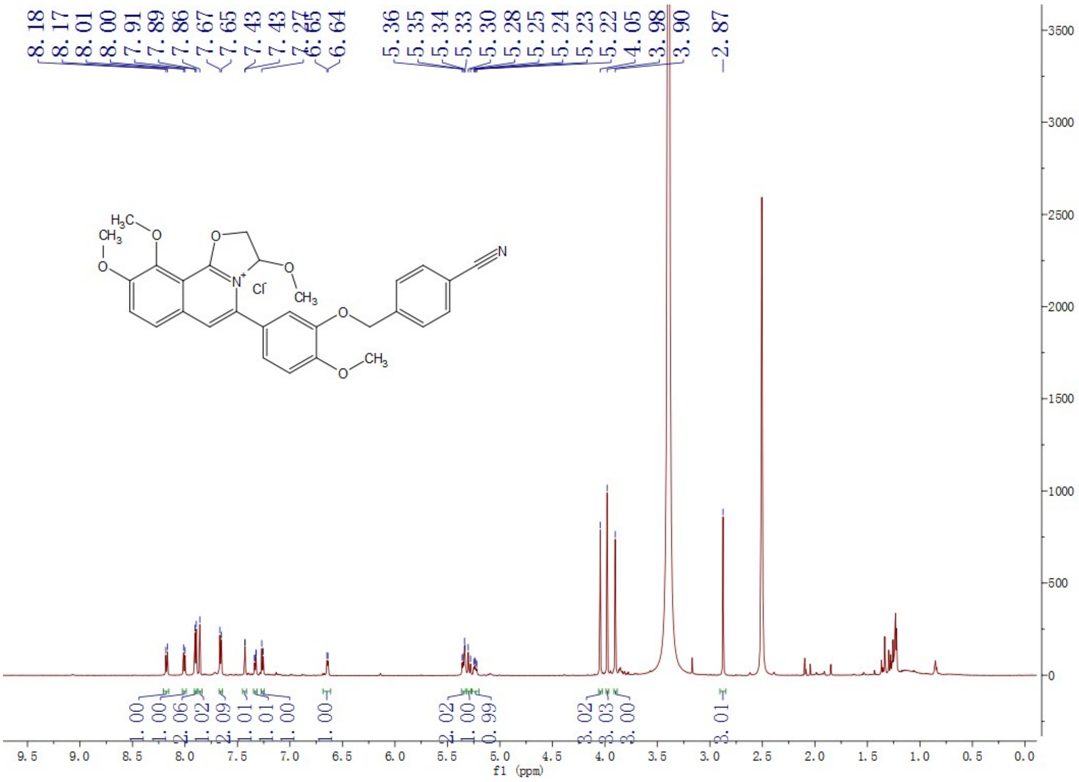

^{13}C NMR spectrum

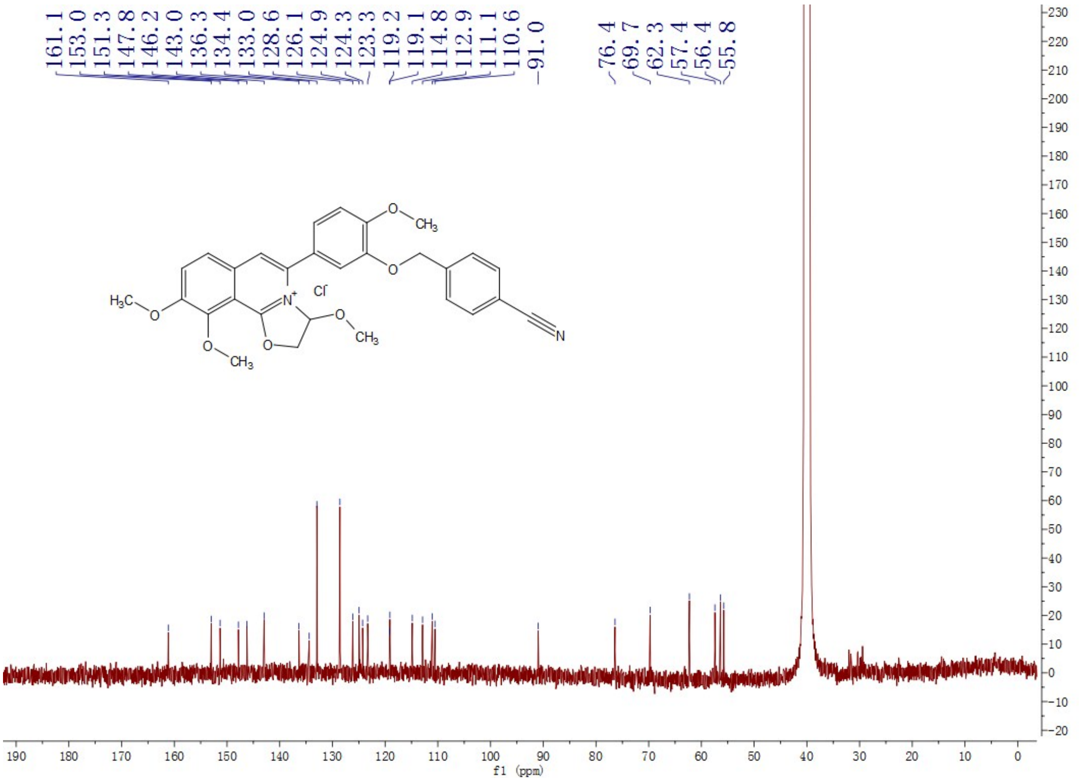

Spectra of compound **23h**

^{1}H NMR spectrum

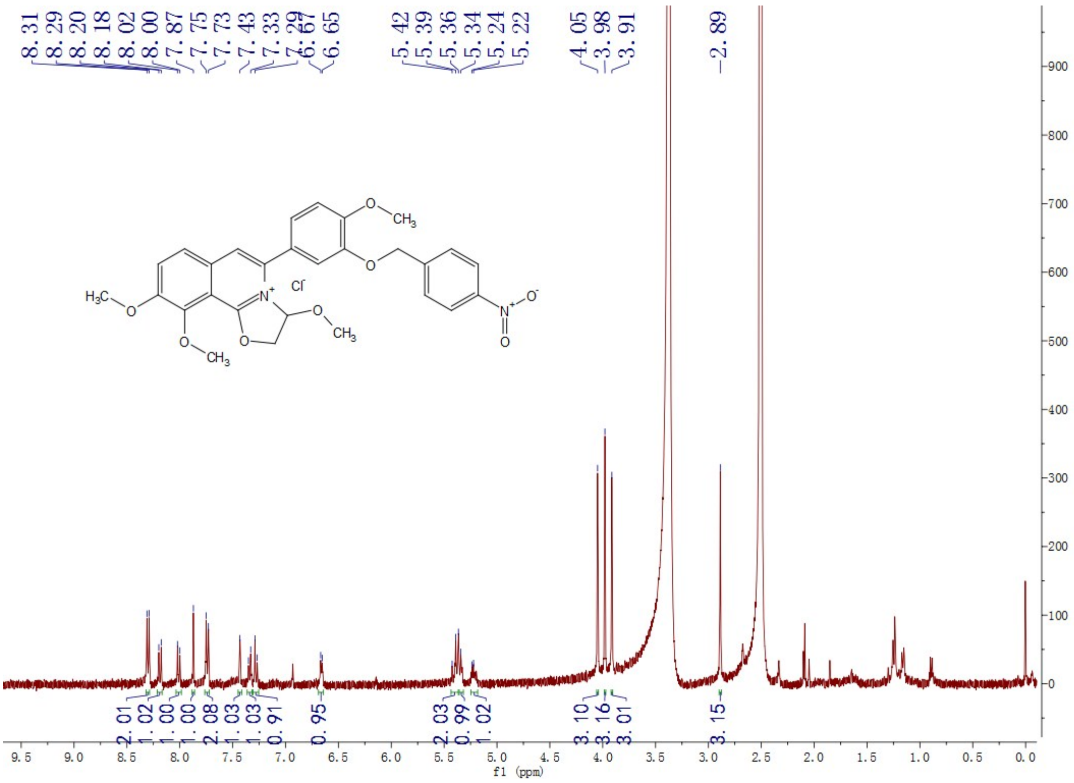

^{13}C NMR spectrum

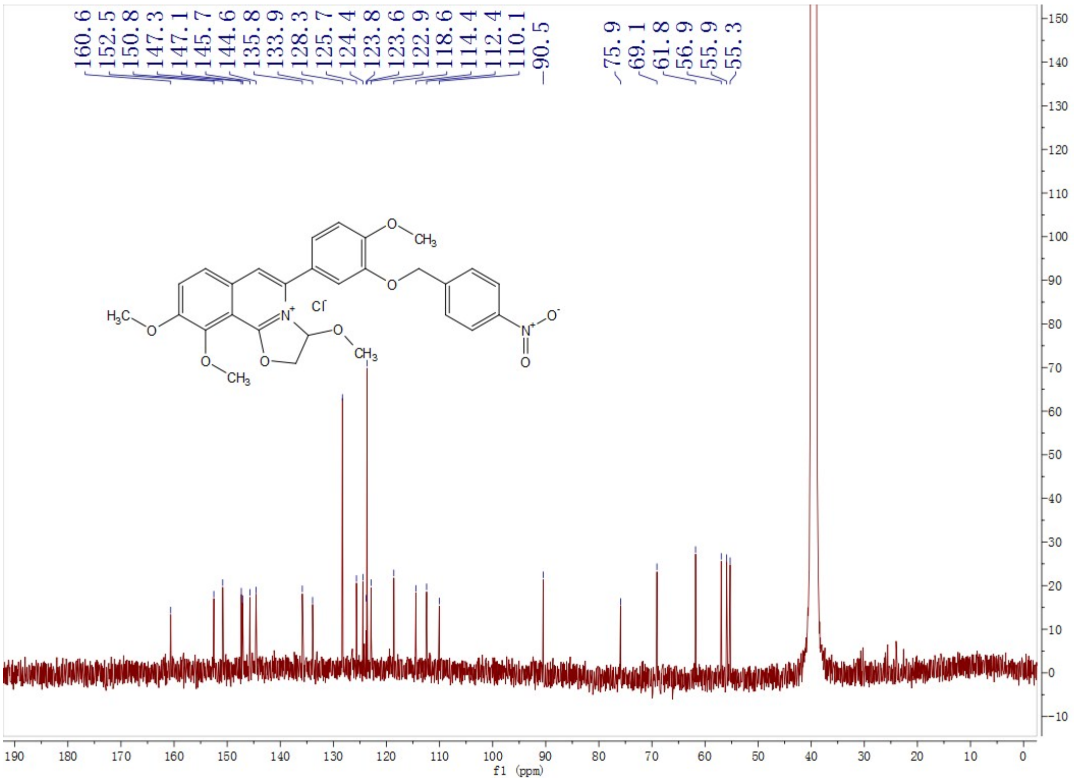

Spectra of compound **23i**

^{1}H NMR spectrum

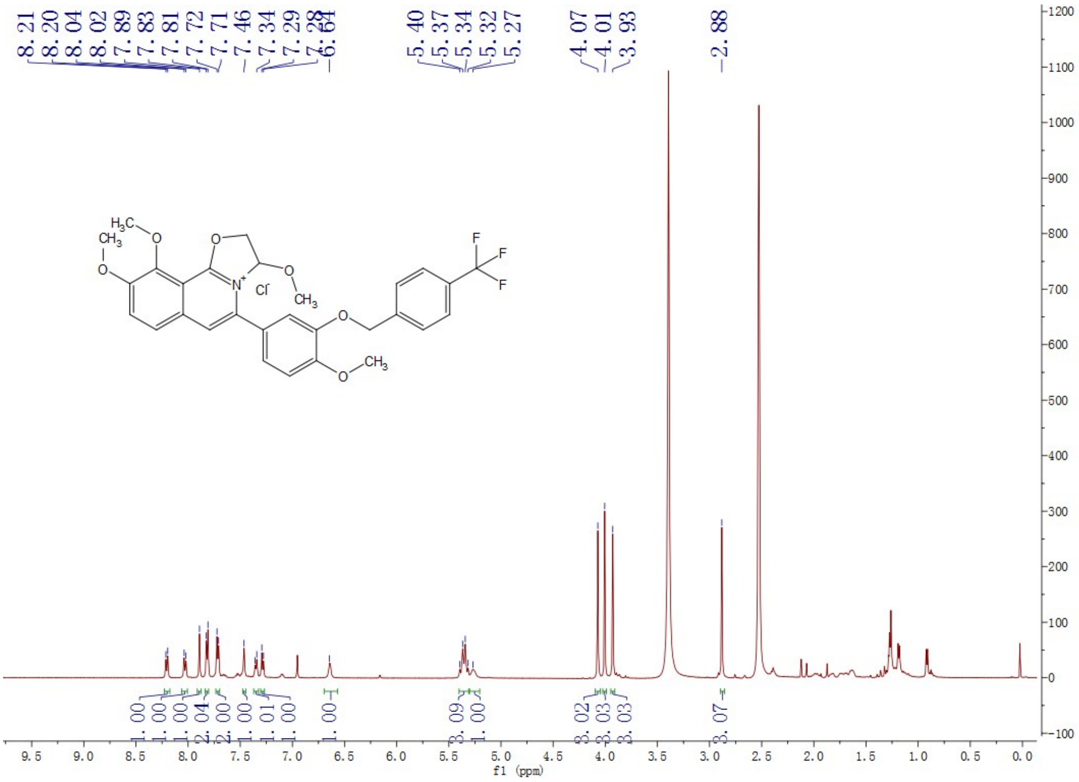

^{13}C NMR spectrum

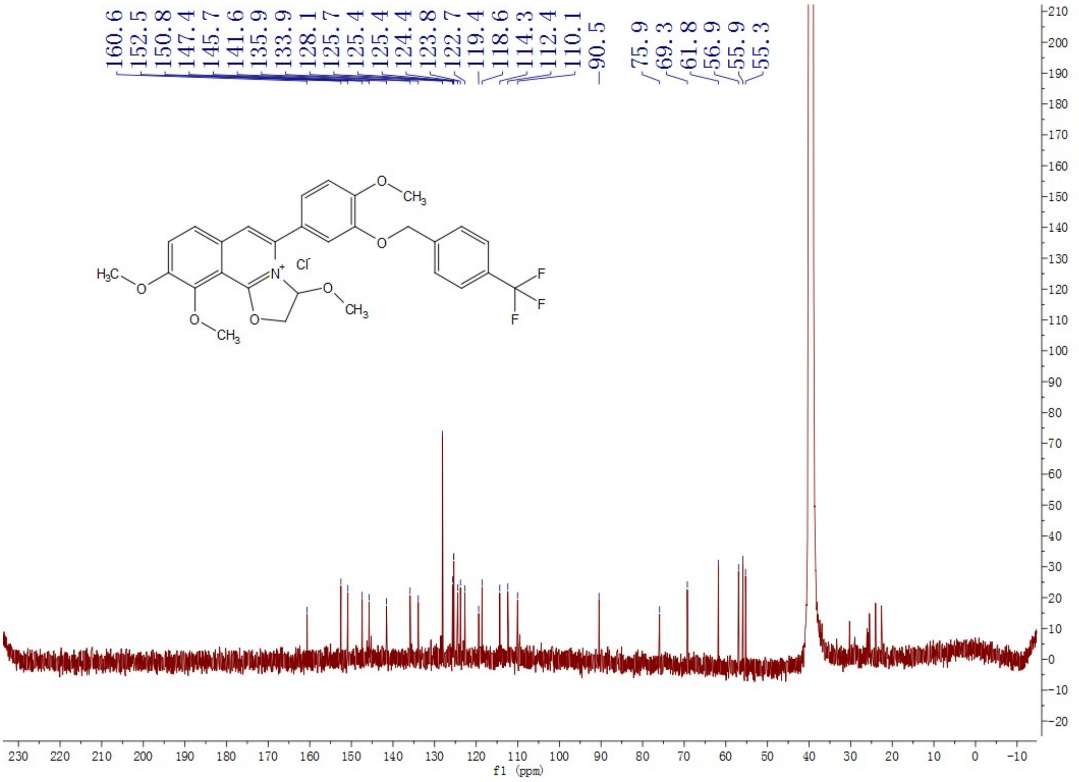

Spectra of compound **23j**

^{1}H NMR spectrum

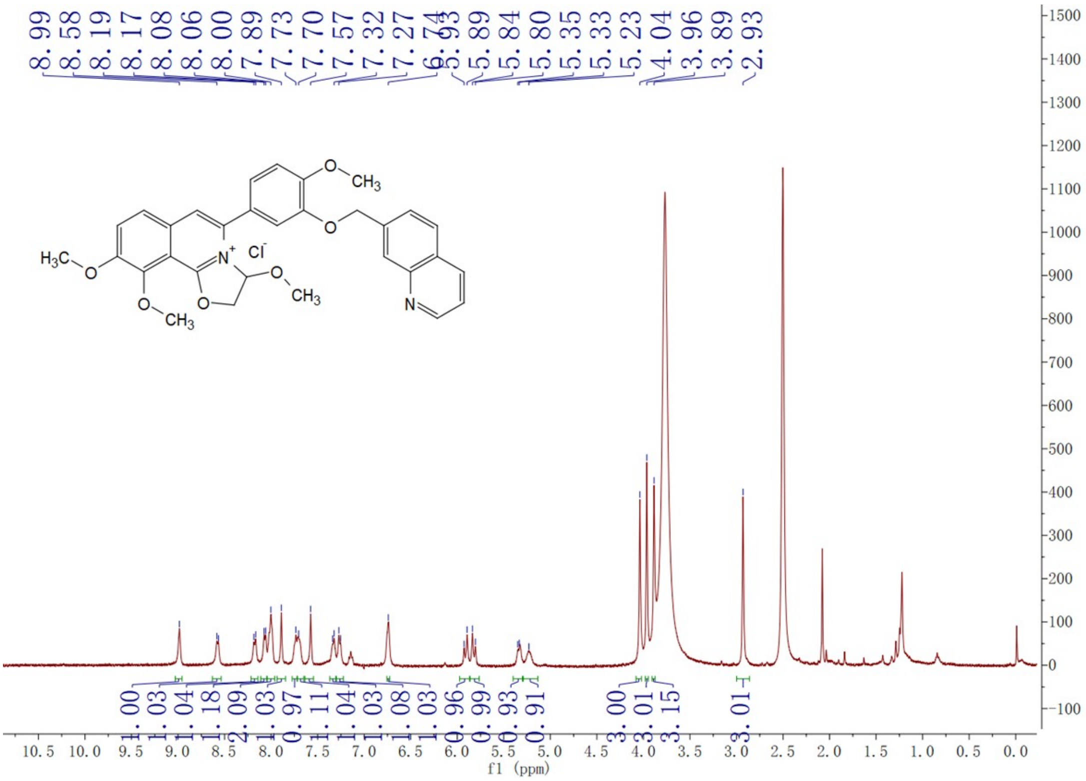

^{13}C NMR spectrum

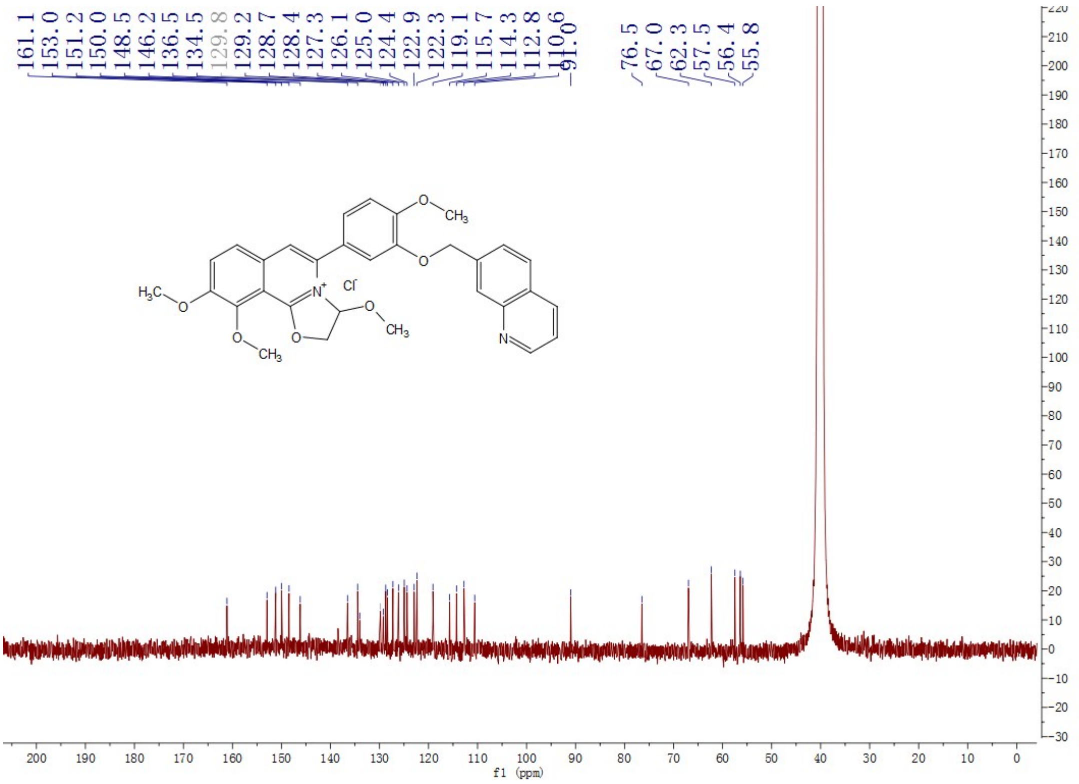

Spectra of compound **23k**

^{1}H NMR spectrum

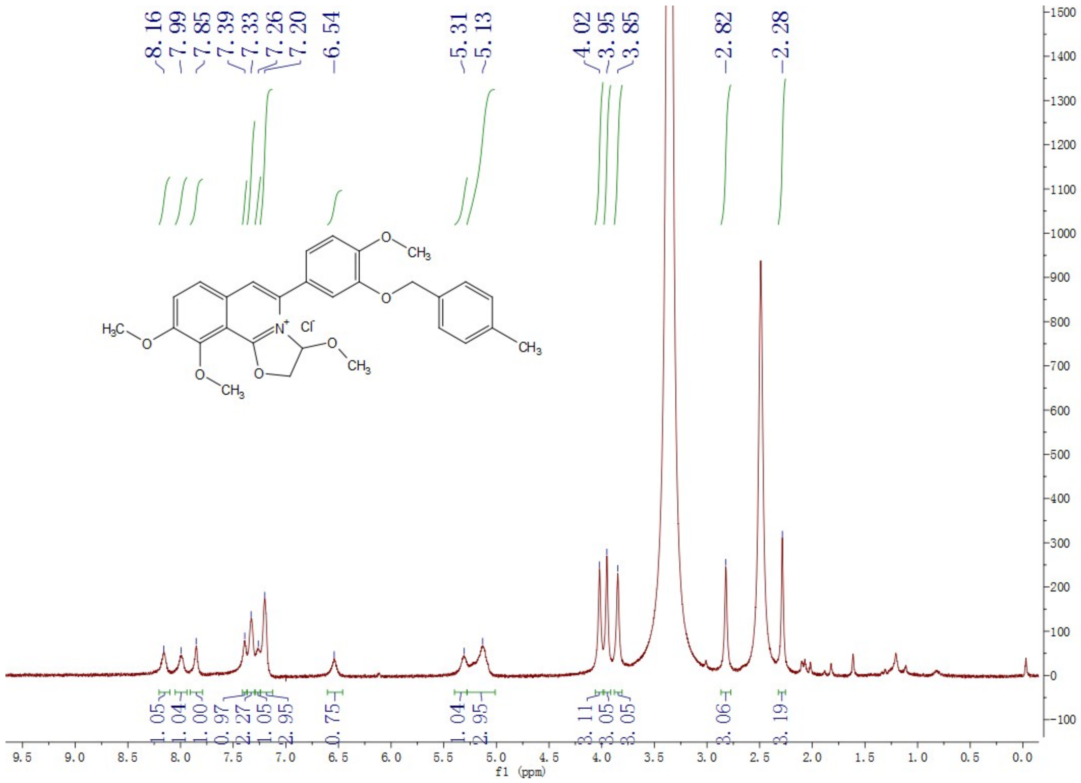

^{13}C NMR spectrum

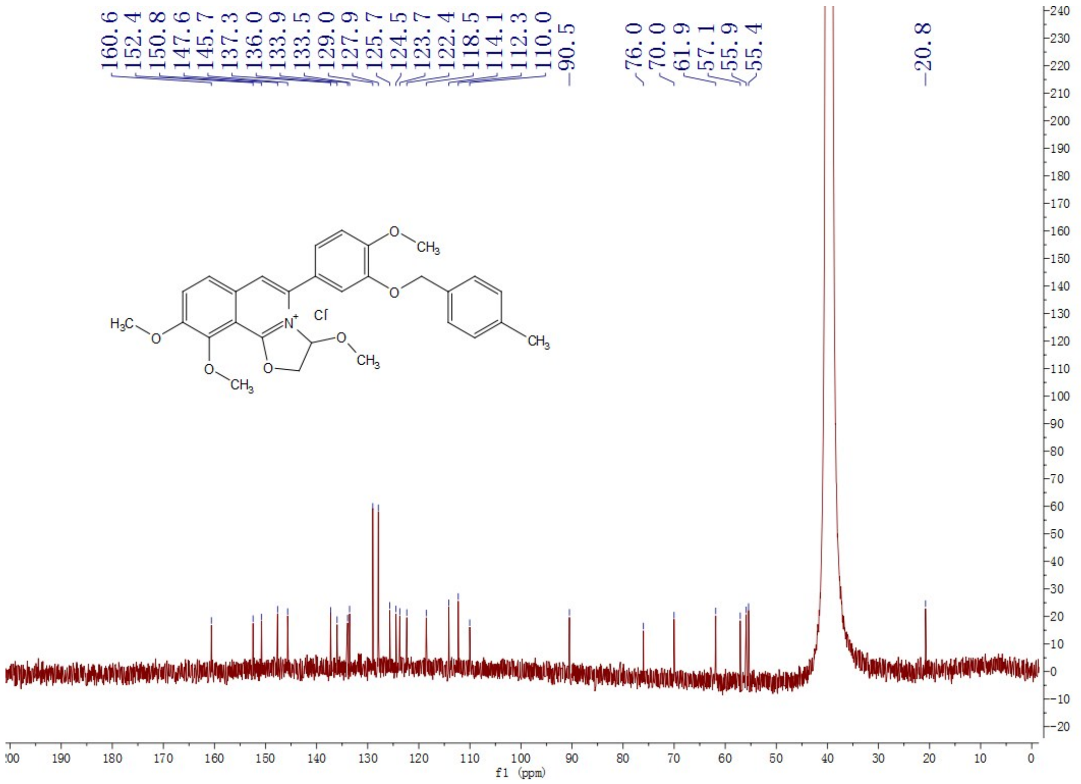

Spectra of compound **23l**

^{1}H NMR spectrum

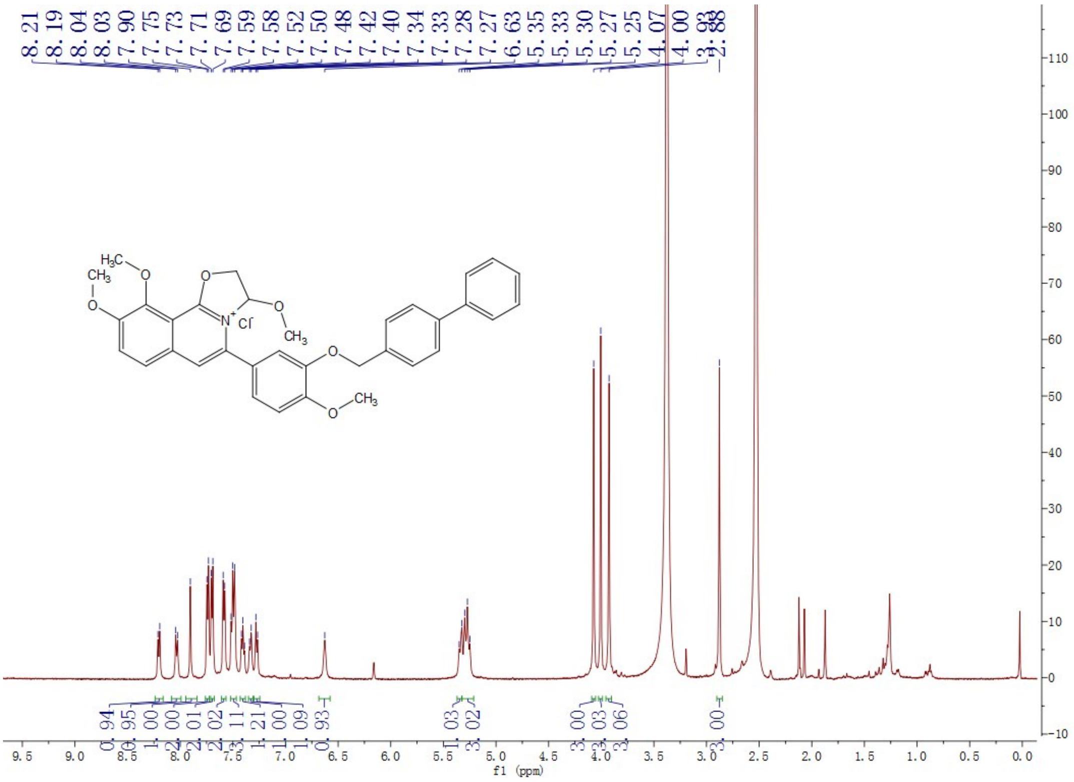

^{13}C NMR spectrum

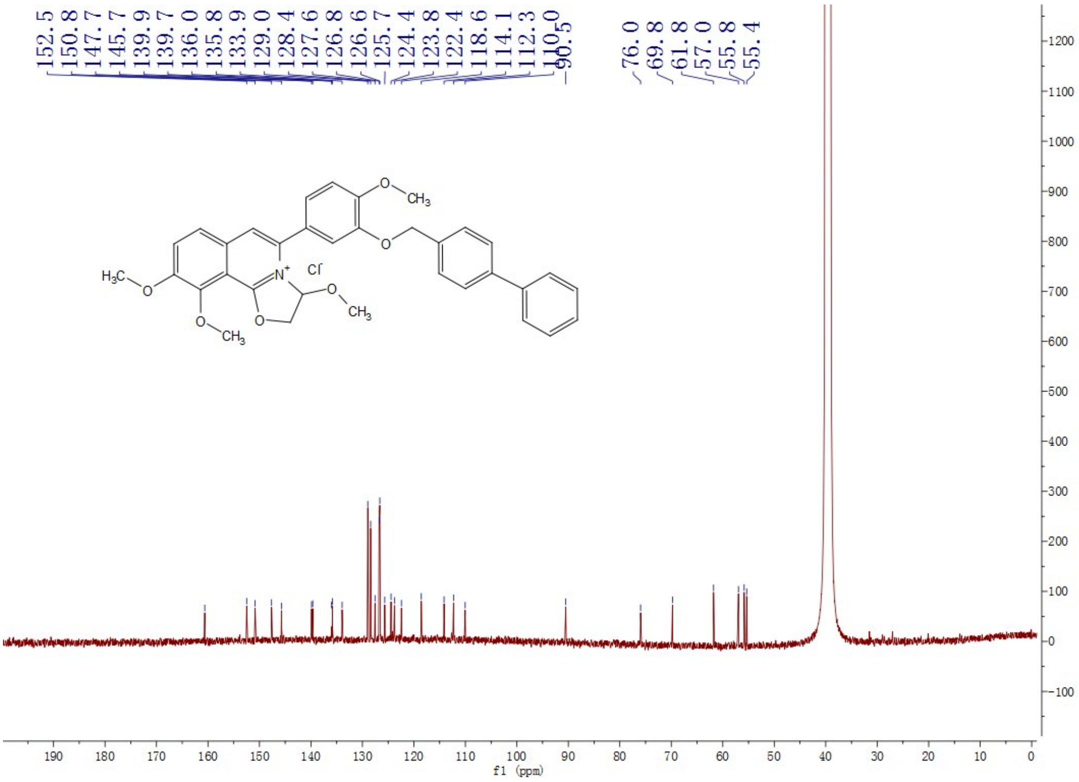

Spectra of compound **23m**

^{1}H NMR spectrum

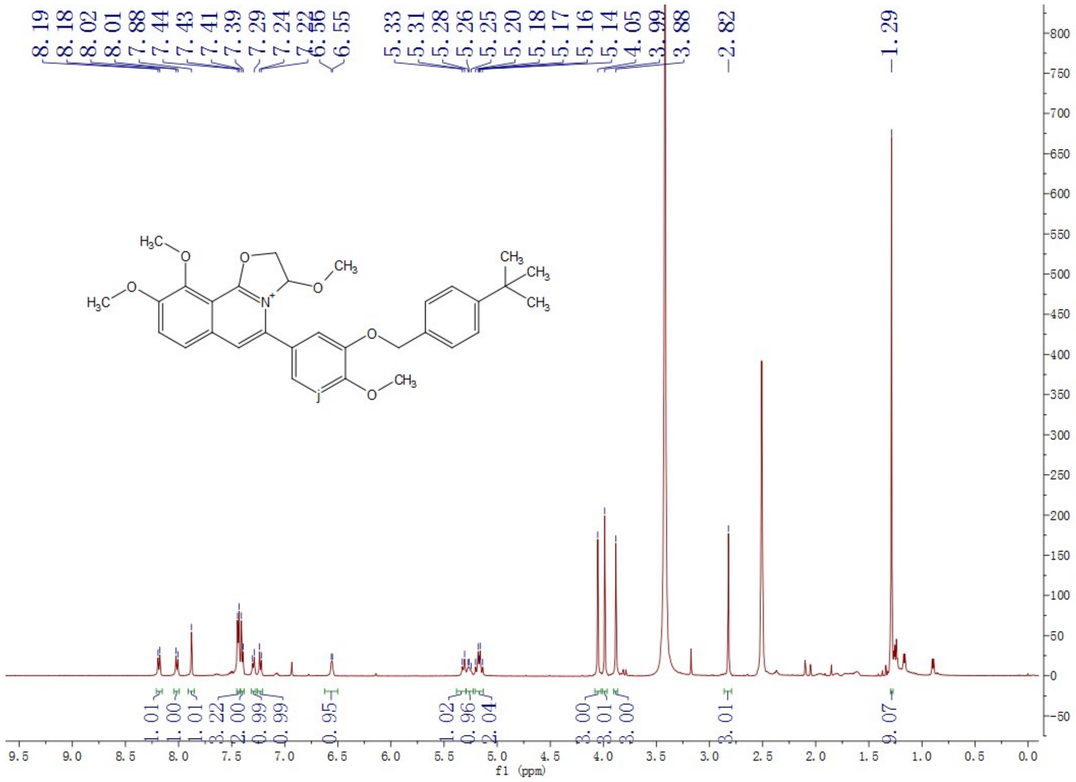

^{13}C NMR spectrum

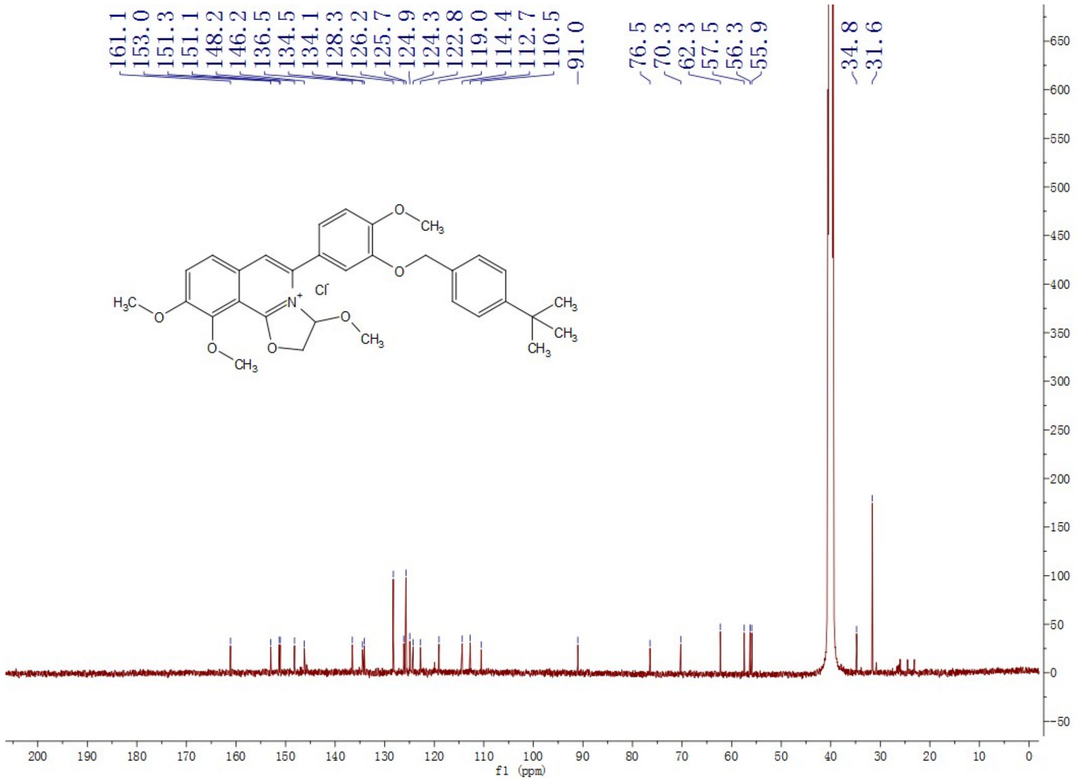

Spectra of compound **23n**

^{1}H NMR spectrum

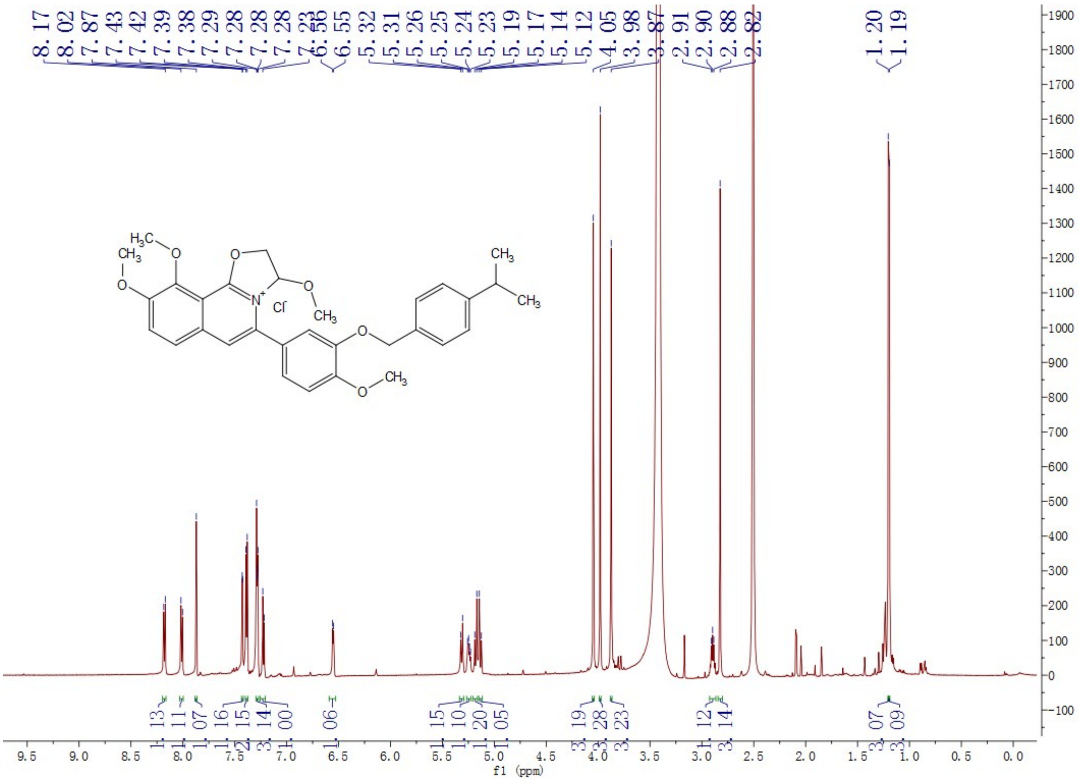

^{13}C NMR spectrum

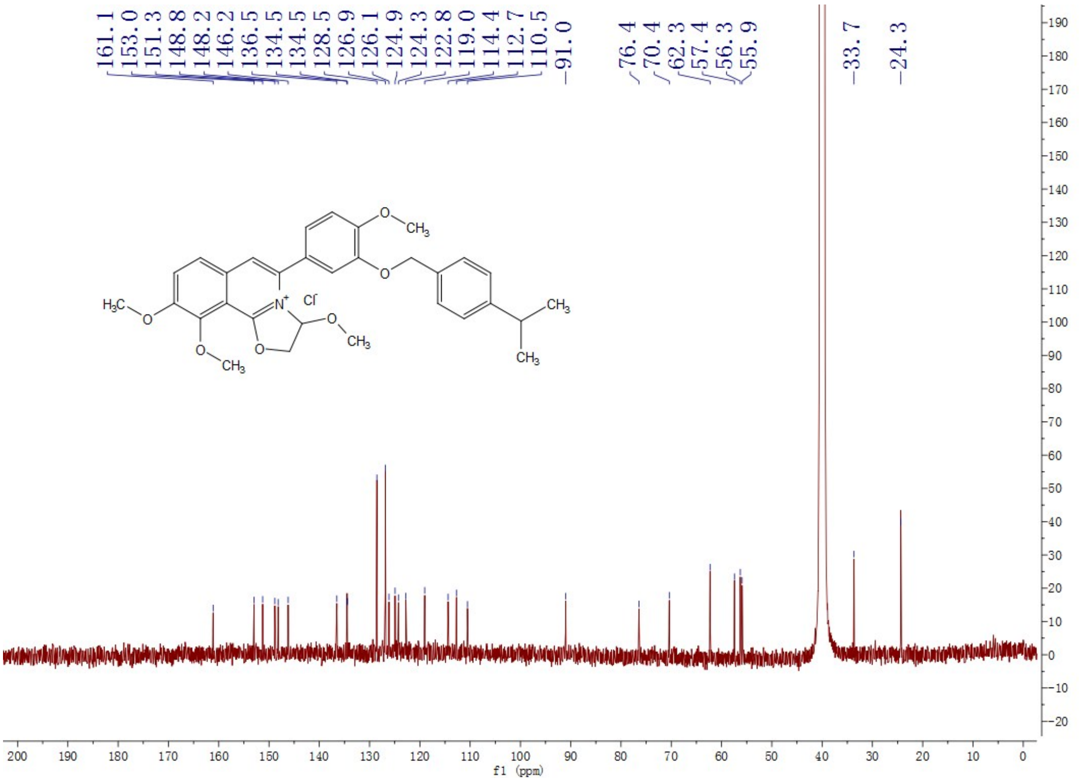

Spectra of compound **23o**

^{1}H NMR spectrum

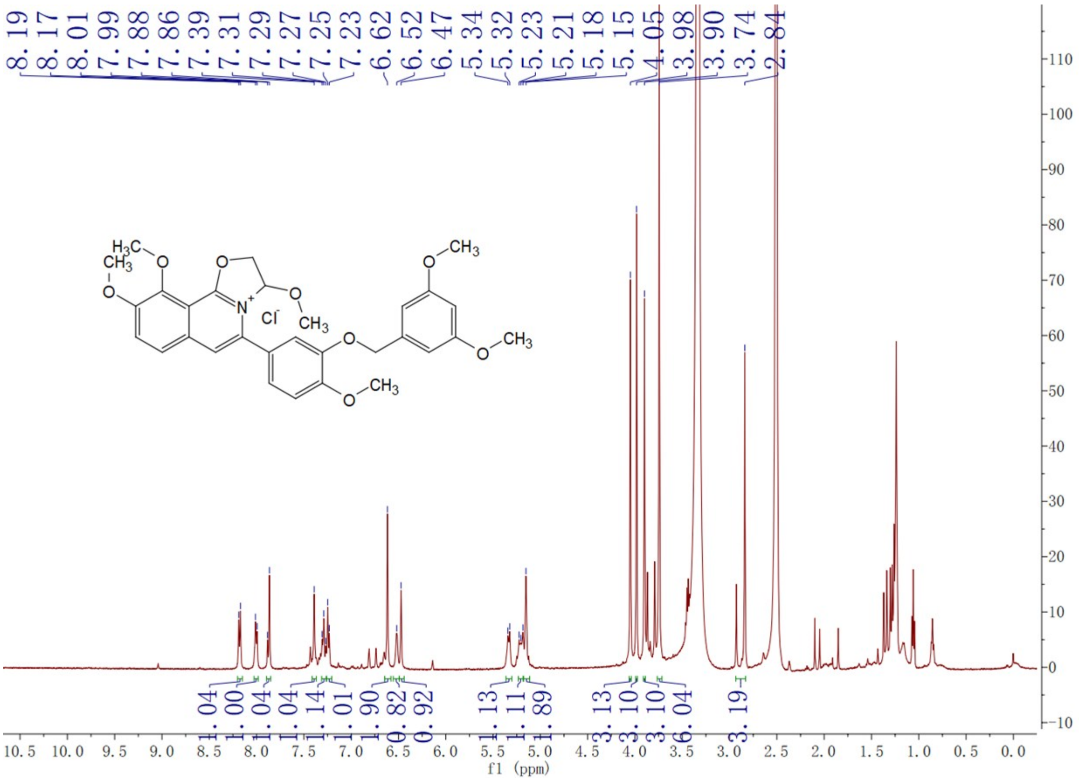

^{13}C NMR spectrum

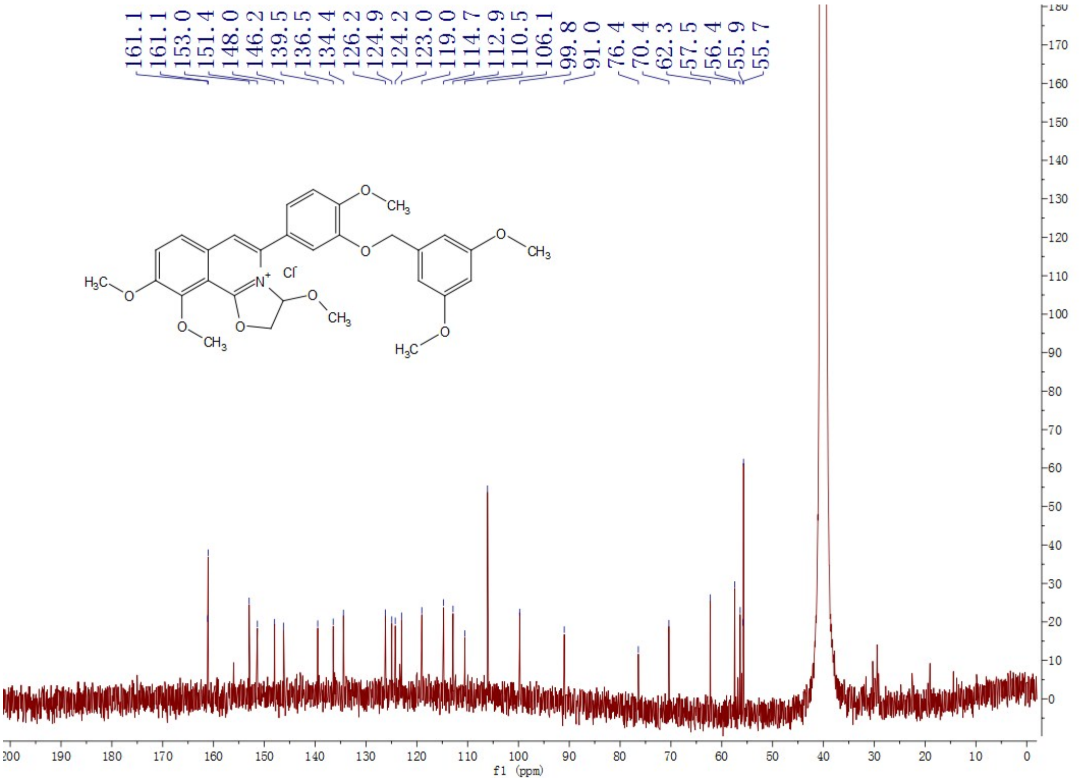

Supplement: RA-009-C9RA07457K-s002 [file RA-009-C9RA07457K-s002.pdf]
